# The Potential Roles of Host Cell miRNAs in Fine-Tuning Bovine Coronavirus (BCoV) Molecular Pathogenesis, Tissue Tropism, and Immune Regulation

**DOI:** 10.3390/microorganisms12050897

**Published:** 2024-04-30

**Authors:** Abid Ullah Shah, Maged Gomaa Hemida

**Affiliations:** Department of Veterinary Biomedical Sciences, College of Veterinary Medicine, Long Island University, Brookville, NY 11548, USA; abidullah.shah@liu.edu

**Keywords:** bovine coronavirus, BCoV, microRNAs, markers, pathogenesis, trophism, immune regulation

## Abstract

Bovine coronavirus (BCoV) infection causes significant economic loss to the dairy and beef industries worldwide. BCoV exhibits dual tropism, infecting the respiratory and enteric tracts of cattle. The enteric BCoV isolates could also induce respiratory manifestations under certain circumstances. However, the mechanism of this dual tropism of BCoV infection has not yet been studied well. MicroRNAs (miRNAs) are small non-coding RNAs that regulate gene expression and play a dual role in virus infection, mediating virus or modulating host immune regulatory genes through complex virus–host cell interactions. However, their role in BCoV infection remains unclear. This study aims to identify bovine miRNAs crucial for regulating virus–host interaction, influencing tissue tropism, and explore their potential as biomarkers and therapeutic agents against BCoV. We downloaded 18 full-length BCoV genomes (10 enteric and eight respiratory) from GenBank. We applied several bioinformatic tools to study the host miRNAs targeting various regions in the viral genome. We used the criteria of differential targeting between the enteric/respiratory isolates to identify some critical miRNAs as biological markers for BCoV infection. Using various online bioinformatic tools, we also searched for host miRNA target genes involved in BCoV infection, immune evasion, and regulation. Our results show that four bovine miRNAs (miR-2375, miR-193a-3p, miR-12059, and miR-494) potentially target the BCoV spike protein at multiple sites. These miRNAs also regulate the host immune suppressor pathways, which negatively impacts BCoV replication. Furthermore, we found that bta-(miR-2338, miR-6535, miR-2392, and miR-12054) also target the BCoV genome at certain regions but are involved in regulating host immune signal transduction pathways, i.e., type I interferon (IFN) and retinoic acid-inducible gene I (RIG-I) pathways. Moreover, both miR-2338 and miR-2392 also target host transcriptional factors RORA, YY1, and HLF, which are potential diagnostic markers for BCoV infection. Therefore, miR-2338, miR-6535, miR-2392, and miR-12054 have the potential to fine-tune BCoV tropism and immune evasion and enhance viral pathogenesis. Our results indicate that host miRNAs play essential roles in the BCoV tissue tropism, pathogenesis, and immune regulation. Four bovine miRNAs (miR-2375, bta-miR-193a-3p, bta-miR-12059, and bta-miR-494) target BCoV-S glycoprotein and are potentially involved in several immune suppression pathways during the viral infection. These miRNA candidates could serve as good genetic markers for BCoV infection. However, further studies are urgently needed to validate these identified miRNAs and their target genes in the context of BCoV infection and dual tropism and as genetic markers.

## 1. Introduction

Bovine coronavirus (BCoV) is a pneumotropic virus with a dual tropism in the respiratory and enteric tracts of the affected cattle [1,2]. BCoV causes several clinical syndromes in the affected animals, including calf diarrhea and winter dysentery, and contributes to developing the bovine respiratory disease complex (BRDC) [3,4,5]. BCoV is endemic in some cattle herds in various regions of the world, including North America [2,6,7,8,9,10,11,12,13]. BCoV is an enveloped positive-sense single-strand RNA virus, ranging from 65 to 210 nm in diameter [1]. The 5′ end of the BCoV genome is occupied with the nonstructural proteins (NSP1–NSP16). The 3′ end contains five major structural proteins (5′-Hemagglutinine esterase (HE), spike (S)-envelope (E), matrix (M), and the nucleocapsid (N)-3′) [14,15].

BCoV-S is the most crucial protein that shapes viral infection, pathogenesis, and immune regulation. The BCoV-S protein binds to multiple host receptors and is cleaved during the initial phase of viral infection by host cell proteases, enabling viral entry and disease progression [16,17,18]. The BCoV-S protein consists of the S1 (receptor-binding) subunit and the S2 (membrane-fusion) subunit [19]. The S1 subunit has two domains: an N-terminal domain (NTD) and a C-terminal domain (CTD) [20]. Both BCoV and the Human Coronavirus (HCoV)-OC43 recognize a sugar moiety called 5-N-acetyl-9-O-acetylneuraminic acid (Neu5,9Ac2) on the host cell surface of glycolipids or glycoproteins that play an essential role in virus–host receptor recognition [21,22]. The S1-NTD region of the BCoV spike protein is an essential receptor-binding domain that recognizes sugar moieties [23]. The S1/S2 furin cleavage site is usually cleaved by furin at the S1/S2 boundary to detach S1 from the S2 domain and control virus fusion and entry [24]. Receptor recognition and attachment are crucial and indispensable steps in viral infection and tropism [25]. Several related SARS-CoV-2 studies and other coronaviruses have demonstrated that blocking spike protein at certain critical regions can inhibit coronavirus infection [26]. However, research regarding BCoV within this context remains scarce.

MicroRNAs (miRNAs) are small, non-coding RNAs that target the 3′ UTR of the target mRNAs and inhibit their translation [27,28]. Several studies have shown that host miRNA-mediated RNA interference (RNAi) is essential in virus–host interactions [29,30,31]. Recent studies reported that, during viral infections, miRNAs emerged as a critical regulator either by suppressing or enhancing the expression of the targeted host gene(s) or the viral RNAs [32]. For example, miR-485 targets host RIG-I with a low abundance of H5N1 influenza virus while targeting the PB1 subunit of the viral genome (essential for viral replication) with an increased amount of H5N1, leading to marked inhibition of the virus replication [33]. Similarly, host miRNA targets the 3′UTR, receptor binding domain, and the structural and nonstructural proteins of SARS-CoV-2 and inhibits viral replication [34]. For example, the up-regulation of miR-2392 enhanced SARS-CoV-2 replication in the host [35], while miR-150-5p inhibited SARS-CoV-2 replication in the target cells in vitro [36]. In addition to their role in modulating the host genome, miRNAs regulate the viral/host tissue tropism upon infection. A recent study reported that pseudorabies virus (PRV) upregulates some key host miRNAs to evade the innate immune response and establish its tropism in the kidneys, spleen, lungs, and other tissues [37]. Therefore, miRNAs hold promise as valuable biomarkers and potential therapeutic agents against viral diseases of various species of animals and humans.

Understanding the complex interaction between viruses and their host transcription machinery is of fundamental importance. As one of its possible interactions with host cells, the SARS-CoV-2 virus induces specific mechanisms to inhibit the production of the host nuclear proteins, transcription factors (TFs), and host mRNAs. This nucleic acid degradation could be achieved through the interaction between host miRNAs and some important viral proteins [38,39]. The TFs are the host cytoplasmic proteins that bind to the promoter region of the DNA-binding domain of target genes and influence transcription mechanisms. Both host miRNAs and TFs interact during viral infection and affect cell functions like proliferation, differentiation, and apoptosis [40]. Several studies have explored the interplay between host miRNAs and TFs during SARS-CoV-2 infection [41], but their role in BCoV remains to be investigated.

Coronavirus (CoV) infections, in general, trigger the host cells to activate several immunogenic cytokines, such as IFNs or inducible cytokines known as suppressors of the cytokine signaling (SOCS) pathway [42]. SOCS1 and SOCS3 are well-known regulators of SOCS pathways, essential in neutralizing interferons [43,44]. Several studies reported that CoV infection activates SOCS and suppresses the host immune regulation [44,45]. Sultani et al. (2023) found that miR-155 is upregulated during SARS-CoV-2 infection and targets SOCS1 and inhibits the host Th17/Treg pathway [46].

On the other hand, type I IFN plays a vital role in activating host innate immune responses after external pathogenic recognition. CoVs produce a double-stranded RNA (ds-RNA) during viral replication, which is recognized by host pattern recognition receptors (PRRs) as a pathogen-associated molecular pattern (PAMP) [47]. The PAMPs are recognized by cytosolic retinoic acid-inducible gene I (RIG-I) [48,49,50] and promote type I IFN transcription [51,52]. After infection, viruses promote tissue tropism either directly, causing pathogenicity through their genomes, or indirectly, stimulating host miRNAs to escape the host immune mechanism.

In the current study, we used several in silico prediction tools to identify some host miRNAs that potentially target and influence the host immune regulatory genes and immune-suppressing genes in the context of BCoV infection. Furthermore, we explored the potential roles of some host miRNAs in binding to various regions across the BCoV genome, inhibiting viral replication, and thus fine-tuning its tissue tropism and pathogenesis. We also identified some potential miRNAs that could serve as biological and genetic markers for BCoV infection and potentially distinguish between the enteric and respiratory isolates of BCoV. The outcomes of this study will enrich our knowledge about bovine miRNA and BCoV host interaction.

## 2. Materials and Methods

### 2.1. BCoV Genome Sequences

We downloaded 18 full-length genome sequences of bovine coronavirus (BCoV) (10 representing the enteric isolates and eight representing the respiratory isolates) from the National Center for Biotechnology Information (NCBI) for our downstream analysis (Table 1). The BCoV isolates were selected from diverse global regions, considering both geographical location and the year of isolation. The mature bovine miRNA sequences were retrieved from the miRBase Release 22.1 (http://www.mirbase.org/ accessed on 30 January 2019) [53]. One thousand sixty-four mature bovine miRNAs were retrieved from the online miRNA database (miRBase database) (https://www.mirbase.org/summary.shtml?orgbta).

### 2.2. Multiple Sequence Alignment and Comparison of Genomes of BCoV (Enteric and Respiratory) Isolates

The multiple sequence alignment of the 18 full-length genome sequences of -BCoV was conducted by snapgene 6.0.2 (http://www.snapgene.com) using the MUSCLE alignment method between various BCoV genome (enteric and respiratory) isolates.

### 2.3. Identification of Bovine miRNAs Targeting Host Genes and miRNAs Targeting Host Transcription Factors in Specific Tissues

The bovine miRNAs targeting host genes prediction was performed via miRWalk and TargetScan 8.0 [54,55]. miRWalk is an online bioinformatic tool that produces the integrated network of relationships between miRNAs–genes–pathways and miRNAs–genes–disorder interactions. The miRWalk prediction presenting the network interaction of miRNAs and genes was obtained from miRWalk. The RumimiR online bioinformatic tools were used to identify miRNAs based on animal status, tissue origins, and experimental conditions from publications [56]. The generated figures illustrating the network interaction of miRNAs and their potential target genes, transcription factors, and tissues were produced by Cytoscape version 3.10.0 [57].

### 2.4. Host miRNAs Potentially Targeting BCoV Genome Sequences at Various Locations

We used a combination of different bioinformatic tools to identify potential bovine miRNAs targeting BCoV. We used the genome sequence of the Mebus (Accession Number: U00735) isolate as a reference strain for the enteric BCoV isolates [58]. To identify potential miRNAs binding to the Mebus isolate of BCoV, we used online software RNA22 v2 (https://cm.jefferson.edu/rna22/). We selected the binding miRNAs based on the binding energy and the 6–8 bp (6mer–8mer) binding at the seed region of miRNAs. The selection criteria for the potential miRNA candidates were based on several parameters: the minimum free energy and the complementarity between the miRNA seed region and the viral/host gene binding site (specified as nucleotide 2-8 on candidate miRNAs) of a minimum of six nucleotide-binding mRNA candidates and the BCoV target gene.

### 2.5. Functional Enrichment Analysis of Genes Targeted by the Relevant miRNA Candidates

To identify the biological significance of the selected bovine miRNAs and their impacts on the differentially expressed genes (DEGs), Gene Ontology (GO) and Kyoto Encyclopedia of Genes and Genomes (KEGG) enrichment analyses in the context of BCoV infection were performed via miRWalk (https://cm.jefferson.edu/rna22/) [54]. The pathway enrichment category plots for KEGG and GO were plotted by (https://www.bioinformatics.com.cn/en), a free online platform for data analysis and visualization.

## 3. Results

### 3.1. Bovine miRNAs Targeting the BCoV Genome at Various Locations

**Figure 1:**
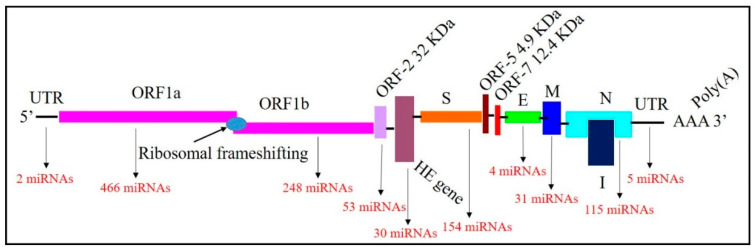
Mapping of the Bovine Coronavirus (BCoV) genome and binding miRNAs. The genome includes 5′ untranslated region (UTR), Open Reading Frame (ORF)1a, ORF1b, ORF-2, Hemagglutinin Esterase (HE), Spike (S), ORF5, ORF7, Envelope (E), Membrane (M), Nucleocapsid (N), 3′UTR and Poly(A) tail in order. The numbers of miRNAs binding with each gene are shown in red.

This study used several bioinformatic approaches to identify potential miRNA candidates targeting BCoV genomes at various locations. Our initial screening identified 1148 potential binding sites of several host bovine miRNA candidates in multiple locations across the BCoV genomes (Supplementary File S1). The numbers of bovine miRNAs that bind to the ORF1a, ORF1b, and spike genes are 466, 248, and 154, respectively (Figure 1). Notably, five host miRNAs (bta-miR-2349, bta-miR-211, bta-miR-204, bta-miR-12004, and bta-miR-760-3p) target the 3′ untranslated region (UTR) of BCoV (Figure 1, Supplementary File S1).

### 3.2. Bovine Host Cellular miRNAs Targeting the BCoV-S Protein

Our data show that 154 bovine miRNAs have potential target sites at several locations across the BCoV-S protein (Figure 1). To identify the most potential miRNA candidates, we selected the miRNA candidates that are potentially bound to the most sensitive sites of BCoV-S protein (i.e., S1-NTD sugar-binding sites, S1/S2 Furin cleavage site, GxCx Motif, and conserved S2 monomers of the BCoV spike protein), which are involved in virus attachment into the host cell and the downstream replication and pathogenesis (Table 2).

**Figure 2:**
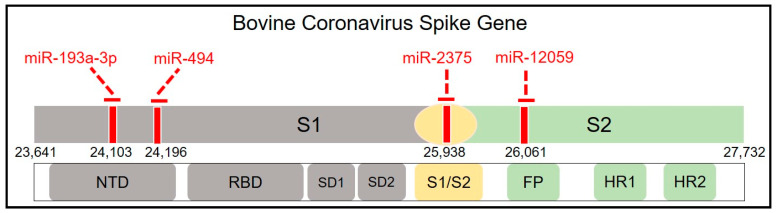
Mapping the host cell miRNAs targeting sites spanning the BCoV-S glycoprotein. Schematic representation of the BCoV spike glycoprotein (from left to right): N-terminal domain (NTD), Receptor-Binding Domain (RBD), Subdomain (SD) 1, SD2, S1/S2 Furin cleavage site, Fusion Peptide (FP), Heptad Repeat (HR) 1, and HR2. The red lines indicate the binding sites of miRNAs.

**Figure 3:**
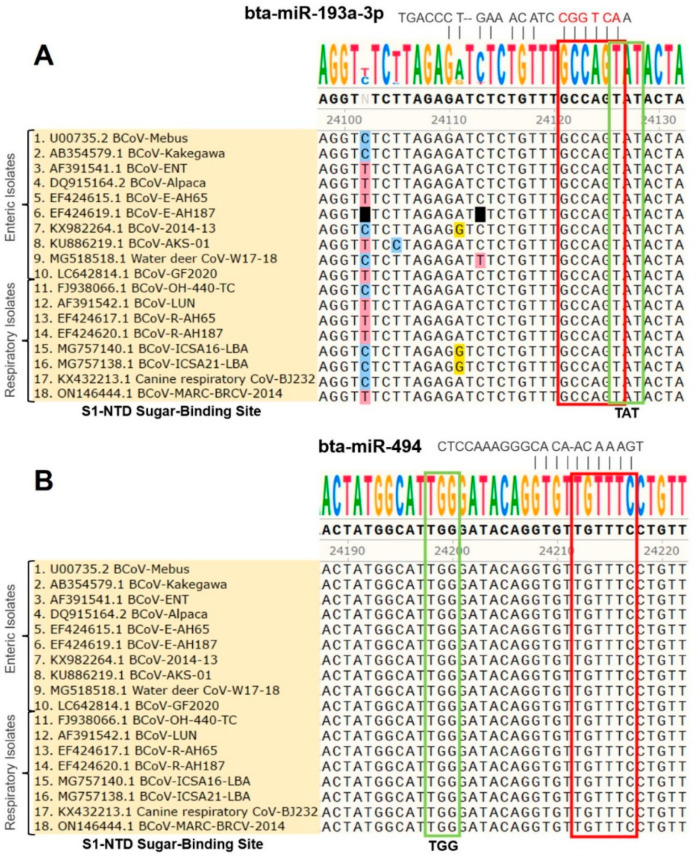
Multiple sequence analysis of the BCoV-S1-NTD sugar-binding site: (**A**) Bovine miR-193a-3p binding to the S1-NTD sugar-binding site tyrosine-162 residue (TAT) of spike gene of BCoV. (**B**) Bovine miR-494 binding to the S1-NTD sugar-binding site histidine-185 residue (TGG) of spike gene of BCoV. The green box shows the exact S1-NTD sugar-binding site. The red box shows the candidate miRNAs’ seed region with complementary binding sites in BCoV-S glycoprotein.

S1-NTD is a crucial receptor-binding site of the BCoV-S protein in the host cell [23]. The S1/S2 furin cleavage site promotes virus entry and enhances replication. We found that both bta-miR-193a-3p and bta-miR-494 potentially synergistically target the S1-NTD sugar-binding sites of the BCoV-S protein. Meanwhile, bta-miR-2375 potentially targets the S1/S2 Furin cleavage site, while bta-miR-12059 has a potential target site within the conserved S2 monomer region (Figure 2 and Figure 3 and Table 3).

bta-miR-494 has three other potential binding sites in the BCoV genome at ORF1a, ORF1b, and the S1 region. Meanwhile, bta-miR-2375 potentially binds to two other sites at ORF1a and the spike gene’s S1 region (Supplementary File S1). The targeting by bovine miRNAs could influence the spike protein functions, inhibiting BCoV replication.

### 3.3. Differential Targeting of Some Bovine Host Cell miRNA Candidates to the Genomes of BCoV (Enteric/Respiratory) Isolates

**Figure 4:**
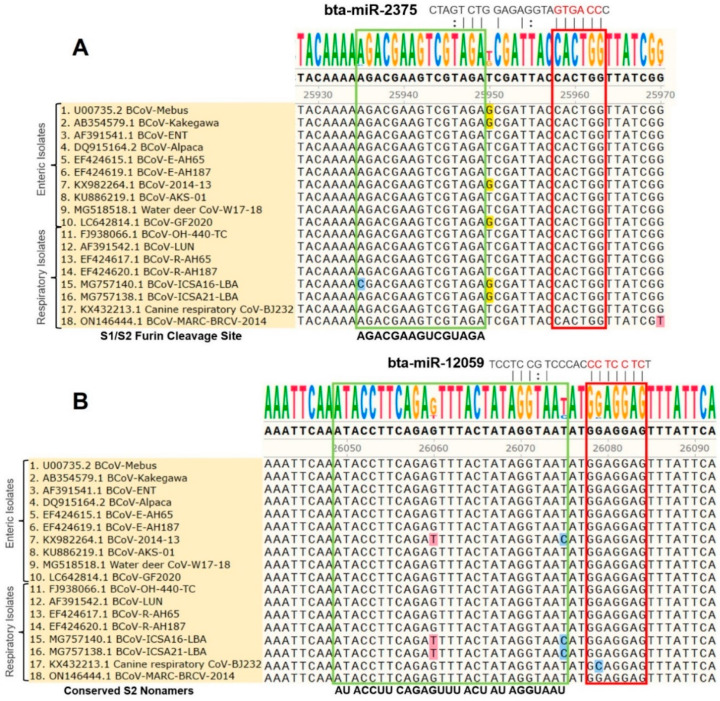
Multiple sequence analysis of the BCoV-S1/S2 Furin cleavage sites and the conserved S2 nonamer site: (**A**) Bta-miR-2375 binding to the S1/S2 Furin cleavage site of BCoV-S glycoprotein; (**B**) Bta-miR-12059 binding to the conserved S2 nonamer site of spike gene of BCoV. The green boxes show the S1/S2 Furin cleavage site and the conserved S2 nonamer site. The red boxes show miRNAs’ seed region binding sites with the BCoV-S genes.

The multiple sequence analysis (MSA) was performed on the 18 full-length BCoV genome sequences from enteric and respiratory isolates to map the homology and targeting of the chosen miRNA candidates. Results showed that bta-miR-193a-3p potentially binds to the region, including the tyrosine-162 residue of the S1-NTD sugar-binding site. This binding site was highly conserved among all of the selected isolates of BCoV (Figure 3A). Meanwhile, the binding of bta-miR-494 to another residue of the S1-NTD sugar-binding site was also conserved among all of the tested BCoV isolates (Figure 3B). Furthermore, the bta-miR-2375 binding to the S1/S2 Furin cleavage site was also conserved among all of the 18 BCoV isolates (Figure 4A). Similarly, bta-miR-12059 potentially binds to the conserved S2 nonamer region of the BCoV-S glycoprotein, which was conserved in all analyzed sequences except for the canine respiratory coronavirus isolate (Figure 4B).

### 3.4. Potential Impacts of Host Cell miRNAs on Viral Replication through Targeting Some Host Signal Transduction Pathways in the Context of BCoV Infection

**Figure 5:**
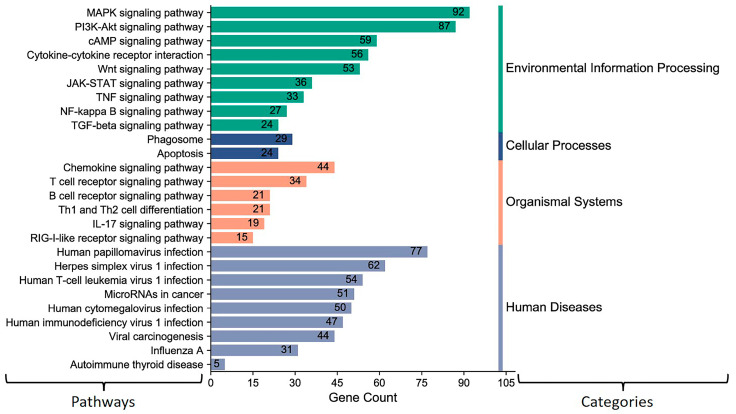
KEGG pathway enrichment analysis for some selected (miR-193a-3p, miR-494, miR-2375, and miR-12059) key host cell miRNAs: The number on the peak of each bar shows the gene numbers in specific pathways targeted and differently expressed by these miRNAs. The column on the left shows the target pathways. The column on the right side indicates KEGG pathways assigned by different categories. The MiRWalk analysis and the pathway enrichment category plots were plotted by https://www.bioinformatics.com.cn/en accessed on 31 January 2021, a free online platform for data analysis and visualization.

The above results showed that miR-2375, miR-193a-3p, miR-12059, and miR-494 target BCoV-S protein. Consequently, we aimed to elucidate the role of these miRNAs in host signal transduction pathways. The KEGG gene enrichment analysis was categorized into groups based on pathways involved in environmental information processing, cellular processing, organismal systems, and human diseases (Figure 5). The highest differentially expressed genes (DEGs) were observed in MAPK and PI3K-AKT pathways, followed by cytokine–cytokine receptor interaction, Wnt, and JAK/STAT signaling pathways (Figure 5). Furthermore, these miRNAs also target genes involved in the host receptor signaling pathways, including chemokine, T cell, B cell, and RIG-I receptor signaling pathways (Figure 5).

**Figure 6:**
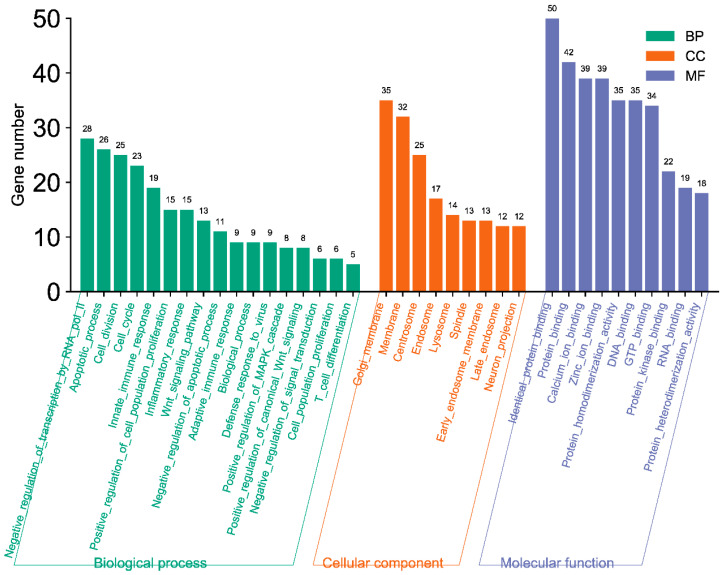
GO pathway enrichment analysis for some selected host cell miRNA candidates (miR-193a-3p, miR-494, miR-2375, and miR-12059). The number on the peak of each bar shows the gene numbers in specific pathways targeted and differently expressed by these miRNA candidates. The MiRWalk analysis and GO term BP, CC, and MF three-in-one bar plots were plotted by https://www.bioinformatics.com.cn/en, a free online platform for data analysis and visualization.

The GO enrichment analysis showed that bovine miR-193a-3p, miR-494, miR-2375, and miR-12059 target genes involved in pathways that negatively regulate different biological processes (Figure 6). These four bovine miRNA candidates potentially target some genes that regulate cell proliferation and inflammatory responses. Furthermore, the GO analysis also showed that many genes were targeted in the Golgi and cellular components’ whole membrane, which may influence the BCoV-S attachment to the cell membrane of the target cells (Figure 6).

### 3.5. Bovine Host Cell miRNAs Targeting Some Immune Suppressor Genes

**Figure 7:**
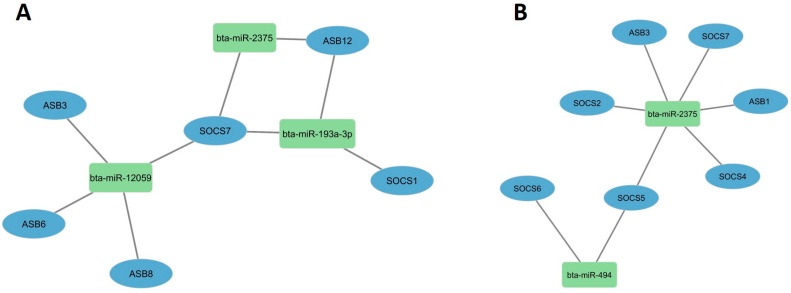
Host cell miRNAs potentially targeting some essential immune suppressor genes: (**A**) The immune-suppressor genes targeted by miR-193a-3p, miR-2375, and miR-12059 were predicted by miRWalk software; (**B**) Immune-suppressor genes targeted by miR-494 and miR-2375 were predicted via TargetScan8.0 software. The figure was developed using the Cytoscape software http://mirwalk.umm.uni-heidelberg.de/ (accessed on 20 March 2024).

The bovine miR-193a-3p, miR-494, miR-2375, and miR-12059 potentially target BCoV-S glycoprotein and regulate some bovine signal transduction pathways. We aimed to investigate if these miRNAs can regulate host immune suppression pathways. In silico prediction analysis showed that miR-2375 binds to SOCS7 and Ankyrin repeat and SOCS Box (ASB) containing 12 (ASB12) (Figure 7A), while bovine miR-12059 binds to SOCS7, ASB3, ASB6, and ASB8 (Figure 7A). miR-193a-3p binds to SOCS1, SOCS7, and ASB12 (Figure 7A), while miR-494 did not bind with any potential immune-suppressing genes. Interestingly, SOCS7 has recognition sites with miR-193a-3p, miR-2375, and miR-12059, while ASB12 binds to miR-193a-3p and miR-2375 (Figure 7A).

To support these findings, we expanded our search and used another online server, TargetScan8.0, to confirm our predictions. Results indicated that both miR-2375 and miR-494 only bind to some immune-suppressor genes. It is worth mentioning here that the bovine miR-12059 was not included in the TargetScsan8.0 database. The bovine miR-2375 binds to six immune-suppressing genes, particularly SOCS2, SOCS4, SOCS5, and SOCS7 (Figure 7B). Meanwhile, bovine miR-494 could only potentially target both SOCS5 and SOCS6 (Figure 7B).

### 3.6. BCoV Infection Induces Differential Display of the Host Cell miRNAs to Fine-Tune the Viral Tropism and Viral Replication

**Figure 8:**
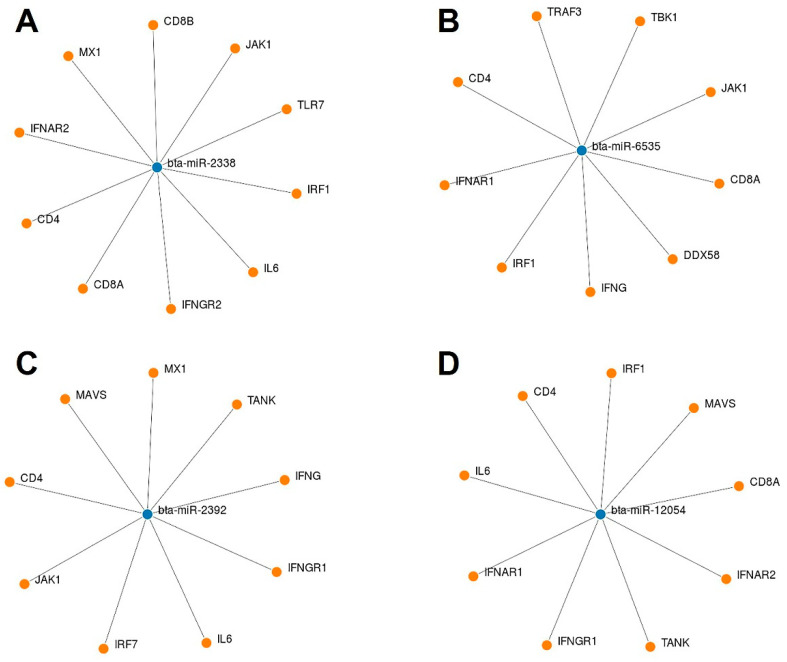
List of some selected bovine miRNAs and their potential targeting to some key host immune regulatory genes: (**A**) bta-miR-2338 targeted genes; (**B**) bta-miR-6535 targeted genes; (**C**) bta-miR-2392 targeted genes; and (**D**) bta-miR-12054 targeted genes. The miRNAs and gene binding predictions were performed by miRWalk software.

**Figure 9:**
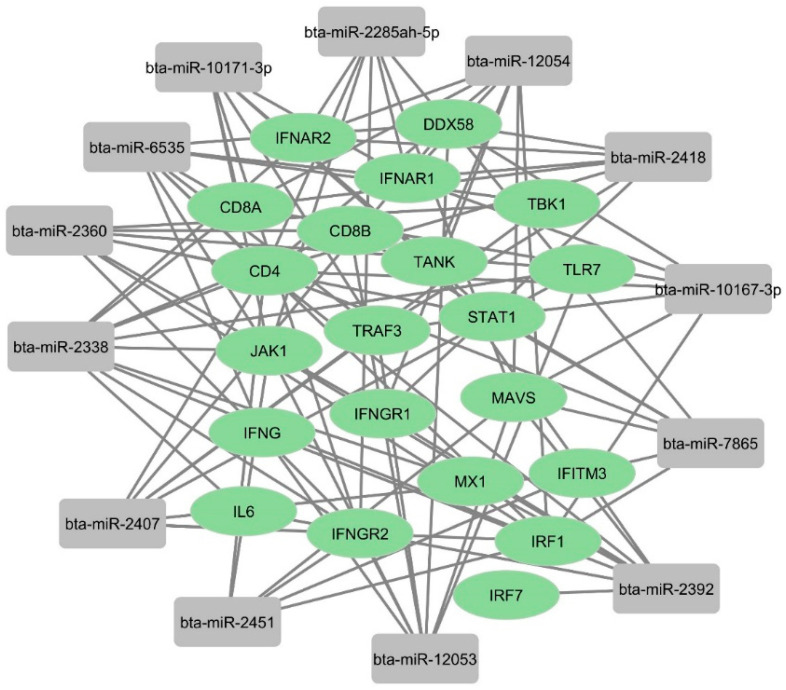
Network analysis of predicted bovine miRNAs targeting host immune regulatory genes: The binding of bovine miRNAs and their potential target genes was predicted by miRWalk. The figure showing network interaction was developed in Cytoscape.

We used an online tool to identify some potential bovine miRNAs targeting host immune regulatory genes (Supplementary File S2). Twelve miRNAs were selected among these candidates based on their putative involvement in various host immune response pathways. Our findings suggest that beta-miR-2338 had the maximum number of binding sites (10 sites) (Figure 8 and Figure 9). Additionally, three miRNAs (bta-miR-6535, bta-miR-2392, and bta-miR-12054) target nine host genes (Figure 8 and Figure 9). Furthermore, three miRNAs (bta-miR-12053, bta-miR-2360, bta-miR-2407) each can potentially target eight genes, and five miRNAs (bta-miR-10167-3p, bta-miR-10171-3p, bta-miR-2285ah-5p, bta-miR-2418, bta-miR-7865) target seven genes involved in host immune regulation (Figure 9). These results suggest that these miRNAs, particularly bta-miR-2338, bta-miR-6535, bta-miR-2392, and bta-miR-12054, could be potential miRNAs involved in BCoV immune evasion and disease progression.

**Figure 10:**
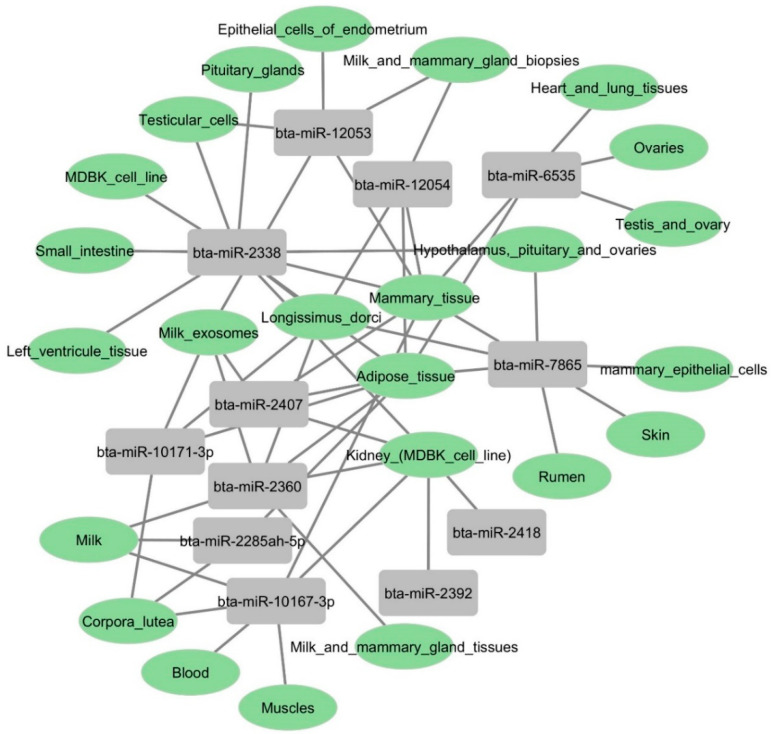
Gene atlas showing the expression profiles of some selected bovine miRNAs in various host tissues: Bovine candidates’ tissue origin was identified using the RumimiR database. The figure showing network interaction of miRNAs with bovine tissues was produced using Cytoscape.

To explore the tissue expression levels of these 12 miRNA candidates, we used an online prediction tool called RumimiR [56]. This database identifies miRNAs based on animal origin, tissue distribution, and experimental conditions. As a primary target tissue following BCoV infection, we focused on miRNAs abundant in the intestine and kidney. The in silico prediction results showed that bta-miR-2338 was enriched in small intestine and kidney tissues (Figure 10). Six other miRNAs (bta-miR-2407, bta-miR-2360, bta-miR-2285ah-5p, bta-miR-10167-3p, bta-miR-2392, and bta-miR-2418) were also highly expressed in bovine kidney cells (Figure 10). These results indicate that beta-miR-2338 could be a potential marker for tissue tropism and BCoV infection.

### 3.7. Bovine miRNAs Regulating Some Host Transcription Factors That Control the Expression of Some Key Host Genes

**Figure 11:**
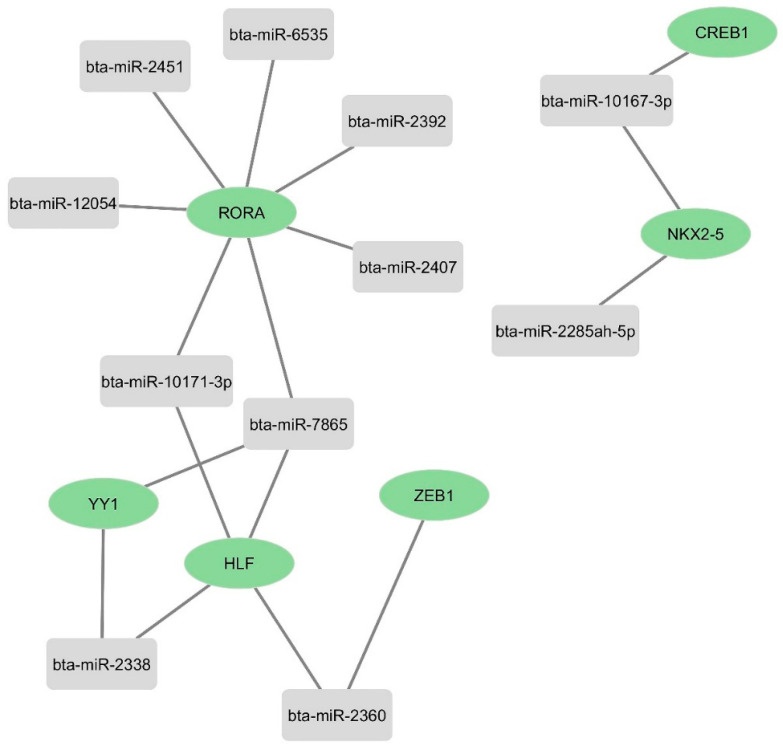
Network analysis of some selected bovine miRNAs targeting some important host transcription factors: The targeting of the selected bovine miRNAs and their target genes was predicted by miRWalk. The figure showing network interaction was produced using Cytoscape.

To understand the miRNA transcription factor (TF) co-regulation of host genes that might potentially influence BCoV replication, we generated a miRNAs–TFs network. We conducted the online target prediction of these 12 miRNAs with TFs already reported to have strong potential as diagnostic biomarkers for BCoV infection [59]. Our results showed that seven miRNAs (miR-10171-3p, miR-7865, miR-2407, miR-6535, miR-2392, miR-12054, and miR-2451) out of those 12 candidates target the Retinoic acid receptor-related orphan receptor alpha (RORα) transcription factor (Figure 11). RORα is highly expressed in different cells and tissues, including macrophages, suggesting its potential roles in the immune responses [60,61]. Furthermore, miR-2338 targets the Yin Yang 1 (YY1) transcription factor, which is highly expressed in mammalian tissues, and the oncogenic fusion transcription factor (TCF3-HLF) (Figure 11).

### 3.8. The Putative Regulation of Some Critical Host Signal Transduction Pathways by Bovine miRNAs

**Figure 12:**
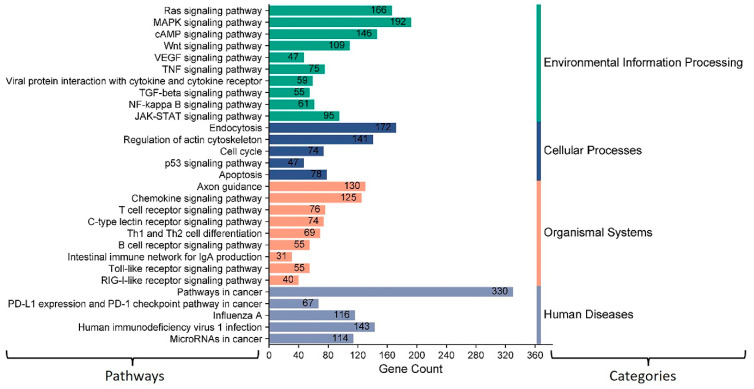
KEGG pathway enrichment analysis of some selected bovine miRNAs potentially targeting some immune regulatory genes. The number displayed on the peak of each bar shows the number of genes involved in specific pathways targeted by these miRNAs. The column on the left displays the name of each pathway. The column on the right side indicates KEGG pathways assigned by different categories. The MiRWalk analysis and pathway enrichment category plot were plotted using https://www.bioinformatics.com.cn/en, a free online platform for data analysis and visualization.

Our analysis showed 12 bovine miRNAs (miR-10167-3p, miR-10171-3p, miR-2285ah-5p, miR-2418, miR-7865, miR-12053, miR-2360, miR-2407, miR-6535, miR-2392, miR-12054, and miR-2338) potentially targeting the most significant immune regulatory genes (Figure 9). The KEGG gene enrichment analysis showed that these miRNAs mainly influence genes that regulate important host cellular signal transduction pathways. These include the Ras, MAPK, cAMP, Wnt, and JAK-STAT pathways (Figure 12). Furthermore, these miRNAs potentially target some genes involved in the host receptor signaling pathways, including chemokine, T cell, B cell, toll-like receptors (TLR), and RIG-I receptor signaling pathways (Figure 12). Most of these host cellular pathways directly or indirectly regulate host immune response.

**Figure 13:**
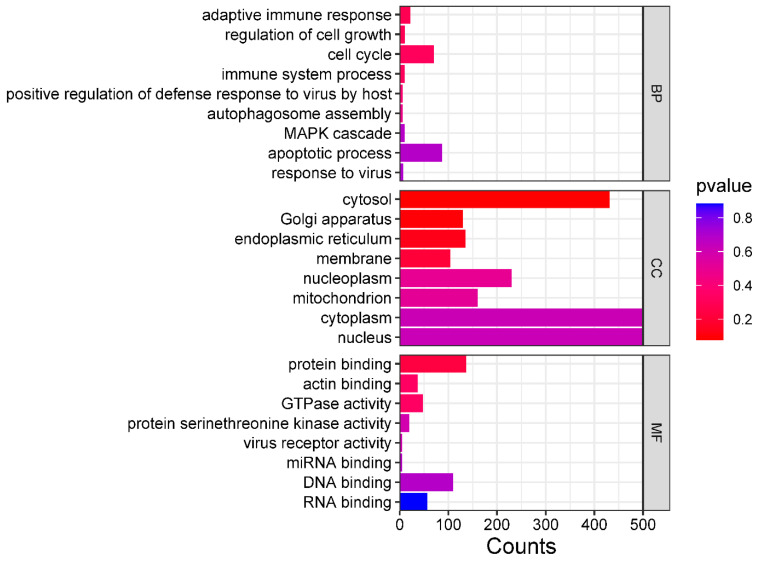
GO pathway enrichment analysis of bovine miRNAs targeting the immune regulatory genes. The counts represent the gene numbers (X-axis). The analysis was performed with MiRWalk, and GO term Biological Process (BP), Cellular Components (CC), and Molecular Function (MF) enriched horizontal bars with colors were plotted by https://www.bioinformatics.com.cn/en, a free online platform for data analysis and visualization.

The GO enrichment analysis showed that these bovine miRNAs target many genes in the host cell cytoplasm and nucleus (Figure 13). Moreover, these miRNAs also target genes involved in the cell cycle, protein binding, DNA binding, and RNA binding (Figure 13). These results indicated that these bovine miRNAs, especially miR-6535, miR-2392, miR-12054, and miR-2338, could potentially promote BCoV replication and fine-tune the viral tissue tropism and pathogenesis.

## 4. Discussion

BCoV causes several clinical syndromes in the affected animals and shows a dual tropism in the respiratory and digestive tracts. Both the enteric and respiratory isolates of BCoV show a high degree of similarity on the genomic level [62]. Meanwhile, due to their high degree of similarity, there is a lack of any genetic marker for these enteric and respiratory isolates of BCoV. Thus, there appeared to be other host-related factors that may fine-tune this dual tissue tropism. MicroRNAs were believed to regulate some host cell genes essential during many viral replications, including SARS-CoV-2 infection. Some host miRNAs were implicated in the diagnosis and prognosis of the outcomes of SARS-CoV-2-infected patients [34]. The main goals of the current study were to study the potential roles of the host cell miRNAs in BCoV replication, tissue tropism, and immune regulation. Searching for some genetic miRNA markers for BCoV infection in general and even distinguishing between the enteric and respiratory isolates of BCoV were important contributions of this study.

### 4.1. Host miRNA Targeting BCoV Spike Protein at Various Locations

As in most other coronaviruses, the spike protein of BCoV also plays an essential role in attachment to the host cells, molecular pathogenesis, and immune evasion. Recent studies reported that host miRNAs could influence viral replication and alter the host gene expression profiles during SARS-CoV-2 replication [63]. Furthermore, 67 human miRNAs have been reported to target and affect the normal function of SARS-CoV-2 spike protein [63,64].

The BCoV-S1 subunit consists of two independent domains, the N-terminal domain (NTD) and the C domain or Receptor-Binding Domain (RBD), which act as the viral receptor-binding domains [20]. The BCoV and human HCoV-OC43 S1-NTD domains bind to some sugar moieties and act as viral lectins [21,22]. Both the BCoV and HCoV-OC43 also express a hemagglutinin-esterase (HE) that serves as a receptor-destroying enzyme and promotes viral detachment from the sugar on infected cells, which is consistent with the presence of a viral lectin in their spike proteins [12]. Peng et al. reported four critical sugar-binding sites in the BCoV S1-NTD domain: Tyr-162, Glu-182, Trp-184, and His-185 [23]. However, the Furin-binding and sugar-binding sites serve as sensitive spike protein regions for virus invasion into the host. Thus, binding some host miRNAs to these motifs may prevent attachment and invasion of viral RNA into the host cell. We found that bta-miR-193a-3p binds to Tyr-162 and bta-miR-494 binds to the His-185 residue of the S1-NTD sugar-binding site. Furthermore, bta-miR-2375 binds to the S1/S2 Furin cleavage site, and bta-miR-12059 binds to the conserved S2 nonamer site.

### 4.2. Impacts of Host miRNA Targeting Various BCoV Genome Regions Related to the Virus’s Infectivity and Pathogenesis

Besides the roles of miRNAs in binding to the viral genome, miRNAs binding to the mRNA of one or more target genes of the host cells may alter the mRNA expression and that of its encoded protein at the post-transcriptional level. This alteration in the mRNA or protein levels may impact a single gene or an entire signal transduction pathway in the context of the BCoV infection. One of the effective methods for understanding the dual tissue tropism of BCoV infection and pathogenesis is to comprehend host cell-related molecular signal transduction pathways during viral infection. First, we found that miR-193a-3p, miR-494, miR-2375, and miR-12059 target BcoV-S protein and could influence the virus’s infectivity. Here, we wanted to explore the influence of these four miRNAs on host signal transduction pathways related to BcoV infection. Most of the cellular signaling and apoptosis pathways enriched by miR-193a-3p, miR-494, miR-2375, and miR-12059 have been recently reported as COVID-19-associated pathways [65,66]. Therefore, we conducted the KEGG and the GO pathway enrichment analyses for some selected miRNA candidates (miR-193a-3p, miR-494, miR-2375, and miR-12059). We found that KEGG pathways affected by bovine miR-193a-3p, miR-494, miR-2375, and miR-12059 are primarily enriched in Mitogen-Activated Protein Kinase (MAPK) and Phosphatidylinositol 3-Kinase PI3K/ AKT (PI3K-Akt) signaling pathways (Figure 5). Consistently, Tao et al. (2020) showed that SARS-CoV, like other respiratory viruses, hijacks MAPK-p38 activity and promotes viral replication [67].

The PI3K-Akt pathway has been linked to various aspects of virus entry into cells and the development of immune responses. It can significantly influence viral cell invasion, growth, migration, and proliferation. It can promote angiogenesis while inhibiting apoptosis. SARS-CoV-2 endocytosis occurs through a clathrin-mediated pathway modulated by PI3K/AKT signaling [65]. A recent study found that cancer pathways are the first disease pathways related to COVID-19. A total of 98 genes were commonly expressed in cancer and COVID-19 disease pathways. It was also shown that patients with the human influenza virus type-A infection pattern shared 57 common genes with SARS-CoV-2 infected patients [68]. Similarly, our study indicated that most human disease pathways, including the influenza virus type-A, are influenced by the four bovine miRNA candidates mentioned above (miR-193a-3p, miR-494, miR-2375, and miR-12059).

Another study showed that viral miRNAs could target host genes and establish favorable host cell conditions for virus replication [69]. In this context, several studies found viral miRNAs predicted to target and influence host immune regulatory pathways, such as T cell-mediated immunity, cytokine response, biological adhesion, and autophagy, as well as other regulatory signaling pathways like WNT, MAPK, and TGF-beta signaling [63,70,71,72,73]. In contrast, this study showed that DEGs mediated by bovine miR-193a-3p, miR-494, miR-2375, and miR-12059 were also enriched in host immune regulatory pathways, like T cell-mediated immunity, cytokine and chemokine, and apoptosis, as well as other regulatory signaling pathways like MAPK, and WNT in both KEGG and GO pathways.

The Suppressors Of Cytokine Signaling (SOCS) family are intracellular proteins that inhibit cytokine activation. SARS-CoV-2 suppresses the host immune response by activating SOCS1 or SOCS3 early in infection [44,45]. Furthermore, vesicular stomatitis virus-induced miR-155 targets SOCS1 and positively regulates the host’s innate immune response by enhancing type I IFN signaling through RIG-I/JNK/NF-kB-dependent mechanisms [74]. Another study suggested that SARS-CoV-2 induces miR-155, which targets SOCS1, affecting Th17/Treg in patients [46]. Other coronaviruses, such as the transmissible gastroenteritis virus (TGEV), downregulate miR-30a-5p expression, which promotes antiviral responses by targeting SOCS1 and SOCS3, enhancing TGEV replication [75]. Moreover, both miR-130b and miR-454 can inhibit Infectious Bursal Disease Virus (IBDV) replication by targeting host SOCS5 and SOCS6, respectively [76,77]. miR-145 targets SOCS7 and promotes interferon induction in bladder cancer cells [78] and Hepatitis C virus (HCV) [79]. In contrast, our study found that the bovine miR-193a-3p, miR-2375, and miR-12059 potentially bind to the host SOCS7 (Figure 7A,B), while SOCS1 only binds to miR-193a-3p. In addition, bovine miR-494 targets SOCS5 and SOCS6, while bovine miR-2375 targets SOCS2, SOCS4, SOCS5, and SOCS7 (Figure 7A,B), thus indicating that, on the one hand, bovine miRNAs miR-193a-3p, miR-494, miR-2375, and miR-12059 target the BCoV-S glycoprotein, while, on the other hand, they target host cytokine-suppressing genes, indicating that these miRNAs could indirectly enhance the host immune response and inhibit BCoV replication.

### 4.3. Impacts of Host miRNA Targeting Various Immune Regulatory Genes in the Context of BCoV Infection

Although the complete mechanism of miRNAs upon viral infection is not fully understood, it involves interactions with virus and host cells and modulates virus replication or host biological pathways. The virus most likely hijacks the host miRNAs to alter host genes, creates a suitable environment for their replication, and prevents the host’s antiviral immune response [80]. To identify the bovine miRNA candidates promoting BCoV pathogenesis and tissue tropism, we selected genes involved in the immune response against some coronaviruses, including BCoV. A recent study demonstrated that BCoV-Nucleocapsid protein alters type I interferon production by inhibiting MDA5, MAVS, TBK1, and IRF3 in the RLR pathway [81]. Another study used an artificial intelligence bioinformatic approach to prove that PI3K/AKT, MAPK, and TLRs are the three most significant pathways involved in COVID-19 infection [68]. At least eight proteins among the viral proteins expressed by SARS-CoV or SARS-CoV-2 have been proven to inhibit type I IFN response [82,83]. To understand the molecular mechanism and the involvement of some host miRNAs in regulating these pathways during BCoV replication, we analyzed complete bovine miRNAs with host immune regulatory pathways. Results showed that bta-miR-2338 targeted 10 critical genes involved in host immune response (Figure 8A). In addition, bta-miR-6535, bta-miR-2392, and bta-miR-12054 each could potentially target nine genes involved in host immune response (Figure 8B–D). Remarkably, all four of these miRNAs (bta-miR-2338, bta-miR-6535, bta-miR-2392, and bta-miR-12054) also target CD4 T helper cell receptors (Figure 9). The KEGG gene enrichment analysis performed for these miRNAs supported the above statement and shared the interest of these miRNAs in influencing receptor binding pathways of T cell, B cell, toll-like receptors (TLR), and RIG-I receptor signaling pathways (Figure 12). All of this evidence strongly suggests that bta-miR-2338, bta-miR-6535, bta-miR-2392, and bta-miR-12054 could potentially enhance the propagation/replication of BCoV.

Despite their role in viral propagation and host immune response activation, miRNAs can also mediate tissue tropism upon virus infection. In rats, the pseudorabies virus (PRV) infection showed several host miRNAs differently expressed in the lungs and spleen, regulating the respiratory and immune system and promoting tissue tropism [37]. In this study, the GO functional annotation showed that these bovine miRNAs (bta-miR-2338, bta-miR-6535, bta-miR-2392, and bta-miR-12054) primarily targeted the host cell cycle, protein binding, DNA binding, RNA binding, cytosol, cytoplasm, and nucleus (Figure 13). Furthermore, an online prediction tool also indicated the high level of expression of bta-miR-2338 in different bovine tissues, including the small intestine, heart tissues, and kidney cells (Figure 10). These observations imply that the over-expression of bta-miR-2338 upon BCoV infection can positively regulate viral pathogenesis and tissue tropism in the host intestine and kidney.

### 4.4. Potential Role of miRNAs as Biological Markers for BCoV Infection

Several in silico studies have been conducted to predict host miRNA interactions with different viruses, particularly in the case of SARS-CoV-2 infection in humans [84,85]. Although miRNAs only target individual genes, advanced research has demonstrated that miRNAs can modulate the complete signal transduction pathways. Understanding the full range of miRNA functions and their roles in the pathogenesis and control of some diseases has sparked widespread interest in their use to regulate immune pathways, offering a promising therapeutic option for many emerging diseases. The current study aimed to identify some potential bovine miRNAs that can be used as diagnostic, therapeutic, and genetic biomarkers for BCoV infection in cattle.

This study elucidates the significance of bovine miR-193a-3p, miR-494, miR-2375, and miR-12059 as potential therapeutic agents against BCoV infection. Our findings demonstrate that these miRNAs have potential roles in fine-tuning BCoV replication. On the one hand, miR-193a-3p, miR-494, miR-2375, and miR-12059 target viral genomes and inhibit their replication. On the other hand, they inhibit the host immune suppressor pathway. Bovine miR-494 shares a homologous seed region with human miR-494-3p. A recent study proposed that has-miR-494-3p was significantly altered in studied COVID-19 patients [36]. In addition, human miR-494 has been reported as a novel therapeutic marker for human breast cancer [86,87]. These findings suggest that bovine miR-494 could be a potential therapeutic marker against BCoV.

Furthermore, by using different bioinformatic prediction tools, we believe four bovine miRNAs (bta-miR-2338, bta-miR-6535, bta-miR-2392, and bta-miR-12054) could influence host immune mechanisms by targeting several immune regulatory genes and transcription factors. In contrast, McDonald et al. [35] found that miR-2392 was detected in the blood and urine of COVID-19-positive patients but was absent in COVID-19-naive patients. Furthermore, they designed miRNA-based antiviral therapeutics that target miR-2392 and significantly reduced SARS-CoV-2 in hamsters and may inhibit COVID-19 in humans [35]. Altogether, these findings highlight the potential of miR-2392 as a biomarker for the diagnosis or prognosis of both BCoV and COVID-19.

miRNA expression has been reported to change during the progression of crucial infectious diseases like bovine viral diarrhea (BVD) and foot and mouth disease (FMD). Evidence showed that miR-423-5p and miR-151-3p exhibit differential expression patterns across different time points post-BVD infection, indicating their potential significance in BVD infection [88]. Another study showed that miR-17-5p, miR-31, and miR-1281 are potential biomarkers for foot and mouth diseases virus (FMD), offering insights into both acute infection and viral persistence [89]. Apart from viral infection, several studies showed the importance of miRNAs as biomarkers in bacterial infections affecting bovines. Wang et al. [90] showed that miR-199a plays an important role in Mycobacterium bovis infection by downregulating host IFN-B expression. Similarly, Iannaccone et al. [91] identified miR-146a as a prognostic biomarker for bovine tuberculosis infection.

In conclusion, host cell miRNAs may act as critical mediators in the differential tropism of BCoV infection (Ent/Resp). These miRNAs represent a promising trend, especially as diagnostic and genetic markers for BCoV. They may also pave the way for developing novel miRNA-based vaccines against BCoV in the future. Further studies are needed to explore the roles of miRNAs in the molecular pathogenesis and immune response/evasion of BCoV.

## 5. Limitations of the Current Study

The main limitations of the current study were (1) a lack of data on bovine miRNAs in the public domain, (2) the limited number of studies on the roles of miRNAs in the field of BCoV, and (3) the limited number of online tools and algorithms that can help in predicting the functions of bovine miRNAs and their target genes.

## Figures and Tables

**Table 1:** List of BCoV genome isolates and their demographic data retrieved from GenBank.

Accession No.	Strain/Isolate	Country	Year
Enteric			
U00735	Mebus	USA	1972
AB354579	Kakegawa	Japan	1976
AF391541	BCoV-ENT	USA	1998
DQ915164	Alpaca	USA	1998
EF424615	Bovine coronavirus E-AH65	USA	2007
EF424619	Bovine coronavirus E-AH187	USA	2007
KX982264	BCoV_2014_13	France	2014
KU886219	BCV-AKS-1	China	2015
MG518518	Water deer coronavirus isolate W17-18	Korea	2018
LC642814	strain GF2020	Japan	2021
Respiratory			
FJ938066	Bovine/US/OH-440-TC/1996	USA	1996
AF391542	BCoV-LUN	USA	1998
EF424617	Bovine coronavirus R-AH65	USA	2007
EF424620	Bovine coronavirus R-AH187	USA	2007
MG757140	BCoV/France/11-ICSA16-LBA/2014	France	2014
MG757138	BCoV/France/ICSA21L3/LBA/2014	France	2014
KX432213	Canine respiratory coronavirus strain BJ232	China	2016
ON146444	Bovine coronavirus strain MARC/BRCV_2014	USA	2022

**Table 2:** Mapping the bovine miRNAs targeting some critical domains in the BCoV-S glycoprotein.

Domain	Location (bp)	Sequence (aa)	Sequence (bp)	Gene
S1-NTD Sugar Binding Sites	24124–24198	YTMCEYPHTICHPNLGNKRVELWHW	UAUACUAUGUGCGAGUACCCACAUACGAUUUGUCAUCCUAAUCUGGGUAAUAAACGCGUAGAACUAUGGCAUUGG	S1
S1/S2 Furin Cleavage Site	25930–25944	RRSRR	AGACGAAGUCGUAGA	S1/S2
GxCx Motif	25903–25914	GYCV	GGUUACUGUGUG	S1
Conserved S2 Nonamers	26044–26070	IPSEFTIGN	AUACCUUCAGAGUUUACUAUAGGUAAU	S2

The amino acids and the nucleotides are highlighted in red font, showing the binding sites of the sugar molecules.

**Table 3:** Hybridization of some host cell miRNAs potentially targeting some critical domains of BCoV-S glycoprotein.

miRNAs	Left-Most Position of Target Site	Binding Sites	Folding Energy (in Kcal/mol)	Heteroduplex
bta-miR-193a-3p	24103	S1-NTD Sugar Binding Sites Tyr-162 Residue	−12.80	TTAGAGATCTCTGTTTGCCAGTA || || ||| |||||| TGACCCT-GAAACATC CGGTCA A
bta-miR-494	24196	S1-NTD Sugar Binding Sites His-185 Residue	−13.30	TGGGATACAGGTGT-TGTTTCC |||| |||||| CTCCAAAGGGCACAT ACAAAG T
bta-miR-2375	25938	S1/S2 Furin Cleavage Site	−13.30	TCGTAGAGCGATT-ACCACTGGT :||| | |: |||||| CTAGTCTGGAGAGGTA GTGACC C
bta-miR-12059	26061	Conserved S2 Nonamers	−16.30	TATAGGTA-ATATGGAGGAGT |||:| ||||||| TCCTCCGTCCCAC CCTCCTC T

The highlighted nucleotide sequences in red color indicate the hybridization between the seed region of selected miRNA and the target region in BCoV-S glycoprotein.

## Data Availability

Data are contained within the article and the Supplementary Materials.

## Supplementary Materials

The following supporting information can be downloaded at https://www.mdpi.com/article/10.3390/microorganisms12050897/s1, Supplementary File S1. List of host miRNA prediction; Supplementary File S2. Bovine miRNA repository.

## References

[1] Clark M. (1993). Bovine coronavirus. Br. Vet. J..

[2] Saif L.J. (2010). Bovine respiratory coronavirus. Vet. Clin. Food Anim. Pract..

[3] Kiser J.N., Neibergs H.L. (2021). Identifying Loci Associated with Bovine Corona virus Infection and Bovine Respiratory Disease in Dairy and Feedlot Cattle. Front. Vet. Sci..

[4] Rahe M.C., Magstadt D.R., Groeltz-Thrush J., Gauger P.C., Zhang J., Schwartz K.J., Siepker C.L. (2022). Bovine coronavirus in the lower respiratory tract of cattle with respiratory disease. J. Vet. Diagn. Investig..

[5] Vlasova A.N., Saif L.J. (2021). Bovine Coronavirus and the Associated Diseases. Front. Vet. Sci..

[6] Castells M., Victoria M., Colina R., Musto H., Cristina J. (2017). Genome-wide analysis of codon usage bias in Bovine Coronavirus. Virol. J..

[7] Hodnik J.J., Jezek J., Staric J. (2020). Coronaviruses in cattle. Trop. Anim. Health Prod..

[8] Lathrop S.L., Wittum T.E., Brock K.V., Loerch S.C., Perino L.J., Bingham H.R., McCollum F.T., Saif L.J. (2000). Association between infection of the respiratory tract attributable to bovine coronavirus and health and growth performance of cattle in feedlots. Am. J. Vet. Res..

[9] Saif L.J., Jung K. (2020). Comparative pathogenesis of bovine and porcine respiratory coronaviruses in the animal host species and SARS-CoV-2 in humans. J. Clin. Microbiol..

[10] Salem E., Dhanasekaran V., Cassard H., Hause B., Maman S., Meyer G., Ducatez M.F. (2020). Global Transmission, Spatial Segregation, and Recombination Determine the Long-Term Evolution and Epidemiology of Bovine Coronaviruses. Viruses.

[11] Ven S., Arunvipas P., Lertwatcharasarakul P., Ratanapob N. (2021). Seroprevalence of bovine coronavirus and factors associated with the serological status in dairy cattle in the western region of Thailand. Vet. World.

[12] Zeng Q., Langereis M.A., Van Vliet A.L., Huizinga E.G., de Groot R.J. (2008). Structure of coronavirus hemagglutinin-esterase offers insight into corona and influenza virus evolution. Proc. Natl. Acad. Sci. USA.

[13] Zhang M., Hill J.E., Fernando C., Alexander T.W., Timsit E., van der Meer F., Huang Y. (2019). Respiratory viruses identified in western Canadian beef cattle by metagenomic sequencing and their association with bovine respiratory disease. Transbound. Emerg. Dis..

[14] Cox G.J., Parker M.D., Babiuk L.A. (1991). Bovine coronavirus nonstructural protein ns2 is a phosphoprotein. Virology.

[15] Gustin K.M., Guan B.-J., Dziduszko A., Brian D.A. (2009). Bovine coronavirus nonstructural protein 1 (p28) is an RNA binding protein that binds terminal genomic cis-replication elements. J. Virol..

[16] Trbojević-Akmačić I., Petrović T., Lauc G. (2021). SARS-CoV-2 S glycoprotein binding to multiple host receptors enables cell entry and infection. Glycoconj. J..

[17] Li F. (2016). Structure, function, and evolution of coronavirus spike proteins. Annu. Rev. Virol..

[18] Belouzard S., Millet J.K., Licitra B.N., Whittaker G.R. (2012). Mechanisms of coronavirus cell entry mediated by the viral spike protein. Viruses.

[19] Lang Y., Li W., Li Z., Koerhuis D., van den Burg A.C.S., Rozemuller E., Bosch B.J., van Kuppeveld F.J.M., Boons G.J., Huizinga E.G. (2020). Coronavirus hemagglutinin-esterase and spike proteins coevolve for functional balance and optimal virion avidity. Proc. Natl. Acad. Sci. USA.

[20] Li F. (2012). Evidence for a common evolutionary origin of coronavirus spike protein receptor-binding subunits. J. Virol..

[21] Künkel F., Herrler G. (1993). Structural and functional analysis of the surface protein of human coronavirus OC43. Virology.

[22] Schultze B., Gross H., Brossmer R., Herrler G. (1991). The S protein of bovine coronavirus is a hemagglutinin recognizing 9-O-acetylated sialic acid as a receptor determinant. J. Virol..

[23] Peng G., Xu L., Lin Y.-L., Chen L., Pasquarella J.R., Holmes K.V., Li F. (2012). Crystal structure of bovine coronavirus spike protein lectin domain. J. Biol. Chem..

[24] Tang T., Jaimes J.A., Bidon M.K., Straus M.R., Daniel S., Whittaker G.R. (2021). Proteolytic Activation of SARS-CoV-2 Spike at the S1/S2 Boundary: Potential Role of Proteases beyond Furin. ACS Infect. Dis..

[25] Baranowski E., Ruiz-Jarabo C.M., Domingo E. (2001). Evolution of cell recognition by viruses. Science.

[26] Cheng Y.W., Chao T.L., Li C.L., Chiu M.F., Kao H.C., Wang S.H., Pang Y.H., Lin C.H., Tsai Y.M., Lee W.H. (2020). Furin Inhibitors Block SARS-CoV-2 Spike Protein Cleavage to Suppress Virus Production and Cytopathic Effects. Cell Rep..

[27] Bartel D.P. (2004). MicroRNAs: Genomics, biogenesis, mechanism, and function. Cell.

[28] Lewis B.P., Burge C.B., Bartel D.P. (2005). Conserved seed pairing, often flanked by adenosines, indicates that thousands of human genes are microRNA targets. Cell.

[29] Lecellier C.-H., Dunoyer P., Arar K., Lehmann-Che J., Eyquem S., Himber C., Saïb A., Voinnet O. (2005). A cellular microRNA mediates antiviral defense in human cells. Science.

[30] Triboulet R., Mari B., Lin Y.-L., Chable-Bessia C., Bennasser Y., Lebrigand K., Cardinaud B., Maurin T., Barbry P., Baillat V. (2007). Suppression of microRNA-silencing pathway by HIV-1 during virus replication. Science.

[31] Zhuo Y., Gao G., Shi J., Zhou X., Wang X. (2013). miRNAs: Biogenesis, origin and evolution, functions on virus-host interaction. Cell. Physiol. Biochem..

[32] Abu-Izneid T., AlHajri N., Ibrahim A.M., Javed M.N., Salem K.M., Pottoo F.H., Kamal M.A. (2021). Micro-RNAs in the regulation of immune response against SARS CoV-2 and other viral infections. J. Adv. Res..

[33] Ingle H., Kumar S., Raut A.A., Mishra A., Kulkarni D.D., Kameyama T., Takaoka A., Akira S., Kumar H. (2015). The microRNA miR-485 targets host and influenza virus transcripts to regulate antiviral immunity and restrict viral replication. Sci. Signal.

[34] Chow J.T., Salmena L. (2020). Prediction and Analysis of SARS-CoV-2-Targeting MicroRNA in Human Lung Epithelium. Genes.

[35] McDonald J.T., Enguita F.J., Taylor D., Griffin R.J., Priebe W., Emmett M.R., Sajadi M.M., Harris A.D., Clement J., Dybas J.M. (2021). Role of miR-2392 in driving SARS-CoV-2 infection. Cell Rep..

[36] Akula S.M., Bolin P., Cook P.P. (2022). Cellular miR-150-5p may have a crucial role to play in the biology of SARS-CoV-2 infection by regulating nsp10 gene. RNA Biol..

[37] Fan Y., Zhu L., Sun X., Lyu W., Xu L., Yin Y., Zhao J., Huang J., Den Y., Jiang Z. (2019). Exploring the tissue tropism of pseudorabies virus based on miRNA level analysis. BMC Microbiol..

[38] Chetta M., Rosati A., Marzullo L., Tarsitano M., Bukvic N. (2020). A SARS-CoV-2 host infection model network based on genomic human Transcription Factors (TFs) depletion. Heliyon.

[39] Gilbertson S., Federspiel J.D., Hartenian E., Cristea I.M., Glaunsinger B. (2018). Changes in mRNA abundance drive shuttling of RNA binding proteins, linking cytoplasmic RNA degradation to transcription. Elife.

[40] Tong Z., Cui Q., Wang J., Zhou Y. (2019). TransmiR v2.0: An updated transcription factor-microRNA regulation database. Nucleic Acids Res..

[41] Sardar R., Satish D., Gupta D. (2020). Identification of Novel SARS-CoV-2 Drug Targets by Host MicroRNAs and Transcription Factors Co-regulatory Interaction Network Analysis. Front. Genet..

[42] Yoshimura A., Naka T., Kubo M. (2007). SOCS proteins, cytokine signalling and immune regulation. Nat. Rev. Immunol..

[43] Linossi E.M., Calleja D.J., Nicholson S.E. (2018). Understanding SOCS protein specificity. Growth Factors.

[44] Ahmed C.M., Grams T.R., Bloom D.C., Johnson H.M., Lewin A.S. (2022). Individual and Synergistic Anti-Coronavirus Activities of SOCS1/3 Antagonist and Interferon α1 Peptides. Front. Immunol..

[45] Johnson H.M., Lewin A.S., Ahmed C.M. (2020). SOCS, intrinsic virulence factors, and treatment of COVID-19. Front. Immunol..

[46] Soltani-Zangbar M.S., Hajivalili M., Daneshdoust D., Ghadir S., Savari G., Zolfaghari M., Aghebati-Maleki L., Oloufi S., Nouri N., Amini N. (2023). SARS-CoV2 infection induce miR-155 expression and skewed Th17/Treg balance by changing SOCS1 level: A clinical study. Cytokine.

[47] Wu J., Chen Z.J. (2014). Innate immune sensing and signaling of cytosolic nucleic acids. Annu. Rev. Immunol..

[48] Fang R., Jiang Q., Zhou X., Wang C., Guan Y., Tao J., Xi J., Feng J.-M., Jiang Z. (2017). MAVS activates TBK1 and IKKε through TRAFs in NEMO dependent and independent manner. PLoS Pathog..

[49] Liu S., Cai X., Wu J., Cong Q., Chen X., Li T., Du F., Ren J., Wu Y.-T., Grishin N.V. (2015). Phosphorylation of innate immune adaptor proteins MAVS, STING, and TRIF induces IRF3 activation. Science.

[50] Oshiumi H. (2020). Recent advances and contradictions in the study of the individual roles of ubiquitin ligases that regulate RIG-I-like receptor-mediated antiviral innate immune responses. Front. Immunol..

[51] Zielecki F., Weber M., Eickmann M., Spiegelberg L., Zaki A.M., Matrosovich M., Becker S., Weber F. (2013). Human cell tropism and innate immune system interactions of human respiratory coronavirus EMC compared to those of severe acute respiratory syndrome coronavirus. J. Virol..

[52] Liu B., Zhang M., Chu H., Zhang H., Wu H., Song G., Wang P., Zhao K., Hou J., Wang X. (2017). The ubiquitin E3 ligase TRIM31 promotes aggregation and activation of the signaling adaptor MAVS through Lys63-linked polyubiquitination. Nat. Immunol..

[53] Kozomara A., Birgaoanu M., Griffiths-Jones S. (2019). miRBase: From microRNA sequences to function. Nucleic Acids Res..

[54] Sticht C., De La Torre C., Parveen A., Gretz N. (2018). miRWalk: An online resource for prediction of microRNA binding sites. PLoS ONE.

[55] McGeary S.E., Lin K.S., Shi C.Y., Pham T.M., Bisaria N., Kelley G.M., Bartel D.P. (2019). The biochemical basis of microRNA targeting efficacy. Science.

[56] Bourdon C., Bardou P., Aujean E., Le Guillou S., Tosser-Klopp G., Le Provost F. (2019). RumimiR: A detailed microRNA database focused on ruminant species. Database.

[57] Shannon P., Markiel A., Ozier O., Baliga N.S., Wang J.T., Ramage D., Amin N., Schwikowski B., Ideker T. (2003). Cytoscape: A software environment for integrated models of biomolecular interaction networks. Genome Res..

[58] McNulty M.S., Bryson D.G., Allan G.M., Logan E.F. (1984). Coronavirus infection of the bovine respiratory tract. Vet. Microbiol..

[59] Morenikeji O.B., Strutton E., Wallace M., Bernard K., Yip E., Thomas B.N. (2020). Dissecting Transcription Factor-Target Interaction in Bovine Coronavirus Infection. Microorganisms.

[60] Garcia J.A., Volt H., Venegas C., Doerrier C., Escames G., Lopez L.C., Acuna-Castroviejo D. (2015). Disruption of the NF-kappaB/NLRP3 connection by melatonin requires retinoid-related orphan receptor-alpha and blocks the septic response in mice. FASEB J..

[61] Liu Y., Chen Y., Zhang J., Liu Y., Zhang Y., Su Z. (2017). Retinoic acid receptor-related orphan receptor alpha stimulates adipose tissue inflammation by modulating endoplasmic reticulum stress. J. Biol. Chem..

[62] Zhang X., Hasoksuz M., Spiro D., Halpin R., Wang S., Vlasova A., Janies D., Jones L.R., Ghedin E., Saif L.J. (2007). Quasispecies of bovine enteric and respiratory coronaviruses based on complete genome sequences and genetic changes after tissue culture adaptation. Virology.

[63] Demirci M.D.S., Adan A. (2020). Computational analysis of microRNA-mediated interactions in SARS-CoV-2 infection. PeerJ.

[64] Khokhar M., Tomo S., Purohit P. (2022). MicroRNAs based regulation of cytokine regulating immune expressed genes and their transcription factors in COVID-19. Meta Gene.

[65] Khezri M.R. (2021). PI3K/AKT signaling pathway: A possible target for adjuvant therapy in COVID-19. Hum. Cell.

[66] Khanmohammadi S., Rezaei N. (2021). Role of Toll-like receptors in the pathogenesis of COVID-19. J. Med. Virol..

[67] Tao Q., Du J., Li X., Zeng J., Tan B., Xu J., Lin W., Chen X.-l. (2020). Network pharmacology and molecular docking analysis on molecular targets and mechanisms of Huashi Baidu formula in the treatment of COVID-19. Drug Dev. Ind. Pharm..

[68] Taheri G., Habibi M. (2022). Comprehensive analysis of pathways in Coronavirus 2019 (COVID-19) using an unsupervised machine learning method. Appl. Soft Comput..

[69] Barbu M.G., Condrat C.E., Thompson D.C., Bugnar O.L., Cretoiu D., Toader O.D., Suciu N., Voinea S.C. (2020). MicroRNA involvement in signaling pathways during viral infection. Front. Cell Dev. Biol..

[70] Demongeot J., Seligmann H. (2020). SARS-CoV-2 and miRNA-like inhibition power. Med. Hypotheses.

[71] Khan M.A.-A.-K., Sany M.R.U., Islam M.S., Islam A.B.M.M.K. (2020). Epigenetic regulator miRNA pattern differences among SARS-CoV, SARS-CoV-2, and SARS-CoV-2 worldwide isolates delineated the mystery behind the epic pathogenicity and distinct clinical characteristics of pandemic COVID-19. Front. Genet..

[72] Saini S., Saini A., Thakur C.J., Kumar V., Gupta R.D., Sharma J.K. (2020). Genome-wide computational prediction of miRNAs in severe acute respiratory syndrome coronavirus 2 (SARS-CoV-2) revealed target genes involved in pulmonary vasculature and antiviral innate immunity. Mol. Biol. Res. Commun..

[73] Sarma A., Phukan H., Halder N., Madanan M.G. (2020). An in-silico approach to study the possible interactions of miRNA between human and SARS-CoV2. Comput. Biol. Chem..

[74] Wang P., Hou J., Lin L., Wang C., Liu X., Li D., Ma F., Wang Z., Cao X. (2010). Inducible microRNA-155 feedback promotes type I IFN signaling in antiviral innate immunity by targeting suppressor of cytokine signaling 1. J. Immunol..

[75] Ma Y., Wang C., Xue M., Fu F., Zhang X., Li L., Yin L., Xu W., Feng L., Liu P. (2018). The coronavirus transmissible gastroenteritis virus evades the type I interferon response through IRE1α-mediated manipulation of the microRNA miR-30a-5p/SOCS1/3 axis. J. Virol..

[76] Fu M., Wang B., Chen X., He Z., Wang Y., Li X., Cao H., Zheng S.J. (2018). MicroRNA gga-miR-130b suppresses infectious bursal disease virus replication via targeting of the viral genome and cellular suppressors of cytokine signaling 5. J. Virol..

[77] Fu M., Wang B., Chen X., He Z., Wang Y., Li X., Cao H., Zheng S.J. (2018). gga-miR-454 suppresses infectious bursal disease virus (IBDV) replication via directly targeting IBDV genomic segment B and cellular Suppressors of Cytokine Signaling 6 (SOCS6). Virus Res..

[78] Noguchi S., Yamada N., Kumazaki M., Yasui Y., Iwasaki J., Naito S., Akao Y. (2013). socs7, a target gene of microRNA-145, regulates interferon-β induction through STAT3 nuclear translocation in bladder cancer cells. Cell Death Dis..

[79] Zeng H., Li L., Gao Y., Wu G., Hou Z., Liu S. (2021). Long noncoding RNA UCA1 regulates HCV replication and antiviral response via miR-145-5p/SOCS7/IFN pathway. Int. J. Biol. Sci..

[80] Piedade D., Azevedo-Pereira J.M. (2016). The role of microRNAs in the pathogenesis of herpesvirus infection. Viruses.

[81] Xiangbo Z., Zhaofang Y., Jinjing G., Zhuandi G., Suocheng W. (2022). Bovine coronavirus nucleocapsid suppresses IFN-β production by inhibiting RIG-I-like receptors pathway in host cells. Arch. Microbiol..

[82] Totura A.L., Baric R.S. (2012). SARS coronavirus pathogenesis: Host innate immune responses and viral antagonism of interferon. Curr. Opin. Virol..

[83] Lei X., Dong X., Ma R., Wang W., Xiao X., Tian Z., Wang C., Wang Y., Li L., Ren L. (2020). Activation and evasion of type I interferon responses by SARS-CoV-2. Nat. Commun..

[84] Fulzele S., Sahay B., Yusufu I., Lee T.J., Sharma A., Kolhe R., Isales C.M. (2020). COVID-19 virulence in aged patients might be impacted by the host cellular microRNAs abundance/profile. Aging Dis..

[85] Nersisyan S., Engibaryan N., Gorbonos A., Kirdey K., Makhonin A., Tonevitsky A. (2020). Potential role of cellular miRNAs in coronavirus-host interplay. PeerJ.

[86] Ghorbanhosseini S.S., Nourbakhsh M., Zangooei M., Abdolvahabi Z., Bolandghamtpour Z., Hesari Z., Yousefi Z., Panahi G., Meshkani R. (2019). MicroRNA-494 induces breast cancer cell apoptosis and reduces cell viability by inhibition of nicotinamide phosphoribosyltransferase expression and activity. EXCLI J..

[87] Zhan M.N., Yu X.T., Tang J., Zhou C.X., Wang C.L., Yin Q.Q., Gong X.F., He M., He J.R., Chen G.Q. (2017). MicroRNA-494 inhibits breast cancer progression by directly targeting PAK1. Cell Death Dis..

[88] Taxis T.M., Bauermann F.V., Ridpath J.F., Casas E. (2017). Circulating MicroRNAs in Serum from Cattle Challenged with Bovine Viral Diarrhea Virus. Front. Genet..

[89] Stenfeldt C., Arzt J., Smoliga G., LaRocco M., Gutkoska J., Lawrence P. (2017). Proof-of-concept study: Profile of circulating microRNAs in Bovine serum harvested during acute and persistent FMDV infection. Virol. J..

[90] Wang J., Hussain T., Yue R., Liao Y., Li Q., Yao J., Song Y., Sun X., Wang N., Xu L. (2018). MicroRNA-199a Inhibits Cellular Autophagy and Downregulates IFN-beta Expression by Targeting TBK1 in Mycobacterium bovis Infected Cells. Front. Cell Infect. Microbiol..

[91] Iannaccone M., Cosenza G., Pauciullo A., Garofalo F., Proroga Y.T., Capuano F., Capparelli R. (2018). Milk microRNA-146a as a potential biomarker in bovine tuberculosis. J. Dairy Res..

