# Supplementary material for: The Potential Roles of Host Cell miRNAs in Fine-Tuning Bovine Coronavirus (BCoV) Molecular Pathogenesis, Tissue Tropism, and Immune Regulation
Source: Microorganisms. 2024 Apr 30;12(5):897. doi: 10.3390/microorganisms12050897 (PMC11124416; doi:10.3390/microorganisms12050897)
Supplement: Supplementary file 1 [file microorganisms-12-00897-s001.zip › Supplementary File S1. List of host miRNA prediction.pdf]

<table>
<tr><th>1. miR Name</th><th>leftmost position of predicted target site</th><th>folding energy (in -Kcal/mol)</th><th>heteroduplex</th><th>p value</th></tr>
<tr><td>2. bta-miR-101 MIMAT0003520</td><td>1909</td><td>-10.20</td><td>TTTAGTTTAGAAACTTTTACTGTT
||:|||| || ||||||
AAGTCAA--TAGTG-TCATGACAT</td><td>3.9E-1</td></tr>
<tr><td>3. bta-miR-21-3p MIMAT0003745</td><td>2021</td><td>-15.10</td><td>ACGCAGGTAAAATCTGCCATGCTGTT
  :||| : ||| :| |||||||
TCTGTCGG--GTAGCTG--ACGACAA</td><td>3.57E-1</td></tr>
<tr><td>4. bta-miR-410 MIMAT0009311</td><td>2243</td><td>-6.10</td><td>TTTTGC--ATGGTGCTTATATA
    || ||| ||||||
TGTCCGGTAGACAC-AATATAA</td><td>2.61E-1</td></tr>
<tr><td>5. bta-miR-2396 MIMAT0011949</td><td>2864</td><td>-10.00</td><td>CTATT--TTAAACACTGCATGT
|:||: || ||||||
GGTAGTTCTGGGGTCACGTACT</td><td>1.36E-1</td></tr>
<tr><td>6. bta-miR-410 MIMAT0009311</td><td>3495</td><td>-16.30</td><td>GCAGGATCTTTGGGTGTTATATA
:|||| :| |: |||||||||
TGTCC-GGTAG-ACACAATATAA</td><td>2.58E-1</td></tr>
<tr><td>7. bta-miR-2285dc MIMAT0046710</td><td>5796</td><td>-9.50</td><td>TAAAAATTTGAAACAAACTTTTA
 |||||::|:|| |||||||
CTTTTTGGATTT-GATTGAAAAA</td><td>2.39E-2</td></tr>
<tr><td>8. bta-miR-2285cr MIMAT0046691</td><td>5797</td><td>-9.90</td><td>AAAAATTTGAAACAAACTTTT
  |||::|||| |||||||
TCTTTGGACTT-GCTTGAAAA</td><td>2.39E-2</td></tr>
<tr><td>9. bta-miR-503-3p MIMAT0025558</td><td>5897</td><td>-11.90</td><td>ACGG-AGGTAAGTATTATACTCA
 ||| :|:| |: ||||||
GGCCCGTCGTCTTTGTTATGAGG</td><td>6.48E-2</td></tr>
<tr><td>10. bta-miR-2406 MIMAT0011964</td><td>6171</td><td>-13.30</td><td>AAGCCATG-AGCAAGCTTCCCTC
   ||| | | |||||||
TTAGGT-CGAAGGAAGAAGGGAA</td><td>3.34E-1</td></tr>
<tr><td>11. bta-miR-219-3p MIMAT0012547</td><td>6175</td><td>-10.60</td><td>CATGAGCAAGCTTCCCTCAATTCT
|| || |: || |||||||
GT-CT--ACAGGTCGGTGTTAAGA</td><td>3.34E-1</td></tr>
<tr><td>12. bta-miR-3578 MIMAT0016934</td><td>6911</td><td>-8.90</td><td>TTGGTAAGATCGCACAGTGGATTA
  :|| ||| |||||||
CTTCA-ACAAGC--ACCACCTAAG</td><td>2.28E-1</td></tr>
<tr><td>13. bta-miR-2284t-5p MIMAT0011831</td><td>8913</td><td>-5.70</td><td>CTCTGCTTG-CACTATGTTTG
     :|::| ||||||
TTTTTGGGCTCACTTACAAAG</td><td>2.69E-1</td></tr>
<tr><td>14. bta-miR-21-3p MIMAT0003745</td><td>9311</td><td>-11.40</td><td>TTGCTGGTGCTAT-ACTTGCTGTA
  :| :|: |:|| :||||||
TCTG-TCG-GGTAGCTGACGACAA</td><td>2.28E-1</td></tr>
</table>

<table>
<thead>
<tr><th>1. miR Name</th><th>leftmost position of predicted target site</th><th>folding energy (in - Kcal/mol)</th><th>heteroduplex</th><th>p value</th></tr>
</thead>
<tbody>
<tr><td>15. bta-miR-2406
MIMAT0011964</td><td>9500</td><td>-16.40</td><td>ATGCCACGCTTT--ATTTCCCTT
   ||| ||||:   :||||||
TTAGGT-CGAAGGAAGAAGGAA</td><td>3.48E-1</td></tr>
<tr><td>16. bta-miR-410
MIMAT0009311</td><td>10523</td><td>-6.50</td><td>AATTGCCCGTTCAGGATTATATA
    ||| || | ||||||
TGTCCGG--TAGACACAATATAA</td><td>8.51E-2</td></tr>
<tr><td>17. bta-miR-2396
MIMAT0011949</td><td>11761</td><td>-8.70</td><td>AATTGCTTGCAACATTTGCATGT
        :| || ||||||
GGTAGTTCTGGGGT-CACGTACT</td><td>3.25E-1</td></tr>
<tr><td>18. bta-miR-17-5p
MIMAT0003815</td><td>11801</td><td>-16.00</td><td>TGTGGCAATATTGTA-GCACTTTG
        :|:|||| ||||||||
TGATGGACGTGACATTCGTGAAAC</td><td>6.2E-2</td></tr>
<tr><td>19. bta-miR-200b
MIMAT0003842</td><td>11920</td><td>-8.80</td><td>AGCAAGTGCCTGACTAGTATTG
      |:|| :||||||
GTAGTAATGG-TCCGTCATAAT</td><td>1.75E-1</td></tr>
<tr><td>20. bta-miR-450b
MIMAT0024576</td><td>12557</td><td>-6.60</td><td>TATCTGCTACCGCTTTGCAAAA
          |: ||||||||
ATAAGTCCTTGTATAACGTTTT</td><td>3.29E-1</td></tr>
<tr><td>21. bta-miR-2284t-5p
MIMAT0011831</td><td>14999</td><td>-12.60</td><td>TATGACTGG-CAGAATGTTTC
   :||: | |||||||||
TTTTTGGGCTCACTTACAAAG</td><td>9.48E-2</td></tr>
<tr><td>22. bta-miR-455-3p
MIMAT0003836</td><td>16426</td><td>-14.60</td><td>TAGCTAGTTGTAAATGGACTGA
        ||: ||||||||
TCACATATACGGGTACCTGACG</td><td>3.57E-1</td></tr>
<tr><td>23. bta-miR-2483-3p
MIMAT0012076</td><td>16432</td><td>-9.50</td><td>GTTGTAAATGGACT-GATGTTG
      ||: :||: ||||||
AGAGAGTTGGTTGGTCTACAAA</td><td>3.57E-1</td></tr>
<tr><td>24. bta-miR-2285bz
MIMAT0046646</td><td>16731</td><td>-7.40</td><td>TATCGCGCCACAAC-CACTTATA
    :| ||| ||| ||||||
TTTTTG-GGT-TTGCTTGAATAA</td><td>8.13E-2</td></tr>
<tr><td>25. bta-miR-450b
MIMAT0024576</td><td>18487</td><td>-7.30</td><td>GTCTCCGCTAC--TTTGCAAAA
   || | || ||||||||
ATAAGTCCTTGTATAACGTTTT</td><td>1.95E-1</td></tr>
<tr><td>26. bta-miR-410
MIMAT0009311</td><td>18666</td><td>-5.30</td><td>TCAAGTAATCATGATTTATATT
 || |: ||| || |||||||
TGTCCGGTAG-ACACAATATAA</td><td>2.36E-2</td></tr>
<tr><td>27. bta-miR-3600
MIMAT0016932</td><td>19031</td><td>-12.80</td><td>TTTGTGTATGTTTTGGAACTGT
    ||: ||| :|||||||
TTCGACGGTCAACTTCTTGACA</td><td>2.42E-1</td></tr>
<tr><td>28. bta-miR-3956
MIMAT0025544</td><td>19867</td><td>-14.80</td><td>ATAATGGCGTTTA-CATTTCCACGA
    ||:| ||:| || ||||||
GGAGACTGGAAGTCGT--AGGTGCA</td><td>3.48E-1</td></tr>
</tbody>
</table>

<table>
<tr><th>1. miR Name</th><th>leftmost position of predicted target site</th><th>folding energy (in - Kcal/mol)</th><th>heteroduplex</th><th>p value</th></tr>
<tr><td>29. bta-miR-345-5p
MIMAT0003800</td><td>20031</td><td>-14.60</td><td>TTCGACAGCCTGAGAGTCAGC
         ||: ||||||||
TCGTGACCTGATCCTCAGTCG</td><td>7.09E-2</td></tr>
<tr><td>30. bta-miR-2448-3p
MIMAT0012026</td><td>20262</td><td>-13.70</td><td>GTTTATGG-TAATTTCAACCAG
: :|:|| |||||||
TGGGTGCCTAGGTTAGTTGGTG</td><td>1.95E-1</td></tr>
<tr><td>31. bta-miR-2415-5p
MIMAT0011978</td><td>20355</td><td>-13.40</td><td>GAGTTTGTTTCATACGACTCCA
  |: |: |||||||
ACCGGCCG-TCCTGGCTGAGGG</td><td>1.89E-1</td></tr>
<tr><td>32. bta-miR-450b
MIMAT0024576</td><td>21169</td><td>-5.80</td><td>TTTTC-TGCACA-AATGCAAAT
  ||| | ||| ||||||
ATAAGTCCTTGTATAACGTTTT</td><td>2.01E-1</td></tr>
<tr><td>33. bta-miR-2430
MIMAT0012000</td><td>21541</td><td>-12.00</td><td>TTAATTTTCC--ACTTACCCAGT
       :|| |: ||||||
GGCCTTTGGGGACGGCTGGGTCT</td><td>1.45E-1</td></tr>
<tr><td>34. bta-miR-219-3p
MIMAT0012547</td><td>21945</td><td>-11.40</td><td>TTCATAAACAGCATGCAATTCA
        |||| ::||||||
GTCTACAGGTCG-GTGTTAAGA</td><td>9.19E-2</td></tr>
<tr><td>35. bta-miR-2446
MIMAT0012023</td><td>22625</td><td>-8.00</td><td>GGCGAAGGTCAACAAAT-TATTTTT
||| |:| ||||| ||||||
CCG-GGCTA-AAGTTTATATAAAAT</td><td>1.86E-1</td></tr>
<tr><td>36. bta-miR-224
MIMAT0009271</td><td>24373</td><td>-10.10</td><td>TATTATGTCCTGCCTTTGACTTG
      | | | |||||||
ATTTGCCTTGGTGATCACTGAAC</td><td>2.98E-1</td></tr>
<tr><td>37. bta-miR-2284t-5p
MIMAT0011831</td><td>24935</td><td>-11.80</td><td>ATTTACCTGCTGCTAATGTTTC
    |||:| | ||||||||
TTTTTGGGC-TCACTTACAAAG</td><td>9.48E-2</td></tr>
<tr><td>38. bta-miR-2327
MIMAT0011856</td><td>25269</td><td>-18.49</td><td>TACAGGGCCTTACAAGTGCCCCCA
  ||| || :||||||
CGGTCACGAACTCAGAGCGGGGGA</td><td>1.04E-1</td></tr>
<tr><td>39. bta-miR-2285cr
MIMAT0046691</td><td>25659</td><td>-7.70</td><td>CTTAAC--AAACAGAACTTTT
   ||| ||| ||||||||
TCTTTGGACTTG-CTTGAAAA</td><td>7.54E-2</td></tr>
<tr><td>40. bta-miR-11999
MIMAT0046658</td><td>27145</td><td>-8.70</td><td>GCGAAGGTTAGTCCCGGTCTGT
|| | |:| ||||||
CGGATACTCTTATACCCAGACT</td><td>2.14E-1</td></tr>
<tr><td>41. bta-miR-21-3p
MIMAT0003745</td><td>27825</td><td>-17.20</td><td>TAATAGCTACCACAATGCCTGCTGTT
  |:||| ||| :| ||||||||
TCTGTCG--GGT--AGCTGACGACAA</td><td>2.25E-1</td></tr>
<tr><td>42. bta-miR-2285cr
MIMAT0046691</td><td>28049</td><td>-7.50</td><td>GGTAGACCT-TAT-AACTTTA
:| |:|||| |: ||||||
TC-TTTGGACTTGCTTGAAAA</td><td>2.5E-1</td></tr>
</table>

<table>
<tr><th>1. miR Name</th><th>leftmost position of predicted target site</th><th>folding energy (in -Kcal/mol)</th><th>heteroduplex</th><th>p value</th></tr>
<tr><td>43. bta-miR-455-3p MIMAT0003836</td><td>28718</td><td>-15.10</td><td>ACCAGTTTACACC-TGGACTGC
           || ||||||||
TCACATATACGGGTACCTGACG</td><td>1.17E-2</td></tr>
<tr><td>44. bta-miR-2468 MIMAT0012058</td><td>28739</td><td>-8.90</td><td>TGATGAAGCTATTAAATTCCTAA
||| | || ::| ||||||
ACTGTT-AGAAGGTACAAGGATA</td><td>1.17E-2</td></tr>
<tr><td>45. bta-miR-2284t-5p MIMAT0011831</td><td>28826</td><td>-6.40</td><td>TACAAGTCGCAGT--ATGTTTG
   || :|| ||| ||||||
TTTTTGGGC-TCACTTACAAAG</td><td>1.04E-1</td></tr>
<tr><td>46. bta-miR-2285cr MIMAT0046691</td><td>29373</td><td>-5.86</td><td>AGAAATAATATCTAAACTTTA
|||||: ||||||
TCTTTG-GACTTGCTTGAAAA</td><td>3.85E-1</td></tr>
<tr><td>47. bta-miR-12030 MIMAT0046723</td><td>29852</td><td>-20.60</td><td>GGCTGATGTCAATACCCCGGC
|||:| || ||||||
CCGGC--GAGGCCCGGGGCCC</td><td>1.98E-1</td></tr>
<tr><td>48. bta-miR-2285bu MIMAT0046640</td><td>29958</td><td>-14.10</td><td>ATTGAAGGCTCAGGAAGGTCTG
   :||:| ||| ||||||
GGTTTTTCAAGTAAGTCCAGAA</td><td>2.01E-1</td></tr>
<tr><td>49. bta-miR-17-5p MIMAT0003815</td><td>30467</td><td>-7.60</td><td>TAGATTTG-ACAGTA--CACTTTC
   |::|| || ||| ||||||
TGATGGACGTGACATTCGTGAAAC</td><td>3.29E-1</td></tr>
<tr><td>50. bta-miR-12006 MIMAT0046667</td><td>148</td><td>-12.60</td><td>CTGTT-AACAG-CTTTCAGCC
  ||| ||||| |||||||
CCCAACTTGTCTCAAAGTCGT</td><td>8.51E-2</td></tr>
<tr><td>51. bta-miR-2486-3p MIMAT0012082</td><td>1723</td><td>-6.82</td><td>GTAAAGGA-AACAAATCTCATTT
     :|| :|||||||
TTGTGTCTAAAACGGGGAGTAAA</td><td>3.11E-2</td></tr>
<tr><td>52. bta-miR-144 MIMAT0009234</td><td>1910</td><td>-8.10</td><td>TTAGTTTAGAAACTTTTACTGTT
:|||| :| | || ||||||
GATCATGTAGTAGA-TATGACAT</td><td>3.9E-1</td></tr>
<tr><td>53. bta-miR-2284p MIMAT0011885</td><td>2940</td><td>-8.20</td><td>TGATGCCATAGAAGAGAAACTTTCT
     || ||| ||||||||
TTTTAGG--GCTT--GTTTGAAAGT</td><td>2.42E-1</td></tr>
<tr><td>54. bta-miR-2356 MIMAT0011893</td><td>3262</td><td>-14.90</td><td>GTGGAGACATCTGATTCACAAGT
    |||| |: |||||||
GGAGTCTGAGGGAGTAGTGTTCT</td><td>2.65E-1</td></tr>
<tr><td>55. bta-miR-2284h-5p MIMAT0012019</td><td>3430</td><td>-7.40</td><td>TGCTGGTTGCGATCAGTTTTGGC
        :||| | ||||||
TTTGGGCTTGCT--TGAAAACCC</td><td>1.22E-2</td></tr>
<tr><td>56. bta-miR-12050 MIMAT0046753</td><td>4798</td><td>-13.50</td><td>GGGGGCATAGAT-ATATGCAGCCA
         ||: |:| |||||||
TCATCCTTCCTGCTGT-CGTCGGT</td><td>3.16E-1</td></tr>
</table>

1. miR Name	leftmost position of predicted target site	folding energy (in -Kcal/mol)	heteroduplex	p value
57. bta-miR-2284h-5p MIMAT0012019	4815	-11.50	CAGCCAGGATAAAGTTTTTGGT<br>|:|| |:| || :|||||| TTTGGGCTTGCTT-GAAAACCC	3.16E-1
58. bta-miR-2284h-5p MIMAT0012019	5493	-8.90	GGACGCTGTTATG-CATTTTGGT<br>:|| |:| |:| |||||| TTTG-GGC-TTGCTTGAAAACCC	2.86E-1
59. bta-miR-374b MIMAT0009302	5893	-10.00	TGTGACGGAGGTAAGTATTATAC<br>| |||| |||||||| GTGAATCGTCCA-ACATAATATA	6.48E-2
60. bta-miR-496 MIMAT0009336	5895	-6.30	TGACGGAGGTAAGTATTATACTCA<br>||| ||||||| CTCTAACCGGTACAT--TATGAGT	6.48E-2
61. bta-miR-2284h-5p MIMAT0012019	6134	-8.90	GGGGTTG-TATTACTTTTGGT<br>::: ::| |||||||| TTTGGGCTTGCTTGAAAACCC	2.14E-1
62. my-mir-noheader-provided-48	8371	-12.00	TGTAAAACTGGTTTGAAACTGAAGCTT<br>|:|| || :| || :||||||| CCGTTCTG-TC--TCTGGTGCTTCGAA	2.69E-1
63. bta-miR-12006 MIMAT0046667	8550	-12.60	ACACAAATCAGATTTTCAGCT<br>|||| ||||||| CCCAACTTGTCTCAAAGTCGT	5.51E-2
64. bta-miR-9-3p MIMAT0012549	9891	-7.10	TGGTAGTGATGTGCTTTAC<br>:||| :| |: |||||| GCCAATA-GAT-CGAAATA	1.42E-1
65. bta-miR-2285ai-5p MIMAT0040946	10237	-8.70	CGTACGCCAAAATATACATTTG<br>||||| |||||| TTTTTCGGTT-CGCTTGTAAAA	1.14E-1
66. bta-miR-2285e MIMAT0024580	10492	-15.10	TATGGTCCTTATAAGGATGCTCAGGTTG<br>|:| || ||| :| ||||||| TAATC-GG-TTTTTCAAGCAAGTCCAAA	8.51E-2
67. bta-miR-16b MIMAT0003525	10564	-12.50	GCATGGCTTTA--TGCTGCTA<br>|| |||| |||||||| CGGTTATAAATGCACGACGAT	8.51E-2
68. bta-miR-9-3p MIMAT0012549	10685	-7.90	TTGTTATAGATGCTTTAG<br>||||| |||||| GCCAATA-GATCGAAATA	3.07E-1
69. bta-miR-9-3p MIMAT0012549	11380	-7.60	TGG-ACAACAGCTTTAT<br>:|| |||||||| GCCAATAGATCGAAATA	1.86E-1
70. bta-miR-2329-3p MIMAT0011859	11436	-10.30	GAATGTT-TTGTATTTCACAGA<br>|:|: :|: ||||||| TGAATAGTCGAGTGTAGTGTCT	8.26E-2

<table>
<tr><th>1. miR Name</th><th>leftmost position of predicted target site</th><th>folding energy (in -Kcal/mol)</th><th>heteroduplex</th><th>p value</th></tr>
<tr><td>71. bta-miR-2451 MIMAT0012031</td><td>11444</td><td>-15.60</td><td>TGTATTTCACAGATATACCTCAA
| :|:|: |||| :||||||
AGGTGAGAGGTCT-CGTGGAGTA</td><td>8.26E-2</td></tr>
<tr><td>72. bta-let-7i MIMAT0003851</td><td>11446</td><td>-15.50</td><td>TATTTCACAGAT--ATACCTCA
     ||||:|: |||||||
TTGTCGTGTTTGATGATGGAGT</td><td>8.26E-2</td></tr>
<tr><td>73. bta-miR-2284h-5p MIMAT0012019</td><td>11658</td><td>-13.20</td><td>GCTTAATTTTAAGCTTTTGGG
           |:||||||||
TTTGGGCTTGCTTGAAAACCC</td><td>3.07E-1</td></tr>
<tr><td>74. bta-miR-2285ai-5p MIMAT0040946</td><td>11760</td><td>-9.00</td><td>TAATTGCT-TGC-AACATTTG
 || ||: || |||||||
TTTTTCGGTTCGCTTGTAAAA</td><td>3.25E-1</td></tr>
<tr><td>75. bta-miR-2349 MIMAT0011884</td><td>11907</td><td>-16.20</td><td>AGCTGCTGTGGATAGCAAGTGCCT
     ||| | :|| |||||||
ACTCAGACTCTGGTC-TTCACGGT</td><td>1.75E-1</td></tr>
<tr><td>76. bta-miR-9-3p MIMAT0012549</td><td>11975</td><td>-6.90</td><td>CTGTTTTGCAGGCTTTAC
  ||| | :||||||
GCCAA-TAGATCGAAATA</td><td>2.39E-1</td></tr>
<tr><td>77. bta-miR-12006 MIMAT0046667</td><td>13469</td><td>-8.10</td><td>TAATTGCTGCCG-TTTTCAGCG
   ||| :| | |||||||
CCCAAC-TTGTCTCAAAGTCGT</td><td>3.76E-1</td></tr>
<tr><td>78. bta-miR-2356 MIMAT0011893</td><td>14760</td><td>-14.40</td><td>GGGTGTATACCGGCATCACAAGT
         || |||||||||
GGAGTCTGAGGGAGTAGTGTTCT</td><td>2.35E-1</td></tr>
<tr><td>79. bta-miR-12011 MIMAT0046679</td><td>14827</td><td>-9.89</td><td>AATTTGGAAAAGCCAGGCTCTA
  ||||: ||||||
ACAAACTCAGAGTACCCGAGAG</td><td>6.08E-3</td></tr>
<tr><td>80. bta-miR-2329-3p MIMAT0011859</td><td>15514</td><td>-10.90</td><td>TTGATTCAACCTT-TGTCACAGA
     ||| ||: ::|||||||
TGAATAGT-CGAGTGTAGTGTCT</td><td>3.2E-1</td></tr>
<tr><td>81. bta-miR-2451 MIMAT0012031</td><td>15714</td><td>-11.90</td><td>GACAT-AAACAATGGACCTCAT
  ||: || | |||||||
AGGTGAGAGGTCTCGTGGAGTA</td><td>1.15E-2</td></tr>
<tr><td>82. bta-miR-2359 MIMAT0011897</td><td>16315</td><td>-8.30</td><td>GTGAAGATCATAAGCCACAATA
  :|||| |:|| ||||||
TTTTTCTTTTGTT--GTGTTAG</td><td>1.36E-1</td></tr>
<tr><td>83. bta-miR-453 MIMAT0009325</td><td>16723</td><td>-14.30</td><td>GTGTGTATTATCGCGCCACAACCA
       |:|:|:|| ||||||
ACGCTTGAGTGGTGC-CTGTTGGA</td><td>8.13E-2</td></tr>
<tr><td>84. bta-miR-2285ai-5p MIMAT0040946</td><td>16866</td><td>-5.40</td><td>AGTGTGCT--TGAGACATTTC
     ||:   :||||||
TTTTTCGGTTCGCTTGTAAAA</td><td>2.82E-1</td></tr>
</table>

<table>
<tr><th>1. miR Name</th><th>leftmost position of predicted target site</th><th>folding energy (in - Kcal/mol)</th><th>heteroduplex</th><th>p value</th></tr>
<tr><td>85. bta-miR-2285e
MIMAT0024580</td><td>19257</td><td>-10.21</td><td>GATGGC-ATGGATGCTAAGCAGGTTG
  |:|| ||||||
TAATCGGTTTTTCAAGCAAGTCCAAA</td><td>2.46E-1</td></tr>
<tr><td>86. bta-miR-2356
MIMAT0011893</td><td>19300</td><td>-17.10</td><td>CC-GCCACTT--GCATCACAAGA
|| |||: ||||||||||
GGAGTCTGAGGGAGTAGTGTTCT</td><td>2.46E-1</td></tr>
<tr><td>87. bta-miR-2285ai-5p
MIMAT0040946</td><td>19863</td><td>-8.00</td><td>TCTAATAATGGCGTTTACATTTC
         :||| ||||||
TTTTTCGGTTCGC--TTGTAAAA</td><td>3.48E-1</td></tr>
<tr><td>88. bta-miR-12006
MIMAT0046667</td><td>20006</td><td>-12.40</td><td>GGGTCAGGATGTCATCTTCAGCC
|||| |:|: ||||||
CCCA-ACTTG-TCTCAAAGTCGT</td><td>7.09E-2</td></tr>
<tr><td>89. bta-miR-2285bj
MIMAT0046391</td><td>21549</td><td>-10.09</td><td>CCACTTACCCAGTTTGAGGGTTT
||| :||: ||||||
GGTTTTTCAAGTAAG-TCCCAAC</td><td>1.45E-1</td></tr>
<tr><td>90. bta-miR-382
MIMAT0009308</td><td>21586</td><td>-11.60</td><td>AAGGTTTAC-AATTTCAACTTC
  ::|::|| |||||||
GCTTAGGTGGTGCTTGTTGAAG</td><td>1.34E-1</td></tr>
<tr><td>91. bta-miR-2285dg
MIMAT0046736</td><td>22028</td><td>-6.30</td><td>TGAAAAG--AAAGATGGTTTTT
  ||||| :||||||
GGTTTTCAAGTAAAGCCAAAAA</td><td>6.99E-2</td></tr>
<tr><td>92. bta-miR-2359
MIMAT0011897</td><td>22803</td><td>-9.70</td><td>TTTATAATGGCTCTGCACAATC
        ::| :|||||||
TTTTTCTTTTG--TTGTGTTAG</td><td>3.25E-1</td></tr>
<tr><td>93. bta-miR-2285e
MIMAT0024580</td><td>23833</td><td>-10.37</td><td>AATGGTTACTACCCTACTTCAGGTTC
   :|::| ||||||||
TAATCGGTTTTTCAAGCAAGTCCAAA</td><td>2.86E-1</td></tr>
<tr><td>94. bta-miR-4657
MIMAT0036970</td><td>24050</td><td>-12.00</td><td>GTGTGGTAGTACA--ACCACATA
 ||: :|| || ||||||
TACGGAGTCTGGTGAAGGTGTAA</td><td>1.2E-1</td></tr>
<tr><td>95. bta-miR-2285aj-5p
MIMAT0040947</td><td>24683</td><td>-10.10</td><td>GGGAACGTA--AGACCTTTTCA
 ::||| :| || |||||||
TTTTTGGGTTTGCTTGAAAAGT</td><td>2.18E-1</td></tr>
<tr><td>96. bta-miR-16b
MIMAT0003525</td><td>24763</td><td>-14.40</td><td>AATAATATT--GATGCTGCTA
  :|||||| ||||||||
CGGTTATAAATGCACGACGAT</td><td>1.75E-1</td></tr>
<tr><td>97. bta-miR-374b
MIMAT0009302</td><td>24914</td><td>-9.80</td><td>CAAGTTGTCAGTTGTATTATAA
          |||||||||||
GTGAATCGTCCAACATAATATA</td><td>9.48E-2</td></tr>
<tr><td>98. bta-miR-16b
MIMAT0003525</td><td>24929</td><td>-16.80</td><td>ATTATAATTTACCTGCTGCTA
      |||||| ||||||||
CGGTTATAAATGCACGACGAT</td><td>9.48E-2</td></tr>
</table>

<table>
<tr><th>1. miR Name</th><th>leftmost position of predicted target site</th><th>folding energy (in - Kcal/mol)</th><th>heteroduplex</th><th>p value</th></tr>
<tr><td>99. bta-miR-2349
MIMAT0011884</td><td>25263</td><td>-16.20</td><td>TAAATCTACAGGGCCTTACAAGTGCCC
      ||| :|:|| |||||||
ACTCAGA--CTCTGG--TCTTCACGGT</td><td>1.04E-1</td></tr>
<tr><td>100. bta-miR-1777a
MIMAT0012032</td><td>25272</td><td>-25.00</td><td>AGGGCCTTACAAGTGCCCCCA
    |||: || :|||||||
GGGCGGGGGGT-GGCGGGGGT</td><td>1.04E-1</td></tr>
<tr><td>101. bta-miR-2356
MIMAT0011893</td><td>25366</td><td>-10.00</td><td>CCTTGTACTTGC-CAACCACAAGC
         |||: | || ||||||
GGAGTCTGAGGGAGT-AGTGTTCT</td><td>1.16E-1</td></tr>
<tr><td>102. bta-miR-2284h-5p
MIMAT0012019</td><td>25378</td><td>-13.40</td><td>CAACCACAAGC--ATTTTTGGG
  |||| | |:| |:|||||||
TTTGG-GCTTGCTTGAAAACCC</td><td>1.16E-1</td></tr>
<tr><td>103. bta-miR-2285bc
MIMAT0046377</td><td>25628</td><td>-8.50</td><td>CTAA--TGGTAATCTCTATGGTTTTA
|:|| ||| || ||||||
GGTTTTCCCA-GTAAG--TCCAAAAA</td><td>2.65E-1</td></tr>
<tr><td>104. bta-miR-2285dg
MIMAT0046736</td><td>25628</td><td>-7.90</td><td>CTAATGGTAATCTCTATGGTTTTA
|:|| :|| || :||||||
GGTTTTCA--AGTAAAGCCAAAAA</td><td>2.65E-1</td></tr>
<tr><td>105. bta-miR-2329-3p
MIMAT0011859</td><td>26137</td><td>-13.90</td><td>GATTATGCAGCATGTAAATCACAGT
   |||| |||| :| |||||||
TGAATA-GTCG--AGTGTAGTGTCT</td><td>1.78E-1</td></tr>
<tr><td>106. bta-miR-496
MIMAT0009336</td><td>26185</td><td>-8.30</td><td>GACAATATTAATGCTATACTCA
               ||| |||||||
CTCTAACCGGTACATTATGAGT</td><td>4.38E-2</td></tr>
<tr><td>107. bta-miR-2329-3p
MIMAT0011859</td><td>26187</td><td>-14.10</td><td>CAATATTAATGCTATAC-TCACAGA
     |||:| ||| :|| |||||||
TGAATAGT--CGA-GTGTAGTGTCT</td><td>4.38E-2</td></tr>
<tr><td>108. bta-miR-2356
MIMAT0011893</td><td>26973</td><td>-7.90</td><td>AGTAAAATTTAGTGCAGCACAAGC
         |:|: || ||||||
GGAGTCTGAG-GGAGTAGTGTTCT</td><td>3.43E-1</td></tr>
<tr><td>109. bta-miR-935
MIMAT0009385</td><td>27253</td><td>-8.00</td><td>TACTACCCTGAACCCATAACTGG
              ||| | |||||||
CGCCATCGCCTTCGCCATTGACC</td><td>6.88E-2</td></tr>
<tr><td>110. bta-miR-2285ai-5p
MIMAT0040946</td><td>27326</td><td>-9.50</td><td>CGGATGTAATGCTGAACATTTC
        |: || ||||||||
TTTTTCGGTTCG-CTTGTAAAA</td><td>6.88E-2</td></tr>
<tr><td>111. my-mir-noheader-provided-48</td><td>27768</td><td>-8.90</td><td>TACATCCCTTCAATCATGTGAAGCTA
                 |:||: ||||||
CCGTTCTGTCTCTGGTG--CTTCGAA</td><td>2.11E-1</td></tr>
<tr><td>112. bta-miR-382
MIMAT0009308</td><td>29044</td><td>-10.60</td><td>TCAACCCAGAAACAAACAACTTG
        ||| || ||||||||
GCTTAGGT-GGTGCTTGTTGAAG</td><td>3.15E-2</td></tr>
</table>

<table>
<thead>
<tr><th>1. miR Name</th><th>leftmost position of predicted target site</th><th>folding energy (in - Kcal/mol)</th><th>heteroduplex</th><th>p value</th></tr>
</thead>
<tbody>
<tr><td>113. bta-miR-2285bj MIMAT0046391</td><td>29331</td><td>-8.82</td><td>CCA-TCAACCCA-AAAGGGTTC
||| || ||||||
GGTTTTTCAAGTAAGTCCCAAC</td><td>3.48E-1</td></tr>
<tr><td>114. bta-miR-362-5p MIMAT0009298</td><td>29376</td><td>-12.80</td><td>AATAATATCTAAACTTTAAGGATG
    |:|:||| |::||||||
TGAGTGTGGATCCAAGGTTCCTAA</td><td>3.85E-1</td></tr>
<tr><td>115. bta-let-7i MIMAT0003851</td><td>30051</td><td>-18.90</td><td>TCTGGCAACAGAACCCCTACCTCT
   :||| :||| |||||||
TTGTCGT--GTTTGATGATGGAGT</td><td>2.94E-1</td></tr>
<tr><td>116. bta-miR-12036 MIMAT0046731</td><td>30204</td><td>-15.20</td><td>CCCCGC--CAGA--AGAGGAGCCC
  ||:| || ||||||
CAGGTGCCTGCTCAGTGCCTCGGC</td><td>5.91E-3</td></tr>
<tr><td>117. bta-miR-2451 MIMAT0012031</td><td>30715</td><td>-14.30</td><td>CCTATACTGAAGA-CACCTCAG
  :|: || ||| |||||||
AGGTGAGAGGTCTCGTGGAGTA</td><td>2.39E-1</td></tr>
<tr><td>118. bta-miR-2349 MIMAT0011884</td><td>30928</td><td>-13.60</td><td>GAAGT-AATTGCCGAACAAGTGCCC
  ||| :||: |||||||
ACTCAGACTCTGGT--CTTCACGGT</td><td>6.29E-2</td></tr>
<tr><td>119. bta-miR-2285l MIMAT0024587</td><td>2210</td><td>-10.50</td><td>CTGCTCTTGCATTTGCATGGGTTTT
             ||| |||||||
CGGTTTTTCAAGTACG--CCCAAAA</td><td>2.61E-1</td></tr>
<tr><td>120. bta-miR-194b MIMAT0046730</td><td>2954</td><td>-11.40</td><td>AGAAAC--TTTCTCCATGTA
   ||| ||||||||
ACATTGTCGTTGAGGTACAC</td><td>2.42E-1</td></tr>
<tr><td>121. bta-miR-2285cf MIMAT0046663</td><td>5796</td><td>-10.00</td><td>TAAAAATTTGAAACAAACTTTT
 |||||:::||| ||||||
CTTTTTGGGCTT-GCTTGAAAC</td><td>2.39E-2</td></tr>
<tr><td>122. bta-miR-11997 MIMAT0046653</td><td>9640</td><td>-13.70</td><td>TCTTACTGCAGACAGCTTGGTA
|||: : || || ||||||
AGAGCCGGGTGTG-GGAACCAA</td><td>2.73E-1</td></tr>
<tr><td>123. bta-miR-2284i MIMAT0011792</td><td>9962</td><td>-5.04</td><td>AAATGGTTAATCCTACTTCTA
|||:: : ||||||
TTTGTGGTCTTGCTTGAAGAG</td><td>7.65E-2</td></tr>
<tr><td>124. bta-miR-194b MIMAT0046730</td><td>9978</td><td>-11.00</td><td>TTCTAAGGTAGAACCATGTA
      :|:|: ||||||
ACATTGTCGTTGAGGTACAC</td><td>7.65E-2</td></tr>
<tr><td>125. bta-miR-208a MIMAT0009261</td><td>10165</td><td>-9.60</td><td>CTAAGCCTTACAGTGATGTCTTAT
  |||| |: :|||||||
TGTTCG-AAAAACG-AGCAGAATA</td><td>3.56E-2</td></tr>
<tr><td>126. bta-miR-2284i MIMAT0011792</td><td>11831</td><td>-10.20</td><td>AAATACT--TGC-CACTTCTG
|||:||:   :| ||||||
TTTGTGGTCTTGCTTGAAGAG</td><td>6.2E-2</td></tr>
</tbody>
</table>

1. miR Name	leftmost position of predicted target site	folding energy (in - Kcal/mol)	heteroduplex	p value
127. bta-miR-2285cy MIMAT0046705	20278	-8.70	ACCAGAAGATTATTGGTGGTTTG<br>|:||| |:||| |||||| GTTTTTTCAAGTAA-GACCAAAA	1.95E-1
128. bta-miR-2482 MIMAT0012074	21415	-9.70	TTCTTGAAAAGGGTAAACTAC<br>:|| ||:|: ||||||| TTTTGCTGTTTCT-TTTGATG	1.45E-1
129. bta-miR-2284i MIMAT0011792	21589	-9.20	GTTTACAATTTC-AACTTCTC<br>:|| | |||||||| TTTGTGGTCTTGCTTGAAGAG	1.34E-1
130. bta-miR-2285cy MIMAT0046705	25627	-6.90	TCTAATGGTAATCTCTATGGTTTT<br>:|| || ||||||| GTTTTTTCA--AGTAAGACCAAAA	2.65E-1
131. bta-miR-2285cf MIMAT0046663	25658	-6.80	ACTTAAC--AAACAGAACTTTT<br>||| ||| ||||||| CTTTTTGGGCTTG-CTTGAAAC	7.54E-2
132. bta-miR-2285cy MIMAT0046705	27371	-11.90	AGGAAGAGTTGGATCAATGGTTTA<br>::||:|||| : || |||||| GTTTTTTCAAGTAAG--ACCAAAA	1.61E-2
133. bta-miR-2285cf MIMAT0046663	28049	-7.10	GGTAGACCT-TAT-AACTTTA<br>|: |:|||: |: |||||| CTTTTTGGGCTTGCTTGAAAC	2.5E-1
134. bta-miR-2464-3p MIMAT0012052	28105	-12.90	TAGTAATGGACA--TCTGGAG<br>:||| || || ||||||| GTCAAGACGAGTCGAGACCTC	3.03E-1
135. bta-miR-11997 MIMAT0046653	28227	-13.60	AGAGTTCCTAGTCATGCTTGGTG<br>||:| ||: |||||| AGAGCCGGGT--GTGGGAACCAA	2.65E-1
136. bta-miR-2285cf MIMAT0046663	29372	-6.16	GAGAAATAATATCTAAACTTTA<br>||:|||: |||||| CTTTTTG-GGCTTGCTTGAAAC	3.85E-1
137. bta-miR-2284i MIMAT0011792	29532	-11.10	AAGCA-AACTGC-TACTTCTC<br>||:|| :| ||||||| TTTGTGGTCTTGCTTGAAGAG	7.91E-3
138. bta-miR-105b MIMAT0009216	1723	-6.49	GTAAAGGAAACAAATCTCATTTGC<br>||||| |||||| TGGTTCCT--CAGACTCGTAAACT	3.11E-2
139. bta-miR-105b MIMAT0009216	2205	-7.90	TAGCACTGCTCT-TGCATTTGC<br>||| ||||||| TGGTTCCTCAGACTCGTAAACT	2.61E-1
140. bta-miR-2467-3p MIMAT0012057	3449	-23.60	TGGCAGTGATGCAGAAACTGCCCTG<br>:|| ||| || |||||||| GTCGGGACT-CG-GAGTGACGGGAG	1.22E-2

<table>
<tr><th>1. miR Name</th><th>leftmost position of predicted target site</th><th>folding energy (in -Kcal/mol)</th><th>heteroduplex</th><th>p value</th></tr>
<tr><td>141. bta-miR-545-3p
MIMAT0003807</td><td>3523</td><td>-14.00</td><td>CAGTATAGTCAGTTGTTTGTTGAT
|| :|:|:| |||||||||||
GT-GTGTTA-TTTACAAACAACTA</td><td>2.58E-1</td></tr>
<tr><td>142. bta-miR-12009
MIMAT0046677</td><td>3841</td><td>-9.30</td><td>AGTGTGTACAAAGC-AGCTTGTG
|||| | |:| ||||||
TCAC-C-TCCCTTGAACGAACAA</td><td>1.11E-1</td></tr>
<tr><td>143. bta-miR-2341
MIMAT0011876</td><td>5118</td><td>-13.80</td><td>TCAGAAGGAATTGCTTGCCTACTA
|||| || :|:||||||
AGTC-GGAAGAA-AGATGGATGAC</td><td>9.05E-2</td></tr>
<tr><td>144. bta-miR-2285de
MIMAT0046713</td><td>5795</td><td>-11.50</td><td>TTAAAAATTTGAAACAAACTTTT
  |||||::||||| |||||||
TCTTTTTGGACTTT-CTTGAAAA</td><td>2.39E-2</td></tr>
<tr><td>145. bta-miR-2285au
MIMAT0046346</td><td>5796</td><td>-10.00</td><td>TAAAAATTTGAAACAAACTTTTA
  ||||:::||| |||||||
TCTTTTGGGCTT-GCTTGAAAAA</td><td>2.39E-2</td></tr>
<tr><td>146. bta-miR-20a
MIMAT0003527</td><td>6922</td><td>-8.60</td><td>GCACAGTGGATTAAGAACACTTTT
  || || |:|| :| ||||||
GATG-GACGTGATATTCGTGAAAT</td><td>2.28E-1</td></tr>
<tr><td>147. bta-miR-2285au
MIMAT0046346</td><td>6926</td><td>-6.90</td><td>AGTGGATTAAGAAC--ACTTTTA
|| :|: |||| ||||||
TCTTTTG-GGCTTGCTTGAAAAA</td><td>2.28E-1</td></tr>
<tr><td>148. bta-miR-2285au
MIMAT0046346</td><td>7546</td><td>-6.20</td><td>TATAAACCAGGTAATACTTTTA
     ||| |: ||||||
TCTTTTGGGCTTGCTTGAAAAA</td><td>2.94E-1</td></tr>
<tr><td>149. bta-miR-9851
MIMAT0046371</td><td>8175</td><td>-15.00</td><td>AAGTTGTAATAACTTGGTGCCA
   ::|:   :||||||||
GGTGGCG-GTCACGACCACGGT</td><td>3.62E-1</td></tr>
<tr><td>150. bta-miR-2285cv
MIMAT0046699</td><td>8734</td><td>-5.30</td><td>GCTGTAGTAGACCAGGATTTTG
     ||| |||||||
TTTTTTCAAGTAAACCTAAAAG</td><td>3.52E-1</td></tr>
<tr><td>151. bta-miR-2285au
MIMAT0046346</td><td>9692</td><td>-7.00</td><td>AAATGGCTCTTAC-TACTTTTA
    ::|:| || ||||||
TCTTTTGGGCTTGCTTGAAAAA</td><td>1.98E-1</td></tr>
<tr><td>152. bta-miR-2341
MIMAT0011876</td><td>9900</td><td>-15.40</td><td>TGTGCTTTACCAACCGCCTACTG
   ||:|| | |||||||
AGTCGGAA-GAAAGATGGATGAC</td><td>1.42E-1</td></tr>
<tr><td>153. bta-miR-499
MIMAT0003536</td><td>10167</td><td>-12.30</td><td>AAGCCTTACAGTGATGTCTTAT
||:| |:|| | | ||||||
TTTGTAGTGACGTT-CAGAATT</td><td>3.56E-2</td></tr>
<tr><td>154. bta-miR-33a
MIMAT0009294</td><td>10176</td><td>-11.02</td><td>AGTGATGTCTTATCAAATGCAG
 | |||: ||||||
ACGTTACG-TTGATGTTACGTG</td><td>3.56E-2</td></tr>
</table>

<table>
<tr><th>1. miR Name</th><th>leftmost position of predicted target site</th><th>folding energy (in -Kcal/mol)</th><th>heteroduplex</th><th>p value</th></tr>
<tr><td>155. bta-miR-2285c MIMAT0011950</td><td>10497</td><td>-13.60</td><td>TCCTTATAAGGATGCTCAGGTTG
 || ||| || |||||||
CGG-TTTTTCAAACAAGTCCAAA</td><td>8.51E-2</td></tr>
<tr><td>156. bta-miR-376d MIMAT0010199</td><td>10692</td><td>-10.50</td><td>AGATGCTTTAGCTTCTATGAC
   || || |:|||||||
TACACCTAAAAGGAGATACTA</td><td>3.07E-1</td></tr>
<tr><td>157. bta-miR-33a MIMAT0009294</td><td>12325</td><td>-9.50</td><td>GGTTGTGTACCATTG-AATGCAA
 |: :||:| |:|: ||||||
ACGTTACGT--TGATGTTACGTG</td><td>9.78E-2</td></tr>
<tr><td>158. bta-miR-2341 MIMAT0011876</td><td>12633</td><td>-13.84</td><td>TCAGACTTGTAATACACCTACTC
|||| |:| |||||||
AGTC-GGAAGAAAGATGGATGAC</td><td>2.18E-1</td></tr>
<tr><td>159. bta-miR-2399-3p MIMAT0011955</td><td>12880</td><td>-7.60</td><td>ACAATTTCTTCTACAGTTAGAT
 || ||||| ||||||
TGTGAAAGACATCAACAATCTT</td><td>2.21E-1</td></tr>
<tr><td>160. bta-miR-499 MIMAT0003536</td><td>13216</td><td>-12.40</td><td>AAAGATC-CTGT--GTCTTAT
||| ||| |||: ||||||
TTTGTAGTGACGTTCAGAATT</td><td>3.29E-1</td></tr>
<tr><td>161. bta-miR-105b MIMAT0009216</td><td>13609</td><td>-6.20</td><td>AACACGATTTCTTTACATTTGA
         ||| |||||||
TGGTTCCTCAGACTCGTAAACT</td><td>2.73E-1</td></tr>
<tr><td>162. bta-miR-2341 MIMAT0011876</td><td>13765</td><td>-8.97</td><td>CTGGTTGTGAACAATCCTACTT
  :|:: ||||||
AGTCGGAAGAAAGATGGATGAC</td><td>1.32E-1</td></tr>
<tr><td>163. bta-miR-26a MIMAT0003516</td><td>13922</td><td>-5.90</td><td>AGGC-ATTTT--AACACTTGAT
|| | ||::| ||||||
TCGGATAGGACCTAATGAACTT</td><td>3.29E-1</td></tr>
<tr><td>164. bta-miR-2408 MIMAT0011966</td><td>15034</td><td>-13.10</td><td>AAAGTATAGC-AGCTACACGTG
       ||| :| |||||||
GGCCGACTCGAGTG-TGTGCAC</td><td>9.48E-2</td></tr>
<tr><td>165. bta-miR-10176-5p MIMAT0040931</td><td>18311</td><td>-24.40</td><td>GGCT-GTGGCTAAAGCTCCTCCT
::|| | ||| ||||||||
TTGAACGTCGA--GAGAGGAGGA</td><td>2.5E-1</td></tr>
<tr><td>166. bta-miR-376d MIMAT0010199</td><td>18934</td><td>-11.70</td><td>ATTTTGATTTTAAGTTCTATGAT
     |||||| :||||||||
TACACCTAAAA--GGAGATACTA</td><td>2.25E-1</td></tr>
<tr><td>167. bta-miR-12041 MIMAT0046737</td><td>20005</td><td>-10.50</td><td>AGGGTCAGGATGT--CATCTTCAGC
      ||| ||:   :||||||
TAAGTCTCC-ACGTTCCGGAAGTCA</td><td>7.09E-2</td></tr>
<tr><td>168. bta-miR-20a MIMAT0003527</td><td>21521</td><td>-8.90</td><td>AGACAAGC-CTA--ATCACTTTA
  || || ||| |||||||
GATGGACGTGATATTCGTGAAAT</td><td>1.45E-1</td></tr>
</table>

1. miR Name	leftmost position of predicted target site	folding energy (in - Kcal/mol)	heteroduplex	p value
169. bta-miR-545-3p MIMAT0003807	21679	-8.20	AAGACTATAGA--AGTGTTGAT<br>| || |||:| ||||||| GTGTGTTATTTACAAACAACTA	2.25E-1
170. bta-miR-376d MIMAT0010199	22099	-7.10	CCACTGATAAGCTAGCTATGAT<br>||| |: ||||||| TACACCTAAAAGG-AGATACTA	3.48E-1
171. bta-miR-1248 MIMAT0009972	22143	-11.70	GTGGTGCGTAAAGAGCTAGAAGAAGGT<br>|| |||||||| AAATCGTGTCACGAATATGTTCTTCCA	3.85E-1
172. bta-miR-124b MIMAT0013774	22664	-8.50	TTTACGCCTTATC--ATGCCTTT<br>|:|:| |||||| GAACCGTAAGTGGCGCACGGAAT	1.24E-1
173. bta-miR-33a MIMAT0009294	22673	-11.80	TATCATGCCTTTA-AATGCAC<br>|||| :|| ||||||| ACGTTACGTTGATGTTACGTG	1.24E-1
174. bta-miR-12009 MIMAT0046677	23253	-13.00	ACATGACGGCGGTTGCTTGTC<br>|| || : |||||||| TCACCTCCCTTGAACGAACAA	1.89E-1
175. bta-miR-2285de MIMAT0046713	25657	-7.90	TACTTAAC-AAACAGAACTTTT<br>||| ||||||||| TCTTTTTGGACTTTCTTGAAAA	7.54E-2
176. bta-miR-10176-5p MIMAT0040931	26567	-20.40	CCTCTGCTAGTCTGTTTCCTCCT<br>||| || || |:||||||| TTGAACG-TC-GAGAGAGGAGGA	3.46E-2
177. bta-miR-11978 MIMAT0046364	27359	-11.80	TCC--ATGATTTTAAGGAAGAGT<br>|| :|| |||||||| CGGCCGGCT-CCCTCCCTTCTCA	1.61E-2
178. bta-miR-2285de MIMAT0046713	28048	-8.60	AGGTAGACCT--TATAACTTTA<br>||: |:|||| |||||| TCTTTTTGGACTTTCTTGAAAA	2.5E-1
179. bta-miR-2285be MIMAT0046380	29025	-11.60	ACTGGAAGTT-GGTGGAGTTTC<br>::||||| | | |||||| GGTTTTTCAAACGAGCTCAAAA	3.15E-2
180. bta-miR-2285de MIMAT0046713	29371	-6.16	TGAGAAATAATATCTAAACTTTA<br>||:|||: |||||| TCTTTTTG-GACTTTCTTGAAAA	3.85E-1
181. bta-miR-11978 MIMAT0046364	29504	-16.00	TCAAACC-AGGGGTAGAAGAGC<br>|| ||||: |||||| CGGCCGGCTCCCTCCCTTCTCA	7.91E-3
182. bta-miR-2285be MIMAT0046380	29612	-10.10	TCAAAAAG--GAAAGGAGTTTG<br>:||||||| |||||| GGTTTTTCAAACGAGCTCAAAA	8.64E-2

<table>
<tr><th>1. miR Name</th><th>leftmost position of predicted target site</th><th>folding energy (in -Kcal/mol)</th><th>heteroduplex</th><th>p value</th></tr>
<tr><td>183. bta-miR-33a MIMAT0009294</td><td>30219</td><td>-12.90</td><td>AGC--CCCAATAAACAATGCAC
 || |||: |||||||||
ACGTTACGTTG-ATGTTACGTG</td><td>5.91E-3</td></tr>
<tr><td>184. bta-miR-2368-3p MIMAT0011912</td><td>30461</td><td>-11.22</td><td>TGCA-ATTAGATTTGACAGT
 ||| |:|||||||
CCGTCTCCACCAGACTGTCA</td><td>3.29E-1</td></tr>
<tr><td>185. bta-miR-6501 MIMAT0046685</td><td>3451</td><td>-19.60</td><td>GCAGT-GATGCAGAAACTGCCCTGT
:| || ::||| ||||||||
TGACAATGGTGTC--CGACGGGACC</td><td>1.22E-2</td></tr>
<tr><td>186. bta-miR-500 MIMAT0009337</td><td>8730</td><td>-12.10</td><td>TGTTGCTGTAGTAGACCAGGATTT
  | |: |||| | ||||||
AGAGTGGGTCCATC-GTTCCTAAT</td><td>3.52E-1</td></tr>
<tr><td>187. bta-miR-2285az MIMAT0046356</td><td>8733</td><td>-8.60</td><td>TGCTGTAGTAGACCAGGATTTT
      ||| || |||||||
GGTTTTTCAAGTGAGCCTAAAA</td><td>3.52E-1</td></tr>
<tr><td>188. bta-miR-2285az MIMAT0046356</td><td>10469</td><td>-7.50</td><td>GTACTGATTTCAATGGGGATTTT
        |||| |||||||
GGTTTTTCAAGT-GAGCCTAAAA</td><td>8.51E-2</td></tr>
<tr><td>189. bta-miR-500 MIMAT0009337</td><td>10523</td><td>-12.20</td><td>AATTGCCC--GT--TCAGGATTA
  | ||| || |||||||
AGAGTGGGTCCATCGTTCCTAAT</td><td>8.51E-2</td></tr>
<tr><td>190. bta-miR-500 MIMAT0009337</td><td>13086</td><td>-12.80</td><td>TGCTACC--ACTAGTCAGGATTC
   :||| |||: ||||||
AGAGTGGGTCCATCGTTCCTAAT</td><td>1.38E-1</td></tr>
<tr><td>191. bta-miR-181a MIMAT0003543</td><td>14995</td><td>-7.80</td><td>GTACTATGACTGGC--AGAATGTT
           :|| |||||||
TTGAGTGGCTGTCGCAACTTACAA</td><td>9.48E-2</td></tr>
<tr><td>192. bta-miR-6501 MIMAT0046685</td><td>22521</td><td>-16.90</td><td>ATATGGACCTTAATCCTGCCCTGT
      ||| ||||||||
TGACAATGG-TGTCCGACGGGACC</td><td>3.66E-1</td></tr>
<tr><td>193. bta-miR-181a MIMAT0003543</td><td>26995</td><td>-7.80</td><td>GCTATGGAGAAGGTTAATGAATGTG
        || :|: |||||||
TTGAGTGGCTGTCG-CAACTTACAA</td><td>1.75E-1</td></tr>
<tr><td>194. bta-miR-2373-3p MIMAT0011919</td><td>232</td><td>-22.70</td><td>GGTCTCGAACTAC--ACTGGGCTCC
|| ||| |:|| |:||||||
CC-GAG-AAGGTGCACGGCCCGAGC</td><td>8.01E-2</td></tr>
<tr><td>195. bta-miR-2904 MIMAT0013862</td><td>237</td><td>-15.80</td><td>CGAACTACACTG-GGCTCCA
    |:| ||:| ||||||
CTCCGGT-TGGCTCCGAGGG</td><td>8.01E-2</td></tr>
<tr><td>196. bta-miR-217 MIMAT0009267</td><td>409</td><td>-11.80</td><td>AAACAA-GAGT-GTTGTGTGCAGTC
   ||| ||| :|| :||||||
TAGGTTAGTCAAGGAC-TACGTCAT</td><td>1.45E-1</td></tr>
</table>

<table>
<tr><th>1. miR Name</th><th>leftmost position of predicted target site</th><th>folding energy (in -Kcal/mol)</th><th>heteroduplex</th><th>p value</th></tr>
<tr><td>197. bta-miR-217
MIMAT0009267</td><td>487</td><td>-9.50</td><td>CTTCAA--GATGCTT--TGCAGTC
|:||| |:| ||||||
TAGGTTAGTCAAGGACTACGTCAT</td><td>3.03E-1</td></tr>
<tr><td>198. bta-miR-2442
MIMAT0012017</td><td>2196</td><td>-16.40</td><td>TTTTACTACTAGC-ACTGCTCT
:||| |||||||
ACGTCGGGTGTCGGGGACGAGA</td><td>2.61E-1</td></tr>
<tr><td>199. bta-miR-1434-5p
MIMAT0012039</td><td>2952</td><td>-7.70</td><td>AGAGA-AACTTTCTCCATGTAA
|||:| || ||||||
TCTTTAAAATCAGTAGTACATG</td><td>2.42E-1</td></tr>
<tr><td>200. bta-miR-2352
MIMAT0011888</td><td>3517</td><td>-9.56</td><td>AAGCAA-C-AGTATAGTCAGTTG
||||| | ||||||
GTCGTTAGGACAAGAAAGTCAAA</td><td>2.58E-1</td></tr>
<tr><td>201. bta-miR-217
MIMAT0009267</td><td>4139</td><td>-9.20</td><td>TTGCAA--AG--GCTATTGCAGTA
| ||| || || |||||||
TAGGTTAGTCAAGGACTACGTCAT</td><td>1.02E-1</td></tr>
<tr><td>202. bta-miR-2285dm
MIMAT0046758</td><td>6055</td><td>-12.80</td><td>TATAAGGTTACTGAGTGGCCAA
||| |||||||
TTTTATGTCTGA-AAACCGGTT</td><td>4.26E-2</td></tr>
<tr><td>203. bta-miR-3602
MIMAT0016937</td><td>6062</td><td>-13.40</td><td>TTACTG-AGTGG--CCAACAG
|||:| |||: ||||||
AATGGCGCCACTAGGGTTGTG</td><td>4.26E-2</td></tr>
<tr><td>204. bta-miR-1284
MIMAT0009955</td><td>8252</td><td>-13.40</td><td>ATTCTGCTAAG-CATGTGCAGG
||:| | |||||||
CTTTTCGGTCCCAGACACGTCT</td><td>3.07E-1</td></tr>
<tr><td>205. bta-miR-2444
MIMAT0012021</td><td>8535</td><td>-5.80</td><td>GCCCACTTACACAGTACACAAA
||| |||||||
TTTTGTTTTTTGT--TGTGTTT</td><td>5.51E-2</td></tr>
<tr><td>206. bta-miR-106b
MIMAT0009218</td><td>8798</td><td>-11.80</td><td>GTTACCATGTGTT-GCACTTTA
||: |||: ||||||||
TAGACGTG-ACAGTCGTGAAAT</td><td>1.54E-1</td></tr>
<tr><td>207. bta-miR-1284
MIMAT0009955</td><td>8831</td><td>-10.90</td><td>TTTCTGCTGATGG--AGTGCAGT
||: || ||||||
CTTTTCGG-TCCCAGACACGTCT</td><td>1.54E-1</td></tr>
<tr><td>208. bta-miR-3533
MIMAT0036975</td><td>9922</td><td>-13.40</td><td>GCTTCCGTTTCA-ACTTCAT
|||: ||| |||||||
TACAGGTGCAGTGTGAAGTA</td><td>7.65E-2</td></tr>
<tr><td>209. bta-miR-1603
MIMAT0011823</td><td>10295</td><td>-13.20</td><td>CTGCTTATAACGGCAAACCAC
:|:|| ::||||||||
TTTTTGTGTT-TTGTTTGGTG</td><td>3.62E-1</td></tr>
<tr><td>210. bta-miR-222
MIMAT0003530</td><td>10548</td><td>-8.02</td><td>ATCT-GT-TAATTTTGTAGCA
|:|: || ||||||
TGGGTCATCGGTCTACATCGA</td><td>8.51E-2</td></tr>
</table>

<table>
<tr><th>1. miR Name</th><th>leftmost position of predicted target site</th><th>folding energy (in -Kcal/mol)</th><th>heteroduplex</th><th>p value</th></tr>
<tr><td>211. bta-miR-1284
MIMAT0009955</td><td>16070</td><td>-8.13</td><td>GAACATGTATTTAAGAAGTGCAGT
||| |: ||||||
CTT-TTCG-GTCCCAGACACGTCT</td><td>3.52E-1</td></tr>
<tr><td>212. bta-miR-2371
MIMAT0011916</td><td>16884</td><td>-7.10</td><td>CAGAACAATGTTGTGAACTATC
 |:||| | :   :||||||
TTTTTGGTGTG--TTTTGATAT</td><td>2.82E-1</td></tr>
<tr><td>213. bta-miR-2442
MIMAT0012017</td><td>17285</td><td>-13.10</td><td>TGTTTATATTGGTGATCCTGCTCA
          :|: :|||||||
ACGTCGGGTGTCG--GGGACGAGA</td><td>3.52E-1</td></tr>
<tr><td>214. bta-miR-2405
MIMAT0011963</td><td>19156</td><td>-12.80</td><td>ACAAACATGCATTCCACACT
     || :|| ||||||
CTCCGGTTTGTTTGGTGTGT</td><td>3.16E-1</td></tr>
<tr><td>215. bta-miR-411a
MIMAT0009312</td><td>19381</td><td>-7.80</td><td>CTTAC--AATACAG-CTACTAC
  ||| |||| | ||||||
GCATGCGATATGCCAGATGATA</td><td>3.12E-1</td></tr>
<tr><td>216. bta-miR-2352
MIMAT0011888</td><td>20519</td><td>-7.10</td><td>TGTTGATTTTAAAGATTTTCAGTTC
    :||::| :||||||||
GTCGTTAGGA--CAAGAAAGTCAAA</td><td>3.16E-1</td></tr>
<tr><td>217. bta-miR-106b
MIMAT0009218</td><td>21523</td><td>-5.90</td><td>ACAAGC-CT--AATCACTTTA
    || || |||||||
TAGACGTGACAGTCGTGAAAT</td><td>1.45E-1</td></tr>
<tr><td>218. bta-miR-106b
MIMAT0009218</td><td>22584</td><td>-7.30</td><td>TTTTTAGGAGTTTTCACTTTA
         ||: |||||||
TAGACGTGACAGTCGTGAAAT</td><td>3.43E-1</td></tr>
<tr><td>219. bta-miR-375
MIMAT0009303</td><td>22618</td><td>-12.70</td><td>TTACAC-AGGCGAAGGTCAACAAAT
|:|| | || |||| | ||||||
AGTGCGCTCGGCTTGC--TTGTTTT</td><td>1.86E-1</td></tr>
<tr><td>220. bta-miR-3533
MIMAT0036975</td><td>23837</td><td>-7.70</td><td>GTTACTAC--CCTACTTCAG
    |:|| :||||||
TACAGGTGCAGTGTGAAGTA</td><td>2.86E-1</td></tr>
<tr><td>221. bta-miR-2371
MIMAT0011916</td><td>24798</td><td>-9.60</td><td>TTTTTCCA-GCATAACTATA
     ||| :|| |||||||
TTTTTGGTGTGTTTTGATAT</td><td>3.85E-1</td></tr>
<tr><td>222. bta-miR-217
MIMAT0009267</td><td>24861</td><td>-12.90</td><td>GGGCAATTTGGGCTATTTGCAGTC
   ||||: | |: ||||||
TAGGTTAGTCAAGGACTACGTCAT</td><td>2.32E-1</td></tr>
<tr><td>223. bta-miR-1434-5p
MIMAT0012039</td><td>26132</td><td>-5.50</td><td>GTGGTGATTATGCAGCATGTAA
       ||| || ||||||
TCTTTAAAATCAGTAGTACATG</td><td>1.78E-1</td></tr>
<tr><td>224. bta-miR-1284
MIMAT0009955</td><td>26969</td><td>-9.30</td><td>CACTAGTAAAATTTAGTGCAGC
    ||: |: ||||||
CTTTTCGGTCCCAGACACGTCT</td><td>3.43E-1</td></tr>
</table>

<table>
<tr><th>1. miR Name</th><th>leftmost position of predicted target site</th><th>folding energy (in - Kcal/mol)</th><th>heteroduplex</th><th>p value</th></tr>
<tr><td>225. bta-miR-1603
MIMAT0011823</td><td>28633</td><td>-10.10</td><td>TTTTACAACGATGTAAAACCAC
||| |:| |||||||
TTTTTGT-GTT-TTGTTTGGTG</td><td>1.78E-1</td></tr>
<tr><td>226. bta-miR-2442
MIMAT0012017</td><td>29961</td><td>-16.50</td><td>GAAGGCTCA-GGAAGGTCTGCTCC
:||:|| :| :||||||
ACGTCGGGTGTC--GGGGACGAGA</td><td>2.01E-1</td></tr>
<tr><td>227. bta-miR-3533
MIMAT0036975</td><td>29985</td><td>-12.30</td><td>AATTCCAGATC-TACTTCAC
|||| || :||||||
TACAGGTGCAGTGTGAAGTA</td><td>2.01E-1</td></tr>
<tr><td>228. bta-miR-2378
MIMAT0011924</td><td>30301</td><td>-15.20</td><td>AACTTGGAACTAGTGACCCACA
|||| | : |||||||
GGGAAC-GAGTCTGTTGGGTGT</td><td>4.98E-2</td></tr>
<tr><td>229. bta-miR-2285dm
MIMAT0046758</td><td>30376</td><td>-13.40</td><td>CAAGATTAGA--GTTGGCCAA
:||| ||||||||
TTTTATGTCTGAAAACCGGTT</td><td>1.09E-1</td></tr>
<tr><td>230. bta-miR-1284
MIMAT0009955</td><td>30381</td><td>-16.70</td><td>TTAGAGTTGGCCAAAGTGCAGA
|:||: | |||||||
CTTTTCGGTCCCAGACACGTCT</td><td>1.09E-1</td></tr>
<tr><td>231. bta-miR-1603
MIMAT0011823</td><td>30555</td><td>-12.10</td><td>ATGAATATGAGTCCAAAACCAC
:||:| |: |||||||
TTTTTGTGTTT--TGTTTGGTG</td><td>3.66E-2</td></tr>
<tr><td>232. bta-miR-12055
MIMAT0046762</td><td>266</td><td>-18.90</td><td>GGATGTTTGAGGACGCAGAGGAGAA
:|||| || ||||||||
TCAAGAGGCTCC--CG-CTCCTCTT</td><td>3.03E-1</td></tr>
<tr><td>233. bta-miR-2354
MIMAT0011890</td><td>5122</td><td>-12.50</td><td>AAGGAATTGC-TTGCCTACTA
:||: :| :|||||||
TGATTTGTTGTGTTGGATGAT</td><td>9.05E-2</td></tr>
<tr><td>234. bta-miR-10163-3p
MIMAT0040910</td><td>5862</td><td>-13.10</td><td>TGAGTATAAACC-TGACTTGTCA
|||| || |||||||
TCTCTCTCTTGGTAC-GAACAGT</td><td>6.48E-2</td></tr>
<tr><td>235. bta-miR-671
MIMAT0009365</td><td>6168</td><td>-14.39</td><td>GTTAAGCCATGAGCAAGCTTCCC
|||| ||||||
GAGGTCGGGGAGGTCCCGAAGGA</td><td>3.34E-1</td></tr>
<tr><td>236. bta-miR-671
MIMAT0009365</td><td>8555</td><td>-14.70</td><td>AATCAG-ATTTTCA--GCTTCCC
:||| ::|:|| ||||||
GAGGTCGGGGAGGTCCCGAAGGA</td><td>5.51E-2</td></tr>
<tr><td>237. bta-miR-2304
MIMAT0011816</td><td>8939</td><td>-10.80</td><td>CCGATGGTAGTC-CACAACC
:| | |||||||
TGTGTGTGTTGGTGTGTTGG</td><td>3.62E-1</td></tr>
<tr><td>238. bta-miR-3596
MIMAT0016940</td><td>9405</td><td>-13.00</td><td>TGTTAATGTG-ATTGTGTGGTG
|| ||: |||||||||
ACTCCATCATCCAACACACCAA</td><td>1.92E-1</td></tr>
</table>

<table>
<tr><th>1. miR Name</th><th>leftmost position of predicted target site</th><th>folding energy (in - Kcal/mol)</th><th>heteroduplex</th><th>p value</th></tr>
<tr><td>239. bta-miR-2354
MIMAT0011890</td><td>9959</td><td>-8.70</td><td>TGAAAATGGTTAATCCTACTT
|||:   : ||||||
TGATTTGTTGTGTTGGATGAT</td><td>7.65E-2</td></tr>
<tr><td>240. bta-miR-2354
MIMAT0011890</td><td>12637</td><td>-14.20</td><td>ACT-TGTAATACA-CCTACTC
||| :||:||| ||||||
TGATTTGTTGTGTTGGATGAT</td><td>2.18E-1</td></tr>
<tr><td>241. bta-miR-2354
MIMAT0011890</td><td>13764</td><td>-12.90</td><td>GCTGGTTGTGAACAATCCTACTT
:||:: |||| ||||||
TGATT-TGTTGTGTT-GGATGAT</td><td>1.32E-1</td></tr>
<tr><td>242. bta-miR-3596
MIMAT0016940</td><td>13988</td><td>-11.60</td><td>TGCAGCCCCAGG--GTGTGGTG
||| |||||||
ACTCCATCATCCAACACACCAA</td><td>3.48E-1</td></tr>
<tr><td>243. bta-miR-2354
MIMAT0011890</td><td>19606</td><td>-18.10</td><td>ATAATACAACATATCCTACTA
||||||:| |||||||
TGATTTGTTGTGTTGGATGAT</td><td>5.58E-3</td></tr>
<tr><td>244. bta-miR-664a
MIMAT0012078</td><td>20024</td><td>-17.70</td><td>CAGCCA-ATTCG--ACAGCCTG
||| |: |||||||
GTAGGTGTGTGTGGGGTCGGAC</td><td>7.09E-2</td></tr>
<tr><td>245. bta-miR-664a
MIMAT0012078</td><td>21655</td><td>-14.70</td><td>TTGCTATGCTGGATATTCAGCCTG
:|::| ::|||||||
GTAGGTGTG--TGTGGGGTCGGAC</td><td>2.25E-1</td></tr>
<tr><td>246. bta-miR-10163-3p
MIMAT0040910</td><td>23252</td><td>-15.50</td><td>AACATGACGGCGGTTGCTTGTCA
|| :| :| ||||||||
TCTCTCTCTTGGTA-CGAACAGT</td><td>1.89E-1</td></tr>
<tr><td>247. bta-miR-2354
MIMAT0011890</td><td>23829</td><td>-10.00</td><td>GCTTAATGGTTACTACCCTACTT
:|| ||:   : || ||||||
TGATTTGTTG-TG-TTGGATGAT</td><td>2.86E-1</td></tr>
<tr><td>248. bta-miR-664a
MIMAT0012078</td><td>24709</td><td>-12.10</td><td>AATTTTAATATG-AGCAGCCTG
|:|:: |||||||
GTAGGTGTGTGTGGGGTCGGAC</td><td>1.75E-1</td></tr>
<tr><td>249. bta-miR-2365
MIMAT0011903</td><td>27376</td><td>-5.70</td><td>GAGTTGGATCAATGGTTTAAAAACC
|| :|| ||::|: ||||||
CT-ATTCT--TTGTCG--TTTTTGA</td><td>1.61E-2</td></tr>
<tr><td>250. bta-miR-137
MIMAT0009231</td><td>27473</td><td>-7.94</td><td>ATAGGT--TACAGGAGGCAATAA
|| |: |:|||||||
GATGCGCATAAGAATTCGTTATT</td><td>2.14E-1</td></tr>
<tr><td>251. bta-miR-12024
MIMAT0046717</td><td>29969</td><td>-6.80</td><td>AGGAAGGTCTG-CTCCTAAT
|| || || ||||||
TCACAACAAACCTAGGATTT</td><td>2.01E-1</td></tr>
<tr><td>252. bta-miR-1584-5p
MIMAT0011969</td><td>30207</td><td>-20.50</td><td>CGCCAGAAGAGGAGCCCCAA
||| || ||||||||
ACGGGGGTCGGGTCGGGGTT</td><td>5.91E-3</td></tr>
</table>

<table>
<tr><th>1. miR Name</th><th>leftmost position of predicted target site</th><th>folding energy (in -Kcal/mol)</th><th>heteroduplex</th><th>p value</th></tr>
<tr><td>253. bta-miR-12055 MIMAT0046762</td><td>30589</td><td>-10.40</td><td>GTCAGAAGAATGGACAAGGAGAA
           || | |||||||
TCAAGAGGCTCCC-GCTCCTCTT</td><td>4.57E-2</td></tr>
<tr><td>254. bta-miR-664a MIMAT0012078</td><td>30673</td><td>-15.80</td><td>TGACTGCAGAGGACATCAGCCTT
   | || : || ||||||
GTAGGTGT-GTGTGGGGTCGGAC</td><td>1.28E-1</td></tr>
<tr><td>255. bta-miR-29b MIMAT0003828</td><td>2237</td><td>-13.90</td><td>TTCAT--GTTTTGCATGGTGCTT
  ||:   :|||:: ||||||||
TTGTGACTAAAGTTTACCACGAT</td><td>2.61E-1</td></tr>
<tr><td>256. bta-miR-502b MIMAT0009339</td><td>2649</td><td>-11.40</td><td>TAAAATTTGC-ATTGTGGATA
     :|||| ||||||
CTTAGGAACGGGTCCACCTAA</td><td>2.78E-1</td></tr>
<tr><td>257. bta-miR-502b MIMAT0009339</td><td>2893</td><td>-7.30</td><td>GAAGTGGATGATAC-TGTGGATA
||| || | ||||||
CTT-AGGAAC-GGGTCCACCTAA</td><td>1.36E-1</td></tr>
<tr><td>258. bta-miR-539 MIMAT0009342</td><td>2943</td><td>-10.60</td><td>TGCCATAGAAGAGAAACTTTCTCC
   ||: ||| || | |||||||
TGTGTG-GTTC-CTATTAAAGAGG</td><td>2.42E-1</td></tr>
<tr><td>259. bta-miR-502b MIMAT0009339</td><td>3852</td><td>-12.40</td><td>AGCAGCTTGTGT-TGTGGATG
     ||||: : ||||||
CTTAGGAACGGGTCCACCTAA</td><td>1.11E-1</td></tr>
<tr><td>260. bta-miR-2285w MIMAT0029945</td><td>4815</td><td>-7.00</td><td>CAGCCAGGATAAAGTTTTTGGT
      |:|| || :|||||||
TTTAGACTTACTT-GAAAACCA</td><td>3.16E-1</td></tr>
<tr><td>261. bta-miR-2285w MIMAT0029945</td><td>5494</td><td>-9.20</td><td>GACGCTGTTATG-CATTTTGGT
    ||| ||| |||||||
TTTAGAC-TTACTTGAAAACCA</td><td>2.86E-1</td></tr>
<tr><td>262. bta-miR-2404 MIMAT0011961</td><td>5647</td><td>-11.10</td><td>AAGGGTGTAG-GAAGTGCAA
  :|:|:   : |||||||
CGTCTATGGTACGTCACGTT</td><td>9.48E-2</td></tr>
<tr><td>263. bta-miR-2284u MIMAT0011895</td><td>5795</td><td>-6.60</td><td>TTAAAAATTTGAAACAAACTTTT
  |||||:: ||| |||||||
TCTTTTTGG-CTT-GCTTGAAAA</td><td>2.39E-2</td></tr>
<tr><td>264. bta-miR-2285w MIMAT0029945</td><td>6134</td><td>-10.00</td><td>GGGGTTG-TATTACTTTTGGT
    :|| |||||||||
TTTAGACTTACTTGAAAACCA</td><td>2.14E-1</td></tr>
<tr><td>265. bta-miR-29b MIMAT0003828</td><td>6786</td><td>-13.00</td><td>GAGTGTGGTTGCTAGA-GGTGCTT
     ||:|| :|:| ||||||
TTGTGACTAA-AGTTTACCACGAT</td><td>3.52E-1</td></tr>
<tr><td>266. bta-miR-502b MIMAT0009339</td><td>9268</td><td>-12.90</td><td>TTAGCACAGCCT--GTGGATT
        |||: |||||||
CTTAGGAACGGGTCCACCTAA</td><td>2.28E-1</td></tr>
</table>

<table>
<tr><th>1. miR Name</th><th>leftmost position of predicted target site</th><th>folding energy (in -Kcal/mol)</th><th>heteroduplex</th><th>p value</th></tr>
<tr><td>267. bta-miR-29b MIMAT0003828</td><td>9298</td><td>-13.90</td><td>ACTGCTAGTTCCATTGCTGGTGCTA
:|| :|| || ||||||||
TTGTGACTAAAGT--TTACCACGAT</td><td>2.28E-1</td></tr>
<tr><td>268. bta-miR-2285w MIMAT0029945</td><td>9325</td><td>-7.70</td><td>CTTGCTGTAATTGTTGTTTTGGT
||| || |||||||
TTTAGAC--TTACTTGAAAACCA</td><td>2.28E-1</td></tr>
<tr><td>269. bta-miR-33b MIMAT0009295</td><td>10177</td><td>-11.60</td><td>GTGATGTCTTATCAAATGCAG
| |||: :| ||||||
CGTTACGTTGT-CGTTACGTG</td><td>3.56E-2</td></tr>
<tr><td>270. bta-miR-497 MIMAT0004343</td><td>10561</td><td>-12.10</td><td>GTAGCATGGCTTTATGCTGCTA
|: :| |||||||
ATGTTTGGTGTCACACGACGAC</td><td>8.51E-2</td></tr>
<tr><td>271. bta-miR-106a MIMAT0003784</td><td>11803</td><td>-13.40</td><td>TGGCAATATTGTA-GCACTTTG
:|:|||| |||||||
ATGGACGTGACATTCGTGAAAA</td><td>6.2E-2</td></tr>
<tr><td>272. bta-miR-33b MIMAT0009295</td><td>12326</td><td>-10.10</td><td>GTTGTGTACCATTGAATGCAA
|: :||:| || ||||||
CGTTACGTTGT-CGTTACGTG</td><td>9.78E-2</td></tr>
<tr><td>273. bta-miR-2285w MIMAT0029945</td><td>13958</td><td>-7.00</td><td>TAAATGGTATGA--TTTTGGT
|||| |||||||
TTTAGACTTACTTGAAAACCA</td><td>3.48E-1</td></tr>
<tr><td>274. bta-miR-497 MIMAT0004343</td><td>14412</td><td>-15.80</td><td>GACTTGCTTTTATATGCTGCTG
|| :|: ||||||||
ATGTTTGGTGTCACACGACGAC</td><td>7.54E-2</td></tr>
<tr><td>275. bta-miR-11986 MIMAT0046385</td><td>14680</td><td>-8.70</td><td>ATTTGCCCAC-CATGGTGGACAT
:| || ||| ||||||
TTCCTGATTGAGTA--ACCTGTT</td><td>1.98E-2</td></tr>
<tr><td>276. bta-miR-10169-3p MIMAT0040917</td><td>16152</td><td>-11.56</td><td>AGTTGCAT-CAGAAAGCCTCTTCT
:||: |||||||
TACTTGTGCAAGACGAGGAGAAGT</td><td>5.43E-2</td></tr>
<tr><td>277. bta-miR-1301 MIMAT0009958</td><td>16161</td><td>-13.70</td><td>AGAAAGCCTCTT-CTTTGCTGCAA
|||:| |||||||
CTTTAGTGAGGATCCGTCGACGTT</td><td>5.43E-2</td></tr>
<tr><td>278. bta-miR-1298 MIMAT0009970</td><td>17472</td><td>-9.00</td><td>AATAAGCTTAAGGCTAAGAATGAA
:||: |||||||
ATGTAGACCTGTCGG--CTTACTT</td><td>3.66E-1</td></tr>
<tr><td>279. bta-miR-12052 MIMAT0046759</td><td>18190</td><td>-10.90</td><td>ATGCCACGCGTGATAGCATTGGG
:|| |||||||
AGAGTCAAACTTTAGAGTAACCC</td><td>2.5E-1</td></tr>
<tr><td>280. bta-miR-1301 MIMAT0009958</td><td>20578</td><td>-16.40</td><td>TCTATC-TTCGTTTGCAAGCTGCAT
||| :|| | ||| ||||||
CTTTAGTGAGGATCCGT-CGACGTT</td><td>2.42E-1</td></tr>
</table>

<table>
<tr><th>1. miR Name</th><th>leftmost position of predicted target site</th><th>folding energy (in - Kcal/mol)</th><th>heteroduplex</th><th>p value</th></tr>
<tr><td>281. bta-miR-33b
MIMAT0009295</td><td>21108</td><td>-7.30</td><td>ACAGAGTTTTCTTGGAATGCAG
 ||: |: | | ||||||
CGTTACG-TTG-TCGTTACGTG</td><td>3.16E-1</td></tr>
<tr><td>282. bta-miR-11986
MIMAT0046385</td><td>21872</td><td>-14.20</td><td>CAGGA--AATGGATTGGACAT
 |||| ||: ||||||||
TTCCTGATTGAGTAACCTGTT</td><td>2.65E-1</td></tr>
<tr><td>283. bta-miR-1298
MIMAT0009970</td><td>22168</td><td>-5.94</td><td>GTGATCT--TCCCGAGAATGAT
   |||| ||||||
ATGTAGACCTGTCGGCTTACTT</td><td>3.85E-1</td></tr>
<tr><td>284. bta-miR-33b
MIMAT0009295</td><td>22674</td><td>-11.10</td><td>ATCATGC-CTTTAAATGCAC
   |||| |||||||
CGTTACGTTGTCGTTACGTG</td><td>1.24E-1</td></tr>
<tr><td>285. bta-miR-2475
MIMAT0012066</td><td>22677</td><td>-11.23</td><td>ATGCCTTTAAATGCAC-CACTTCT
 :|| ||| ||||||
GGCG-ACGTCAAAGTGTGTGAAGT</td><td>1.24E-1</td></tr>
<tr><td>286. bta-miR-2285al-5p
MIMAT0040949</td><td>22947</td><td>-7.30</td><td>AGTATATCGTAC-CACTTTGT
      :|| || |||||||
ATTTTGGGCTTGTTTGAAACA</td><td>2.39E-1</td></tr>
<tr><td>287. bta-miR-6529a
MIMAT0025565</td><td>25625</td><td>-6.09</td><td>ATTCT--AATGGTAATCTCTA
|:||| ||||||
TGAGACGCGGAGACTAGAGAG</td><td>2.65E-1</td></tr>
<tr><td>288. bta-miR-2284u
MIMAT0011895</td><td>25657</td><td>-8.00</td><td>TACTTAACAAACAGAACTTTT
     ||| ||||||||
TCTTTTTGGCTTGCTTGAAAA</td><td>7.54E-2</td></tr>
<tr><td>289. bta-miR-10178-5p
MIMAT0040933</td><td>27467</td><td>-20.40</td><td>AAATG-AATAGGTTACAGGAGG
   || :|||| |||||||
ACGACGGAGTCCAGGGTCCTCC</td><td>2.14E-1</td></tr>
<tr><td>290. bta-miR-10178-5p
MIMAT0040933</td><td>27681</td><td>-13.69</td><td>TGATTATACTGGACACCAGGAGT
|| || |||||||
ACGACGGAGTCC-AGGGTCCTCC</td><td>2.21E-1</td></tr>
<tr><td>291. bta-miR-2284u
MIMAT0011895</td><td>28048</td><td>-8.60</td><td>AGGTAGACCTTAT-AACTTTA
||: |:||| |: ||||||
TCTTTTTGGCTTGCTTGAAAA</td><td>2.5E-1</td></tr>
<tr><td>292. bta-miR-2284u
MIMAT0011895</td><td>29371</td><td>-5.74</td><td>TGAGAAATAATATCTAAACTTTA
 ||:|||: ||||||
TCTTTTTG--GCTTGCTTGAAAA</td><td>3.85E-1</td></tr>
<tr><td>293. bta-miR-10178-5p
MIMAT0040933</td><td>29540</td><td>-19.30</td><td>TGCTACTTCTCAGCTACCATCAGGAGG
|||| :||||| || ||||||
ACGA--CGGAGTC-CAGG--GTCCTCC</td><td>7.91E-3</td></tr>
<tr><td>294. bta-miR-196a
MIMAT0009255</td><td>30051</td><td>-18.90</td><td>TCTGGCAACA-GAACCCCTACCTC
:| :||||| ||| ||||||
GGGTTGTTGTACTT--TGATGGAT</td><td>2.94E-1</td></tr>
</table>

1. miR Name	leftmost position of predicted target site	folding energy (in -Kcal/mol)	heteroduplex	p value
295. bta-miR-33b MIMAT0009295	30220	-12.50	GC--CCCAATAAACAATGCAC<br>|| |||:| |||||||| CGTTACGTTGT-CGTTACGTG	5.91E-3
296. bta-miR-106a MIMAT0003784	30469	-7.60	GATTTG-ACAGTA--CACTTTC<br>|::|| || ||| |||||| ATGGACGTGACATTCGTGAAAA	3.29E-1
297. bta-miR-411c-5p MIMAT0025545	206	-9.60	TCACAATGT-CGAAGATCAACA<br>|||| | ||||||| TTACATACAAGAGACTAGTTGG	8.01E-2
298. bta-miR-10179-5p MIMAT0040937	1123	-13.40	CGTTATGTTATGAGACTGCAGA<br>:||: | ||||||| GGTGTCGGATGGTGGGACGTCT	1.69E-1
299. bta-miR-2285a MIMAT0011797	3429	-9.10	TTGCTGGTTGCGATCAGTTTTG<br>|: |||||||| GGTTCTTAAAGTAAGTCAAAAA	1.22E-2
300. bta-miR-2449 MIMAT0012027	3454	-15.20	GTGATGC-AGAAACTGCCCT<br>|: ||| |||||| CGGGACGACAGAGGACGGGT	1.22E-2
301. bta-miR-299 MIMAT0009274	6138	-14.20	TTGTATTACTTTTGGTAAACCT<br>||||| :|||||||| TACATACACCCTGCCATTTGGT	2.14E-1
302. bta-miR-142-5p MIMAT0003790	8803	-8.00	CATGTGTT--GCACTTTATT<br>|||:| ||||||| CATCACGAAAGATGAAATAC	1.54E-1
303. bta-miR-130a MIMAT0009223	8809	-13.50	TTGCACTTTATTACACATGCACTT<br>||| |||| ||| |||||| TACGGGAAA-ATTGT-AACGTGAC	1.54E-1
304. bta-miR-454 MIMAT0009326	8813	-10.00	ACTTTAT-TACA--CATGCACTT<br>||::||| || |||||| TGGGATATTCGTTATAACGTGAT	1.54E-1
305. bta-miR-130a MIMAT0009223	8905	-15.00	GTGCTTTCCTCTGC-TTGCACTA<br>:|||::| :| ||||||| TACGGGA-AAATTGTAACGTGAC	2.69E-1
306. bta-miR-142-5p MIMAT0003790	9497	-5.20	TTTATGC--CACGCTTTATT<br>||| :|||||| CATCACGAAAGATGAAATAC	3.48E-1
307. bta-miR-2287 MIMAT0011794	10048	-16.30	GATAAGGTCTACT-GTCCCAG<br>|:||: ||||||| TTCACGACGGTGGTCAGGGTC	1.75E-1
308. bta-miR-107 MIMAT0003785	10560	-18.50	TGTAGC-ATG-GCTTTATGCTGCT<br>:|||| || :| |||||||| CTATCGGGACATG--TTACGACGA	8.51E-2

<table>
<tr><th>1. miR Name</th><th>leftmost position of predicted target site</th><th>folding energy (in -Kcal/mol)</th><th>heteroduplex</th><th>p value</th></tr>
<tr><td>309. bta-miR-411c-5p
MIMAT0025545</td><td>12065</td><td>-12.70</td><td>CTAGTG-GTTCTGCTAATCAACA
   ||: ||||| || ||||||
TTACATACAAGA-GACTAGTTGG</td><td>3.46E-2</td></tr>
<tr><td>310. bta-miR-2488
MIMAT0012084</td><td>12067</td><td>-7.26</td><td>AGTGGTTCTGCTAATCAACAG
|| || |||||||
TCTCC-CTTATCCTAGTTGTA</td><td>3.46E-2</td></tr>
<tr><td>311. bta-miR-129-5p
MIMAT0009221</td><td>12714</td><td>-5.50</td><td>TGATGGTCTTAAGTATACAAAAAT
    :|:|: || ||||||
TCGTTCGGG--TCTGGCGTTTTTC</td><td>3.34E-1</td></tr>
<tr><td>312. bta-miR-107
MIMAT0003785</td><td>14412</td><td>-15.40</td><td>GACTTGCTTT-TATA-TGCTGCT
|| ||::| ||:| |||||||
CT-ATCGGGACATGTTACGACGA</td><td>7.54E-2</td></tr>
<tr><td>313. bta-miR-133c
MIMAT0029949</td><td>15916</td><td>-6.00</td><td>ACCACGAAAATGAAGAATACCAAAA
      |||||| || ||||||
CGACCATTTTAC--CT--TGGTTTA</td><td>2.69E-2</td></tr>
<tr><td>314. bta-miR-485
MIMAT0009328</td><td>16150</td><td>-16.50</td><td>GCAGTTGCATCA--GAAAGCCTCT
  |:|| ||||| | |||||||
GCTTAA-GTAGTGCCGGTCGGAGA</td><td>5.43E-2</td></tr>
<tr><td>315. bta-miR-107
MIMAT0003785</td><td>16162</td><td>-17.80</td><td>GAAAGCCT-CTTCTTTGCTGCA
|| ||||: | ||||||
CTATCGGGACATGTTACGACGA</td><td>5.43E-2</td></tr>
<tr><td>316. bta-miR-133c
MIMAT0029949</td><td>16264</td><td>-11.60</td><td>GTGATGTAAATGATGTTACCAAAT
|: |||| :||| |||||||
CG-ACCATT--TTACCTTGGTTTA</td><td>1.36E-1</td></tr>
<tr><td>317. bta-miR-411c-5p
MIMAT0025545</td><td>16890</td><td>-9.80</td><td>AATGT-TGTGAACT-ATCAACA
||||| ||| || ||||||
TTACATACAAGAGACTAGTTGG</td><td>2.82E-1</td></tr>
<tr><td>318. bta-miR-3065
MIMAT0046741</td><td>18240</td><td>-16.10</td><td>TCCA-CAGGAATTGATTTTGTTGT
|||| || | |||||||||||
AGGTCGT-AGTCACTAAAACAACT</td><td>2.14E-1</td></tr>
<tr><td>319. bta-miR-152
MIMAT0009238</td><td>20418</td><td>-12.80</td><td>AGTAAG-AGTGTT-TGCACTGT
  :||| |||: |||||||
GGGTTCAAGACAGTACGTGACT</td><td>2.21E-1</td></tr>
<tr><td>320. bta-miR-411c-5p
MIMAT0025545</td><td>21967</td><td>-9.00</td><td>ATTATAAAATCACT-ATCAACC
         || || |||||||
TTACATACAAGAGACTAGTTGG</td><td>9.19E-2</td></tr>
<tr><td>321. bta-miR-11971
MIMAT0046336</td><td>21973</td><td>-19.80</td><td>AAATCACTATCAACCCCTCA
   ||||| || |||||||
AAGAGTGAGAGACGGGGAGT</td><td>9.19E-2</td></tr>
<tr><td>322. bta-miR-11983
MIMAT0046376</td><td>23060</td><td>-13.10</td><td>TCTACTGAAACCATTACCACTGGTT
:|:|| || |||||||
GGGTG--TCCGG--AGCGTGACCAG</td><td>7.42E-2</td></tr>
</table>

<table>
<tr><th>1. miR Name</th><th>leftmost position of predicted target site</th><th>folding energy (in - Kcal/mol)</th><th>heteroduplex</th><th>p value</th></tr>
<tr><td>323. bta-miR-216a MIMAT0009265</td><td>28112</td><td>-10.50</td><td>GGACATCTGGAGACCTGAGATTA
||| || |||||||||
AGTGTCAAC-GGTCGACTCTAAT</td><td>3.03E-1</td></tr>
<tr><td>324. bta-miR-142-5p MIMAT0003790</td><td>28272</td><td>-5.40</td><td>GCTACACTCACT-CTTTATG
|| || |||||||
CATCACGAAAGATGAAATAC</td><td>6.2E-2</td></tr>
<tr><td>325. bta-miR-411c-5p MIMAT0025545</td><td>29316</td><td>-8.80</td><td>GGTAATTACCGACTGCCATCAACC
||| |||||||
TTACATACAAGAGAC--TAGTTGG</td><td>3.48E-1</td></tr>
<tr><td>326. bta-miR-129-5p MIMAT0009221</td><td>29599</td><td>-10.10</td><td>GAATTACTCAG-TTTCAAAAAG
|:||| : |||||||
TCGTTCGGGTCTGGCGTTTTTC</td><td>8.64E-2</td></tr>
<tr><td>327. bta-miR-2287 MIMAT0011794</td><td>29657</td><td>-20.30</td><td>TATTGC-ACCAGGAGTCCCAG
||| ||| ||||||||
TTCACGACGGTGGTCAGGGTC</td><td>2.69E-1</td></tr>
<tr><td>328. bta-miR-2345 MIMAT0011880</td><td>29690</td><td>-10.60</td><td>GGGGTACTGGTACAGACACAAC
| :|| |: | |||||||
CTTTAT-TCT-TTTGTGTGTTG</td><td>2.01E-1</td></tr>
<tr><td>329. bta-miR-107 MIMAT0003785</td><td>29733</td><td>-17.90</td><td>GATGGCAACCAGCGT-CAACTGCTGCC
|||:|| || || ||| ||||||
CTATCG--GG--ACATGTT-ACGACGA</td><td>1.11E-1</td></tr>
<tr><td>330. bta-miR-130a MIMAT0009223</td><td>30218</td><td>-19.80</td><td>GAGCCCCAATAAACAATGCACTG
|||| |||| |||||||
TACGGG-AAAATTGTAACGTGAC</td><td>5.91E-3</td></tr>
<tr><td>331. bta-miR-152 MIMAT0009238</td><td>30222</td><td>-16.44</td><td>CCCAA--TAAACAATGCACTGT
||||| ||||||||
GGGTTCAAGACAGTACGTGACT</td><td>5.91E-3</td></tr>
<tr><td>332. bta-miR-2488 MIMAT0012084</td><td>30522</td><td>-7.90</td><td>AATTTGAATGCATATCAACAA
||||: |||||||
TCTCCCTTAT-CCTAGTTGTA</td><td>2.11E-1</td></tr>
<tr><td>333. bta-miR-2284e MIMAT0012080</td><td>222</td><td>-8.60</td><td>CAACAAATACGGTCTCGAACTA
|||| ||: ||||||
CCTTTTTAGGCT--TGCTTGAA</td><td>8.01E-2</td></tr>
<tr><td>334. bta-miR-2285bi MIMAT0046389</td><td>233</td><td>-14.50</td><td>GTCTCGAACTACA-CTGGGCTC
|:| :|| || |||||||
CGGTTTTTCAAGTAGACCCGAA</td><td>8.01E-2</td></tr>
<tr><td>335. bta-miR-2285y MIMAT0029944</td><td>2214</td><td>-11.90</td><td>TCTTGCATTTGCATGGGTTTT
|||| ||||||||
TTTTTTCAAACAAACCCAAAA</td><td>2.61E-1</td></tr>
<tr><td>336. bta-miR-2284ab MIMAT0030437</td><td>5797</td><td>-13.00</td><td>AAAAATTTGAAACAAACTTTTA
|||||:: | ||||||||||
TTTTTGGGTT-GGTTTGAAAAT</td><td>2.39E-2</td></tr>
</table>

1. miR Name	leftmost position of predicted target site	folding energy (in -Kcal/mol)	heteroduplex	p value
337. bta-miR-2284ab MIMAT0030437	7546	-9.10	TATAAACCAGGTAATACTTTTA<br>|||: :|| ||||||| TTTTTGGGTTGGTT-TGAAAAT	2.94E-1
338. bta-miR-2463 MIMAT0012050	8808	-13.90	GTTGCACTTTATTACACATGCAC<br>:|| ||: |||||||| GTCTGTAAAG-GTGGTGTACGTC	1.54E-1
339. bta-miR-940 MIMAT0009386	9261	-12.10	TAAAGGT-TTAGCACAGCCTG<br>:|: || |||||| CGCCTCGCCCCCGGGTCGGAA	2.28E-1
340. bta-miR-2284ab MIMAT0030437	9693	-8.30	AATGGCTCTTACT--ACTTTTA<br>::|:| ||: ||||||| TTTTTGGG-TTGGTTTGAAAAT	1.98E-1
341. bta-miR-10020 MIMAT0040419	14821	-10.50	TTAATAAATTTGGAAAAGCCAG<br>|: :| |||||| GCGGTACAAGGTTTCTTCGGTG	6.08E-3
342. bta-miR-2463 MIMAT0012050	15413	-7.30	TGTATGTGCTTTAATGTCATGCAA<br>|::| |::| |||||| GTCTGTA-AAGGT-GGTGTACGTC	7.31E-2
343. bta-miR-10020 MIMAT0040419	16602	-12.84	GAGATTGGAAAAGTGAAGCCAC<br>|| |||||||| GCGGTACAAGGTTTCTTCGGTG	3.48E-1
344. bta-miR-2428 MIMAT0011998	17010	-18.80	GTATACACTGCG-GCCAGCCA<br>| |:||: ||||||| TGGAGGCGGCGTGAGGTCGGT	3.2E-1
345. bta-miR-2463 MIMAT0012050	17016	-16.90	ACTGCG--GCCAGC-CATGCAG<br>:|: ||| | ||||||| GTCTGTAAAGGTGGTGTACGTC	3.2E-1
346. bta-miR-30f MIMAT0009282	17149	-8.20	CCACTCGTAAGTA-TGTGTTTACT<br>||| |||||||| TCGACTCTCACATCCCACAAATGT	3.39E-1
347. bta-miR-12038 MIMAT0046733	17730	-15.20	ATATATTCACAG--ACTGCAGA<br>:|:| |||| ||||||| TCCGTGACTGTCGAAGACGTCT	1.09E-1
348. bta-miR-2463 MIMAT0012050	19147	-11.40	TGTATGTTAACA-AACATGCAT<br>|::|| || ||||||| GTCTGTAAAGGTGGTGTACGTC	3.16E-1
349. bta-miR-329b MIMAT0009289	20107	-13.00	GTAATGATGCTCTGGCAACCTCC<br>|| :|:| |: |||||| TTTGTCTTTGGGTCTTTTGGAGA	2.82E-1
350. bta-miR-760-5p MIMAT0009372	21548	-9.54	TCCACTTACCCAGTTTGAGGGT<br>||: :|||||| GCCCGAGACCACCTGACTCCCC	1.45E-1

1. miR Name	leftmost position of predicted target site	folding energy (in - Kcal/mol)	heteroduplex	p value
351. bta-miR-940 MIMAT0009386	21657	-13.70	GCTATGCTGGATATTCAGCCTG<br>|| || ::|||||| CGCCTCGCCC-CCGGGTCGGAA	2.25E-1
352. bta-miR-2285cc MIMAT0046652	25627	-7.60	TCTAATGGTAATCTCTATGGTTTTA<br>|| ||| || |||||| AGTTTTCCA--AGTAAGTCCAAAAA	2.65E-1
353. bta-miR-2285n MIMAT0025531	25628	-7.00	CTAATGGTAATCTCTATGGTTTTA<br>|:|| :|| || |||||| GGTTTTCA--AGTAAGCCCAAAAA	2.65E-1
354. bta-miR-11994 MIMAT0046647	26803	-8.40	AACTTATTGCA-ACAACTCTCT<br>:|| ||||||| GTAGGTCTTGTCCCTTGAGAGT	3.34E-1
355. bta-miR-12038 MIMAT0046733	30660	-12.50	AAGAGTAGAGAGTTGACTGCAGA<br>||:| ||||||| TCCGTGACTGTCGA-AGACGTCT	1.28E-1
356. bta-miR-940 MIMAT0009386	30671	-16.10	GTTGACTGCAGAGGACATCAGCCTT<br>|: || || | || :||||||| CGCCT--CGCCCCC--GGGTCGGAA	1.28E-1
357. bta-miR-3154 MIMAT0025529	30675	-15.60	ACTGCAGAGGACATCAGCCTTCTT<br>|||| ||| || |||||| AGACG-AGGCTG-AG-GGGAAGAC	1.28E-1
358. bta-miR-2440 MIMAT0012015	2860	-9.90	AATACTATTTTAAACACTGCA<br>|:|:| ||||||| AGGTCCCAGAGT-AGTGACGT	1.36E-1
359. bta-miR-7859 MIMAT0030434	3429	-11.10	TTGCTGGTTGCGATCAGTTTTG<br>|| :||||||| AATGTACTTCGACGGTCAAAAA	1.22E-2
360. bta-miR-11986b MIMAT0046621	3639	-5.90	GCAGTGTGTTGCATACTGGAAAT<br>|| : :| :| |||||| TTTCCTG-ATTGAGTAACCTTTT	3.34E-1
361. bta-miR-10174-3p MIMAT0040928	3653	-12.70	ACTGGAAATGC-ATTAAATGTGAT<br>||| ||| | |||||||| GCACCATTAGGGACCGTTACACTA	3.34E-1
362. bta-miR-22-3p MIMAT0012536	3840	-12.40	TAGTGTGTACAA--AGCAGCTT<br>:||| ||| ||||||| GTCA-AGAAGTTGACCGTCGAA	1.11E-1
363. bta-miR-7859 MIMAT0030434	4808	-11.00	ATATATGCAGCCAGGATAAAGTTTTT<br>||:||| ||| | ||||||| AATGTACTTCG--AC--GGTCAAAAA	3.16E-1
364. bta-miR-186 MIMAT0003818	5091	-8.30	GGCT-GTAAG--AAGTTCTTTT<br>:||: ||| |:|||||| TCGGGTTTTCCTCTTAAGAAAC	1.95E-1

1. miR Name	leftmost position of predicted target site	folding energy (in -Kcal/mol)	heteroduplex	p value
365. bta-miR-2285cw MIMAT0046702	5795	-11.70	TTAAAAATTTGAAACAAACTTTT<br>|||||::|||| ||||||| TCTTTTTGGACTT-GATTGAAAA	2.39E-2
366. bta-miR-2284l MIMAT0011824	5797	-12.00	AAAAATTTGAAACAAACTTTT<br>|||||:::||| ||||||| TTTTTGGGCTT-GGTTGAAAA	2.39E-2
367. bta-miR-155 MIMAT0009241	6793	-8.10	GTTGCTA--GAGGTGCTTGCATTAT<br>:: ||| |:| |||||| TGGGGATAGTGCTA--ATCGTAATT	3.52E-1
368. bta-miR-2377 MIMAT0011923	8254	-13.90	TCTGCTAAG-CATGTGCAGG<br>||: || ||||||| TCTCGGATCCAGACACGTCA	3.07E-1
369. bta-miR-7859 MIMAT0030434	8449	-12.20	CTTAAAGGGGGTGC-AGTTTTT<br>|::| ||| ||||||| AATGTACTTCGACGGTCAAAAA	3.29E-1
370. bta-miR-302b MIMAT0009280	8794	-10.30	TATGGTTACCATGTGTTGCACTTT<br>|||| | |||||| GATGATTTTGTAC-CTTCGTGAAT	1.54E-1
371. bta-miR-2377 MIMAT0011923	8833	-14.40	TCTGCTGATGG--AGTGCAGT<br>||: || ||||||| TCTCGG-ATCCAGACACGTCA	1.54E-1
372. bta-miR-494 MIMAT0009334	9597	-10.30	ATATATATCTGTTGT-TGTTTCA<br>:|:|| || ||||||| CTCCAAAGGGCA-CATACAAAGT	6.88E-2
373. bta-miR-186 MIMAT0003818	9732	-11.20	TTGTAAGCTTAAGAATTCTTTG<br>||||||||||| TCGGGTTTTCCTCTTAAGAAAC	2.82E-1
374. bta-miR-562 MIMAT0009349	10068	-8.10	ACATGTGATATGTTCTGCTTC<br>:|| ||: |||||| CATTTACCATG-TCGACGAAA	1.75E-1
375. bta-miR-2284ac MIMAT0030447	11758	-9.00	CTTAATTGCT-TGC-AACATTT<br>|| ||: || ||||||| CCTTTTTCGGTTCGCTTGTAAA	3.25E-1
376. bta-miR-302b MIMAT0009280	11800	-9.90	TTGTGGCAATATTGTAGCACTTT<br>||:|| | ||||||| GATGATTTTGTACCTTCGTGAAT	6.2E-2
377. bta-miR-2459 MIMAT0012044	11834	-10.30	TACTTGCCACTTCTGATCTGGG<br>|| |: | |||||| TTAGTCGAGGG-TAGTAGACCG	6.2E-2
378. bta-miR-429 MIMAT0009315	11918	-14.70	ATAGCAAGTGCCTGACTAGTATTG<br>||| |:|| ||| |||||| TGCCGT-AATGGTCTG-TCATAAT	1.75E-1

1. miR Name	leftmost position of predicted target site	folding energy (in -Kcal/mol)	heteroduplex	p value
379. bta-miR-656 MIMAT0009361	11993	-6.80	AGAG--TGAATTTGT-TAATATG<br>|||| ||| ||| |||||| TCTCCAACT--GACATATTATAA	2.39E-1
380. bta-miR-562 MIMAT0009349	12113	-8.40	GTAATATTGCTAAATCTGCTTA<br>|||| || || |||||| CATT--TACCATGTCGACGAAA	3.46E-2
381. bta-miR-2917 MIMAT0012885	13304	-9.60	ACACTACTGTTCAGTCAA<br>|:| ||| |||||| TCAGGT-ACA-GTCAGTA	3.12E-1
382. bta-miR-155 MIMAT0009241	14842	-18.60	GGCTCTATTATGA--AGCATTAT<br>: |:||||:|:|| ||||||| TGGGGATAGTGCTAATCGTAATT	6.08E-3
383. bta-miR-190a MIMAT0009251	15778	-6.90	ACGATGTCTATCTTCCATATCC<br>|||| |||||| TGGATTATATAGTTTGTATAGT	2.69E-1
384. bta-miR-186 MIMAT0003818	16155	-9.20	TGCATCAGAAAGCCTCTTCTTTG<br>|| :||:|| | ||||||| TCG-GGTTTTCCTCTTAAGAAAC	5.43E-2
385. bta-miR-10166-5p MIMAT0040913	16157	-10.29	CATCAGAAAGCCTTCTTTGCTGCAA<br>|| ||| || |||||| GT-GTC--AGACACTAG--TCGACGTG	5.43E-2
386. bta-miR-494 MIMAT0009334	16217	-11.00	TAAATATGTTTTGAGTGTTTCA<br>|| :||||||| CTCCAAAGGGCACATACAAAGT	1.69E-1
387. bta-miR-10174-3p MIMAT0040928	16245	-10.40	GTGTGTAACGCACCAGG--ATGTGAT<br>|||| || || ||||||| GCACCATT--AGGGACCGTTACACTA	1.36E-1
388. bta-miR-155 MIMAT0009241	17834	-9.00	GCAGTTGTT-TGA--AGCATTAC<br>:| :|:|: :|| ||||||| TGGGGATAGTGCTAATCGTAATT	2.98E-1
389. bta-miR-190a MIMAT0009251	19601	-7.40	TATTAATA-ATACAACATATCC<br>:||||| |||||||| TGGATTATATAGTTTGTATAGT	5.58E-3
390. bta-miR-574 MIMAT0024577	20365	-24.00	CATACGACTC-CA-GCATACACTCT<br>||:|| |||| || :||:|||||| GTGTG-TGAGTGTGTGTGTGTGAGT	1.89E-1
391. bta-miR-24 MIMAT0009250	20831	-16.30	ACCGGGTTCTGCAGTTC-TTAGGCAG<br>|| |:| |||:|| |||||| TG-ACTA--TAGTCGAGTCATCCGTG	2.78E-1
392. bta-miR-2486-5p MIMAT0012081	21585	-9.10	AAAGGTTTACAATTTCAACTTCTCG<br>: ::|||| |||||| GGGTGGGATGT--GGCACGAAGAGG	1.34E-1

1. miR Name	leftmost position of predicted target site	folding energy (in - Kcal/mol)	heteroduplex	p value
393. bta-miR-494 MIMAT0009334	24196	-11.10	TGGGATACAGGTGT-TGTTTCC<br>|||| |||||| CTCCAAAGGGCACATACAAAGT	3.8E-1
394. bta-miR-10174-3p MIMAT0040928	24226	-11.40	AAGCGTAATTTCACATATGATGTGAA<br>||||| | :|||||| GCACCATTAGGG--ACCGTTACACTA	1.36E-1
395. bta-miR-2285bo MIMAT0046627	24684	-9.10	GGAAC--GTAAGACCTTTTCA<br>::||| || ||||||| TTTTGAGCTTGCTTGAAAAGT	2.18E-1
396. bta-miR-656 MIMAT0009361	24750	-5.00	CTCATTTACTTGTAATAATATT<br>:|||| ||||||| TCTCCAACTGACAT-ATTATAA	1.75E-1
397. bta-miR-494 MIMAT0009334	24933	-9.40	TAATTTACCTGCTGCTAATGTTTCT<br>||:| || ||||||| CTCCAAAGGGC-AC--ATACAAAGT	9.48E-2
398. bta-miR-2440 MIMAT0012015	26508	-10.70	CAAAGTGTTGCCTCCACTGCT<br>|| ||: | |||||| AGGTCCCAG-AGTAGTGACGT	2.42E-2
399. bta-miR-2377 MIMAT0011923	26971	-9.30	CTAGTAAAATTTAGTGCAGC<br>||: |: |||||| TCTCGGATCCAGACACGTCA	3.43E-1
400. bta-miR-365-5p MIMAT0012539	27139	-14.44	GTCA-CTGCGAAGGTTAGTCCCG<br>|| |||| |||||| GTGTAGACGGGGGTTTTCAGGGA	2.14E-1
401. bta-miR-2284ac MIMAT0030447	27324	-9.50	GCCGGATGTAATGCTGAACATTT<br>|: || |||||||| CCTTTTTCGGTTCG-CTTGTAAA	6.88E-2
402. bta-miR-10174-3p MIMAT0040928	27764	-12.10	ATATTACATCCCTTCAATCATGTGAA<br>|||||| |||||| GCACCATTAGGGA--CCGTTACACTA	2.11E-1
403. bta-miR-2285cw MIMAT0046702	28048	-11.30	AGGTAGACCT--TATAACTTTA<br>||: |:|||| ||||||| TCTTTTTGGACTTGATTGAAAA	2.5E-1
404. bta-miR-2284l MIMAT0011824	28050	-9.10	GTAGACCT--TATAACTTTA<br>|:|||: :|||||| TTTTTGGGCTTGGTTGAAAA	2.5E-1
405. bta-miR-155 MIMAT0009241	28053	-12.50	GACCTTATAACTTTAAGCATTAT<br>||:||| || | ||||||| TGGGGATAGTGCTAATCGTAATT	2.5E-1
406. bta-miR-2459 MIMAT0012044	28102	-11.90	CCCTAGTAATGGACATCTGGA<br>:||: : : ||||||| TTAGTCGAGGGTAGTAGACCG	3.03E-1

<table>
<tr><th>1. miR Name</th><th>leftmost position of predicted target site</th><th>folding energy (in -Kcal/mol)</th><th>heteroduplex</th><th>p value</th></tr>
<tr><td>407. bta-miR-2284ac MIMAT0030447</td><td>28673</td><td>-5.30</td><td>ACGTTTAGTTAATCCAAACATTA
||::|| | ||||||
CCTTTTTCGGTT-CGCTTGTAAA</td><td>1.64E-1</td></tr>
<tr><td>408. bta-miR-10174-3p MIMAT0040928</td><td>29196</td><td>-8.40</td><td>TCAGATTTGCC--AGCTTATGTGAC
|| || ||||||
GCACCATTAGGGACCG-TTACACTA</td><td>2.11E-1</td></tr>
<tr><td>409. bta-miR-656 MIMAT0009361</td><td>29365</td><td>-5.70</td><td>CATTGTTGA-GAAATAATATC
||||| |||||||
TCTCCAACTGACATATTATAA</td><td>3.85E-1</td></tr>
<tr><td>410. bta-miR-2486-5p MIMAT0012081</td><td>29529</td><td>-15.60</td><td>CCCA-AGCAAACTGCTACTTCTCA
|||| ||:| ||||||
GGGTGGGATGTGGC-ACGAAGAGG</td><td>7.91E-3</td></tr>
<tr><td>411. bta-miR-365-5p MIMAT0012539</td><td>29654</td><td>-22.20</td><td>GCCTATTGC-ACCAGGAGTCCCA
:||| |||::||||||
GTGTAGACGGGGGTTTTCAGGGA</td><td>2.69E-1</td></tr>
<tr><td>412. bta-miR-8549 MIMAT0037488</td><td>30056</td><td>-14.70</td><td>CAACAGAACC-CCTACCTC
|| ||| |||||||
TATG--GTGGATGATGGAG</td><td>2.94E-1</td></tr>
<tr><td>413. bta-miR-2377 MIMAT0011923</td><td>30383</td><td>-14.44</td><td>AGAGTTGGCCAAAGTGCAGA
||||:: ||||||
TCTCGGATCCAGACACGTCA</td><td>1.09E-1</td></tr>
<tr><td>414. bta-miR-656 MIMAT0009361</td><td>30606</td><td>-5.90</td><td>GGAG--AAAATG-ATAATATA
:||| || |||||||
TCTCCAACTGACATATTATAA</td><td>4.57E-2</td></tr>
<tr><td>415. bta-miR-211 MIMAT0009263</td><td>30937</td><td>-10.20</td><td>GCCGAACAAGTGCCCAAGGGAA
|:|| :|| | |||||||
CCGTTT-CCTACTGTTTCCCTT</td><td>6.29E-2</td></tr>
<tr><td>416. bta-miR-1179 MIMAT0009957</td><td>1309</td><td>-6.10</td><td>GATGCAGTTG--TAAATGCTT
:| || |||||||
GGTTGGTTACTTTCTTACGAA</td><td>3.57E-1</td></tr>
<tr><td>417. bta-miR-2464-5p MIMAT0012051</td><td>5103</td><td>-8.30</td><td>TTCTTTTAATTTTG-ATCAGAAGG
|::| |:|| ||||||
CAGAAGGTCCAGACGTCGTCTTCA</td><td>9.05E-2</td></tr>
<tr><td>418. bta-miR-2285cl MIMAT0046681</td><td>5797</td><td>-8.90</td><td>AAAAATTTGAAACAAACTTTT
|||||:::||| ||||||
TTTTTGGGCTT-ACTTGAAAT</td><td>2.39E-2</td></tr>
<tr><td>419. bta-miR-7691 MIMAT0029952</td><td>6273</td><td>-11.70</td><td>TGGT--GGTGATATCTCAGA
|:|| ||: ||||||
ATCAATAATCTGGAGAGTCG</td><td>3.52E-1</td></tr>
<tr><td>420. bta-miR-1179 MIMAT0009957</td><td>7708</td><td>-8.10</td><td>ACATATGATGATGTTAATGCTA
|| ::|||| ||||||
GGTTGGTTACT-TTCTTACGAA</td><td>2.18E-1</td></tr>
</table>

1. miR Name	leftmost position of predicted target site	folding energy (in -Kcal/mol)	heteroduplex	p value
421. bta-miR-2285cu MIMAT0046698	9615	-10.26	TTCAAATCATGCTTTTTGGGTAT<br>:|||| |:|||||| CGGTTT-TCCAAGCAGACCCATA	6.88E-2
422. bta-miR-12057 MIMAT0046764	10046	-13.00	GTGATAAGGTCTACTGTCCCAGA<br>||:|| |||||| CCGAGAGGAAGGTG--AGGGTCA	1.75E-1
423. bta-miR-677 MIMAT0012003	10162	-12.20	CGTCTAAGC--CTTA-CAGTGAT<br>|||| ||:| |||||| CAGTCTTCGACGAGTAGTCACTC	3.56E-2
424. bta-miR-503-5p MIMAT0025557	10562	-12.00	TAGCATGGCTTTATGCTGCTA<br>:|| |: ||: :||||||| GTCATG-ACAAGGGCGACGAT	8.51E-2
425. bta-miR-2372 MIMAT0011917	16121	-9.80	CTGCTC-TTCTCAAACATCATTA<br>:|:| ||: ||||||| ACTGGGTTAGG-AAAGTAGTAAG	5.43E-2
426. bta-miR-483 MIMAT0009327	17104	-13.90	CCAAGGTCAGG-GTGGAGTGC<br>||| | |||||| TTCTGCCCTCCTCTCCTCACT	3.76E-1
427. bta-miR-2388-3p MIMAT0011941	17778	-13.60	TTCAATGTTGCTATTACTCGAGC<br>||| ||||||| AGTAGAGGTCGA--AGGAGCTCG	2.86E-1
428. bta-miR-323 MIMAT0009284	19038	-11.30	ATGTTTTGGAACTGTAATGTGG<br>|:|: ||||||||| TCTCCAGCTGGCACATTACACG	2.42E-1
429. bta-miR-2285br MIMAT0046636	19389	-11.11	ACAG-CTACTACAGCAGGTTTT<br>|||| |||||||| TGTCTTTCAAGTAAGTCCAAAA	3.12E-1
430. bta-miR-1814c MIMAT0011984	19876	-10.60	TTTACATTTCCACGACAAAAG<br>||| :||| :|||||| TTTTGTTTGGGT-TTGTTTTG	3.48E-1
431. bta-miR-2464-5p MIMAT0012051	20262	-8.30	GTTT--ATGGTAATTTCAACCAGAAGA<br>||:| ||| || |||||| CAGAAGGTCCA--GACGT-CGTCTTCA	1.95E-1
432. bta-miR-451 MIMAT0009323	21151	-10.50	ATTTTGCATTTTGG--ACGGTTT<br>||| ||||||| TTTGAGTCATTACCATTGCCAAA	2.01E-1
433. bta-miR-1179 MIMAT0009957	21172	-7.50	TCTGCACAAATG--CAAATGCTT<br>:| :| |||| ||||||| GGTTG--GTTACTTTCTTACGAA	2.01E-1
434. bta-miR-503-5p MIMAT0025557	24930	-16.70	TTATAATTTACCTGCTGCTA<br>||:||||||| GTCATGACAAGGGCGACGAT	9.48E-2

<table>
<tr><th>1. miR Name</th><th>leftmost position of predicted target site</th><th>folding energy (in -Kcal/mol)</th><th>heteroduplex</th><th>p value</th></tr>
<tr><td>435. bta-miR-2285cl MIMAT0046681</td><td>25659</td><td>-5.40</td><td>CTTAAC-AAACAGAACTTTT
   ||| |||||||
TTTTTGGGCTTACTTGAAAT</td><td>7.54E-2</td></tr>
<tr><td>436. bta-miR-2464-5p MIMAT0012051</td><td>26193</td><td>-8.50</td><td>TAATGCTA-TACT-CA-CAGAAGT
     |:| || || |||||||
CAGAAGGTCCAGACGTCGTCTTCA</td><td>4.38E-2</td></tr>
<tr><td>437. bta-miR-502a MIMAT0009338</td><td>27152</td><td>-17.50</td><td>TTAGTCCCGGTCT--GTGCATT
  |:||| |:|: |||||||
ACTTAGGAACGGGTCCACGTAA</td><td>2.14E-1</td></tr>
<tr><td>438. bta-miR-2285cl MIMAT0046681</td><td>28050</td><td>-8.10</td><td>GTAGACCT-TAT-AACTTTA
  |:|||: || |||||||
TTTTTGGGCTTACTTGAAAT</td><td>2.5E-1</td></tr>
<tr><td>439. bta-miR-7691 MIMAT0029952</td><td>28154</td><td>-9.90</td><td>CGGTT-TTA-ATGTCTCAGA
 :||| ||| |: ||||||
ATCAATAATCTGGAGAGTCG</td><td>3.71E-1</td></tr>
<tr><td>440. bta-miR-148c MIMAT0016933</td><td>29044</td><td>-5.10</td><td>TCAACCCAGAAACAAACAACTTGA
||| || ||| ||||||
AGTCACGT-ACTGT--CTTGAACC</td><td>3.15E-2</td></tr>
<tr><td>441. bta-miR-7691 MIMAT0029952</td><td>29534</td><td>-16.00</td><td>GCAAACTGCTACTTCTCAGC
          ||:|||||||
ATCAATAATCTGGAGAGTCG</td><td>7.91E-3</td></tr>
<tr><td>442. bta-miR-12057 MIMAT0046764</td><td>29660</td><td>-10.10</td><td>TGC--ACC-AGGAGTCCCAGC
 || || | ||||||
CCGAGAGGAAGGTGAGGGTCA</td><td>2.69E-1</td></tr>
<tr><td>443. bta-miR-12026 MIMAT0046719</td><td>29849</td><td>-18.50</td><td>CCAGGCTGATGTCAATACCCCGGC
|| |||: | || ||||||
GGACCGG--TC-GT-CAGGGGCCC</td><td>1.98E-1</td></tr>
<tr><td>444. bta-miR-2464-5p MIMAT0012051</td><td>30195</td><td>-6.40</td><td>TTGAATAAGCCCCGC--CAGAAGA
       || | || ||||||
CAGAAGGTCCAGACGTCGTCTTCA</td><td>5.91E-3</td></tr>
<tr><td>445. bta-miR-148c MIMAT0016933</td><td>30288</td><td>-8.50</td><td>GGAG-AAATGTTAAAACTTGG
  || ||| :| |||||||
AGTCACGTACTGTCTTGAACC</td><td>4.98E-2</td></tr>
<tr><td>446. bta-miR-2285ck MIMAT0046676</td><td>30373</td><td>-11.30</td><td>GATCAAGATTAGA-GTTGGCCA
          ||| |||||||
TGACTTTGTTTCTAAAACCGGT</td><td>1.09E-1</td></tr>
<tr><td>447. bta-miR-2439-3p MIMAT0012014</td><td>2546</td><td>-10.00</td><td>TAAAAGGTTCTG-GTTCTGATT
        :||| |||||||
AGACGGATGGACAGAAGACTAT</td><td>2.65E-2</td></tr>
<tr><td>448. bta-miR-34c MIMAT0003854</td><td>3448</td><td>-14.00</td><td>TTGGCAGTGATGCAGAAACTGCCC
    |||:   :| ||||||
GTTAGTCG-ATTG-ATGTGACGGA</td><td>1.22E-2</td></tr>
</table>

<table>
<tr><th>1. miR Name</th><th>leftmost position of predicted target site</th><th>folding energy (in -Kcal/mol)</th><th>heteroduplex</th><th>p value</th></tr>
<tr><td>449. bta-miR-495 MIMAT0009335</td><td>3523</td><td>-8.50</td><td>CAGTATAGT-CAGT-TGTTTGTTG
|| ||| || : ||||||||
TTC--TTCACGTGGTACAAACAAA</td><td>2.58E-1</td></tr>
<tr><td>450. bta-miR-2284a MIMAT0011906</td><td>5797</td><td>-8.50</td><td>AAAAATTTGAAACAAACTTTT
|||||::: ||| |||||||
TTTTTGGG-GTTGCTTGAAAA</td><td>2.39E-2</td></tr>
<tr><td>451. bta-miR-6528 MIMAT0025562</td><td>6174</td><td>-14.50</td><td>CCATGAGCAAGCTTCCCTCA
|| | ||||||||
GGAGGAAGGGAGAAGGGAGA</td><td>3.34E-1</td></tr>
<tr><td>452. bta-miR-2285dk MIMAT0046749</td><td>7190</td><td>-7.69</td><td>TGTTTGCTCTTATTAGTATTCAGA
||: :||||||
TCAACCGGTTTTACAAGTAAGTCC</td><td>2.98E-1</td></tr>
<tr><td>453. bta-miR-495 MIMAT0009335</td><td>7488</td><td>-11.40</td><td>TAATGGTGGCACTGGCTTTTGTTC
:||| |||:: ||||||
TTCTTCAC-GTGGT-ACAAACAAA</td><td>2.95E-2</td></tr>
<tr><td>454. bta-miR-2439-3p MIMAT0012014</td><td>7878</td><td>-7.84</td><td>AATGGTTGATAAAAATCTGATA
|| :|: |||||||
AGACGGATGGACAGAAGACTAT</td><td>3.48E-1</td></tr>
<tr><td>455. bta-miR-19a MIMAT0004336</td><td>8794</td><td>-8.20</td><td>TATGGTTACCATG-TGTTGCACT
|||: ||||||
AGTCAAAACGTATCTAAACGTGT</td><td>1.54E-1</td></tr>
<tr><td>456. bta-miR-302d MIMAT0009279</td><td>8796</td><td>-10.30</td><td>TGGTTACCATGTGTTGCACTTT
|||| | ||||||
TGATTTTGTAC-CTTCGTGAAT</td><td>1.54E-1</td></tr>
<tr><td>457. bta-miR-302d MIMAT0009279</td><td>8815</td><td>-7.70</td><td>TTTATTACA--CATGCACTTT
:|| ||| ||||||
TGATTTTGTACCTTCGTGAAT</td><td>1.54E-1</td></tr>
<tr><td>458. bta-miR-2370-5p MIMAT0011914</td><td>9499</td><td>-14.30</td><td>TATGCCACGCTTT--ATTTCCCT
:||| :|||| ||||||
TATTGGT-TGAAAGTAAAAGGGT</td><td>3.48E-1</td></tr>
<tr><td>459. bta-miR-2439-3p MIMAT0012014</td><td>10664</td><td>-9.10</td><td>TTAGCCAAGTT-AAGTCTGATC
||| | :| ||||||
AGACGGATGGACAGAAGACTAT</td><td>3.07E-1</td></tr>
<tr><td>460. bta-miR-2317 MIMAT0011838</td><td>11434</td><td>-12.70</td><td>GTGAATGTTTTGTATTTCACAGAT
|| |||| |||||||
ATAGTCCATAACA-GTAGTGTCTC</td><td>8.26E-2</td></tr>
<tr><td>461. bta-let-7g MIMAT0003838</td><td>11446</td><td>-12.90</td><td>TATTTCACAGAT--ATACCTCA
|||:|: |||||||
TTGACATGTTTGATGATGGAGT</td><td>8.26E-2</td></tr>
<tr><td>462. bta-miR-302d MIMAT0009279</td><td>11802</td><td>-9.90</td><td>GTGGCAATATTGTAGCACTTT
||:|| | |||||||
TGATTTTGTACCTTCGTGAAT</td><td>6.2E-2</td></tr>
</table>

1. miR Name	leftmost position of predicted target site	folding energy (in -Kcal/mol)	heteroduplex	p value
463. bta-miR-19a MIMAT0004336	11805	-12.50	GCAATATTGTAGCACTTTGCACA<br>|||:| |||||||| AGTCAAAACGTATCTAAACGTGT	6.2E-2
464. bta-miR-2439-3p MIMAT0012014	11832	-15.30	AATACTTGCC--ACTTCTGATC<br>|:|:|| |||||||| AGACGGATGGACAGAAGACTAT	6.2E-2
465. bta-miR-12027 MIMAT0046720	13168	-14.10	GGGTTGTGCAAATTACGCGGCA<br>|||: ||||||| TGTGGTCCCTTTGTCGCGCCGT	1.34E-1
466. bta-miR-2414 MIMAT0011977	13913	-10.00	AGGCTTAGTAGGCATTTTAACACT<br>::| :| |||: |||||| ATTGTGGTAGGCGTG--ATTGTGT	3.29E-1
467. bta-miR-495 MIMAT0009335	14308	-15.10	TGGATGGTGTGCC--TTTTGTTG<br>:|| :||| || |||||| TTCT-TCACGTGGTACAAACAAA	3.62E-1
468. bta-miR-2317 MIMAT0011838	17721	-9.84	GATTATGTTATATATTCACAGAC<br>||:| || ||||||| ATAGTCCATAACAGTAGTGTCTC	1.09E-1
469. bta-miR-2347 MIMAT0011882	18856	-15.10	TTAAAGCTGCCATGCTCTGCA<br>|||: | ||||||| CAAGTCGGAACAAGGAGACGT	6.11E-2
470. bta-miR-19a MIMAT0004336	20411	-9.10	TGGTGGTAGTAAGAGTGTTTGCACT<br>|:| || :||||||| AGTCAAAACGT-ATC-TAAACGTGT	2.21E-1
471. bta-miR-2285u MIMAT0031094	21192	-9.20	TCTTCTAGTGAAGGGTTTTTA<br>|| |||||||| TTTCAAGCA-AGCCCAAAAAG	2.01E-1
472. bta-miR-19a MIMAT0004336	21871	-10.80	CCAGGAAATGGATTGGACATTGCACC<br>||| || || :|| |||||| AGTC-AAAACGTA-TCT-AAACGTGT	2.65E-1
473. bta-miR-2285da MIMAT0046707	22028	-7.44	TGAAAAG--AAAGATGGTTTTT<br>:||||| |||||| TTTTTTCAAGTAAGTCCAAAAG	6.99E-2
474. bta-miR-660 MIMAT0004344	22107	-15.50	AAGCT-AGCTATGATTATGGGTT<br>|||| | |||| |||||| GTCGAGGCTATAC-GTTACCCAT	3.48E-1
475. bta-miR-1256 MIMAT0009960	23221	-10.80	TTCACGGTGGAA--CAATGCCA<br>| :||| ||||||| TAGATCTCTCTTCAGTTACGGA	1.89E-1
476. bta-miR-2439-3p MIMAT0012014	23230	-7.80	GAACAATGCCAGGCAGTCTGATA<br>|| | ||||||| AGACGGATGGAC-AGAAGACTAT	1.89E-1

<table>
<tr><th>1. miR Name</th><th>leftmost position of predicted target site</th><th>folding energy (in - Kcal/mol)</th><th>heteroduplex</th><th>p value</th></tr>
<tr><td>477. bta-miR-2450b MIMAT0012029</td><td>23608</td><td>-8.30</td><td>TAGGCTGCATGATGCTTAGACCATA
            |:|| ||||||
GTGTACGTGGAGATGA--CTGGTAC</td><td>3.29E-1</td></tr>
<tr><td>478. bta-miR-2285dk MIMAT0046749</td><td>24722</td><td>-10.50</td><td>GCAGCCTGATGTCTTTTATTCAGG
              ||:|||||||
TCAACCGGTTTTACAAGTAAGTCC</td><td>1.75E-1</td></tr>
<tr><td>479. bta-miR-2285u MIMAT0031094</td><td>25026</td><td>-5.30</td><td>TCAACCTGT-AGGTGTTTTTA
      :|| || ||||||
TTTCAAGCAAGCC-CAAAAAG</td><td>2.46E-1</td></tr>
<tr><td>480. bta-miR-2393 MIMAT0011946</td><td>25250</td><td>-5.60</td><td>ACCCCATTACATCTAAATCTA
        ||| |||||||
TTTTCTTTTGT-TTTTTAGAT</td><td>1.04E-1</td></tr>
<tr><td>481. bta-miR-2893 MIMAT0013851</td><td>26506</td><td>-14.30</td><td>ATCAAAGTGTT-GCCTCCAC
 :| |::|| |||||||
GGGGCCCGTAAGAGGAGGTG</td><td>2.42E-2</td></tr>
<tr><td>482. bta-miR-2347 MIMAT0011882</td><td>26555</td><td>-18.20</td><td>CTTTGGCT-GCCACCTCTGCT
 || ||: |||||||
CAAGTCGGAACAAGGAGACGT</td><td>3.46E-2</td></tr>
<tr><td>483. bta-miR-2284a MIMAT0011906</td><td>28050</td><td>-9.50</td><td>GTAGACCTTA--TAACTTTA
  |:|||::| ||||||
TTTTTGGGGTTGCTTGAAAA</td><td>2.5E-1</td></tr>
<tr><td>484. bta-miR-495 MIMAT0009335</td><td>28826</td><td>-18.60</td><td>TACAAGTCGCAGTATGTTTGTTT
   |||| ||| :||||||||||
TTCTTCA-CGTGGTACAAACAAA</td><td>1.04E-1</td></tr>
<tr><td>485. bta-miR-599 MIMAT0009354</td><td>29694</td><td>-12.10</td><td>TACTGGTA--CAGACACAAC
   ||:|| ||||||||
CAAACTATTTGACTGTGTTG</td><td>2.01E-1</td></tr>
<tr><td>486. bta-miR-6122-5p MIMAT0024594</td><td>29730</td><td>-9.10</td><td>GCCGATGGCAACCAGCGTCAACTGC
|| ||| || ||||||
CG-TGACC-TATTTC-ATGTTGACT</td><td>1.11E-1</td></tr>
<tr><td>487. bta-let-7g MIMAT0003838</td><td>30052</td><td>-17.10</td><td>CTGGCAACAGAACCCCTACCTCT
      |||:| | |||||||
TTGACATGTTT-GATGATGGAGT</td><td>2.94E-1</td></tr>
<tr><td>488. bta-let-7d MIMAT0003810</td><td>30054</td><td>-16.20</td><td>GGCAACAGAACC-CCTACCTCT
        |||| ||||||||
TTGATACGTTGGATGATGGAGA</td><td>2.94E-1</td></tr>
<tr><td>489. bta-miR-2414 MIMAT0011977</td><td>30065</td><td>-12.10</td><td>CCCTACC-TCTGGTGTAACACC
   :||| ||:| ||||||
ATTGTGGTAGGCGTGATTGTGT</td><td>2.94E-1</td></tr>
<tr><td>490. bta-miR-2411-3p MIMAT0011973</td><td>2496</td><td>-8.30</td><td>TTTAACCATGCCTAGTCAAGTTCAGA
             || || ||||||
CCTACACCCTCATTCTGT-CAAGTCG</td><td>2.65E-2</td></tr>
</table>

<table>
<tr><th>1. miR Name</th><th>leftmost position of predicted target site</th><th>folding energy (in - Kcal/mol)</th><th>heteroduplex</th><th>p value</th></tr>
<tr><td>491. bta-miR-10a MIMAT0003786</td><td>4286</td><td>-14.50</td><td>CTGATGCGAGGA-C-ACAGGGTA
||| | ||||||||
GTGTTTAAGCCTAGATGTCCCAT</td><td>1.75E-1</td></tr>
<tr><td>492. bta-miR-320a MIMAT0003534</td><td>5711</td><td>-11.40</td><td>GTGAACAGTCTTATCAGCTTTA
:| | || :|||||||
AGCGGGAGAGTTGGGTCGAAAA</td><td>1.62E-1</td></tr>
<tr><td>493. bta-miR-2284n MIMAT0011846</td><td>5797</td><td>-10.70</td><td>AAAAATTTGAAACAAACTTTTA
|||||:::||| ||||||||
TTTTTGGGCTT-ATTTGAAAAA</td><td>2.39E-2</td></tr>
<tr><td>494. bta-miR-2284c MIMAT0011926</td><td>5798</td><td>-8.90</td><td>AAAATTTGAAACAAACTTTTA
|||| : |||| |||||||
TTTT-GGTTTTGCTTGAAAAA</td><td>2.39E-2</td></tr>
<tr><td>495. bta-miR-320a MIMAT0003534</td><td>6738</td><td>-7.20</td><td>TAAGCTTGTAGCTTTAGCTTTT
:::|||||||
AGCGGGAGAGTTGGGTCGAAAA</td><td>4.7E-2</td></tr>
<tr><td>496. bta-miR-2284c MIMAT0011926</td><td>6928</td><td>-6.80</td><td>TGGATTAAGA-ACACTTTTA
::|::||:| ||||||
TTTTGGTTTTGCTTGAAAAA</td><td>2.28E-1</td></tr>
<tr><td>497. bta-miR-371 MIMAT0009301</td><td>7478</td><td>-20.80</td><td>ATGCT-ATGGCTAATGGTGGCACTG
:|| ::| ||||:||||||
TGTGAGTTTTG--TACCGCCGTGAA</td><td>2.95E-2</td></tr>
<tr><td>498. bta-miR-2284n MIMAT0011846</td><td>7546</td><td>-6.80</td><td>TATAAACCAGGTAATACTTTTA
|| :||| ||||||
TTTTTGGGCTTATT-TGAAAAA</td><td>2.94E-1</td></tr>
<tr><td>499. bta-miR-2284c MIMAT0011926</td><td>7548</td><td>-8.30</td><td>TAAACCAGGTAATACTTTTA
||||:: ||||||
TTTTGGTTTTGCTTGAAAAA</td><td>2.94E-1</td></tr>
<tr><td>500. bta-miR-2285aa MIMAT0030435</td><td>8447</td><td>-12.50</td><td>GTCTTAAAGGGGGT-GCAGTTTT
|:|: |||| || |||||||
CGGGTTTTCAAGCAAGGTCAAAA</td><td>3.29E-1</td></tr>
<tr><td>501. bta-miR-2284n MIMAT0011846</td><td>9693</td><td>-5.40</td><td>AATGGCTC-TTACTACTTTTA
::|:| ||||||
TTTTTGGGCTTATTTGAAAAA</td><td>1.98E-1</td></tr>
<tr><td>502. bta-miR-2447 MIMAT0012024</td><td>10050</td><td>-17.00</td><td>TAAGGTC-TACTG--TCCCAGA
:|:| ||:| |||||||
TCGTCGGTTTGGCCAAGGGTCT</td><td>1.75E-1</td></tr>
<tr><td>503. bta-miR-2411-3p MIMAT0011973</td><td>10515</td><td>-7.54</td><td>GGTTGT--CCAATTGCCCGTTCAGG
|| ||| | | ||||||
CCTACACCCTCATTCTGTCAAGTCG</td><td>8.51E-2</td></tr>
<tr><td>504. bta-miR-2355-3p MIMAT0011892</td><td>11367</td><td>-9.70</td><td>TGGTACTTACA-CATGGACAAC
| ||| || ||||||
TAGAGGTTTGTCGTTCCTGTTA</td><td>1.86E-1</td></tr>
</table>

1. miR Name	leftmost position of predicted target site	folding energy (in - Kcal/mol)	heteroduplex	p value
505. bta-miR-2355-3p MIMAT0011892	11952	-13.20	CGACGATTAC-GCAAAGGACAAT<br>|| |||| ||||||| TAGAGGTTTGTCGTT-CCTGTTA	2.39E-1
506. bta-miR-1251 MIMAT0009963	12086	-16.50	AGCAGTT-AAAACAGCTAGAGA<br>||| |:: ||||||||| TCG-CGGAAACCGTCGATCTCA	3.46E-2
507. bta-miR-2401 MIMAT0011958	12275	-15.50	GTAAGTTAGATAATCAAGCTCT<br>:||||:| |||||||| GTCGGGATCTGT--GTTCGAGA	2.14E-1
508. bta-miR-22-5p MIMAT0003826	14334	-13.50	ATTGGCT-ACCATTATAAAGAACT<br>:||| |||:| ||||||| ATTTCGAACGGTGA--CTTCTTGA	3.9E-1
509. bta-miR-320a MIMAT0003534	14425	-18.50	ATGCT-GCT-GATCCAGCTTTG<br>:||: || :|:|||||||| AGCGGGAGAGTTGGGTCGAAAA	7.54E-2
510. bta-miR-22-5p MIMAT0003826	16055	-6.10	TGAGTCCTTTTAC--AAGAACA<br>::|| |||||| ATTTCGAACGGTGACTTCTTGA	3.52E-1
511. bta-miR-708 MIMAT0009367	16793	-15.20	AGTAGCTAATTTAAGTGCTCCTA<br>:||||| || |||||| GGGTCGATCTAACATTCGAGGAA	1.34E-1
512. bta-miR-654 MIMAT0009359	16864	-6.70	ATAGTG-TG-CTTG-AGACATT<br>||| || | |||||| TTCCACTACCAGTCGTCTGTAT	2.82E-1
513. bta-miR-2355-3p MIMAT0011892	18194	-15.40	CACGCGTGATAGCATTGGGACAAA<br>:|:|||| :|||||| TAGAGGTTTGTCGT--TCCTGTTA	2.5E-1
514. bta-miR-708 MIMAT0009367	18308	-15.70	AAAGGCTGTGGCTAAAGCTCCTC<br>:|||: |||||||| GGGTCGATCTAACATTCGAGGAA	2.5E-1
515. bta-miR-2401 MIMAT0011958	18310	-15.10	AGGCTGTGG-CTAAAGCTCC<br>:||: |:| | |||||| GTCGGGATCTGTGTTCGAGA	2.5E-1
516. bta-miR-148a MIMAT0003522	20417	-10.90	TAGTAAGAGTGT-TTGCACTGT<br>||| ||||||| TGTTTCAAGACATCACGTGACT	2.21E-1
517. bta-miR-22-5p MIMAT0003826	20989	-7.20	ATGACCCTATTACT-AAGAACA<br>::||| |||||| ATTTCGAACGGTGACTTCTTGA	3.8E-1
518. bta-miR-12064 MIMAT0046771	23829	-12.30	GCT--TAATGG-TTACTACCC<br>||: || ||||||| CGGTGTGATCCTTATGATGGT	2.86E-1

<table>
<tr><th>1. miR Name</th><th>leftmost position of predicted target site</th><th>folding energy (in - Kcal/mol)</th><th>heteroduplex</th><th>p value</th></tr>
<tr><td>519. bta-miR-2402
MIMAT0011959</td><td>25173</td><td>-15.10</td><td>TATAGGCACTTGTCCTGCAGGTAC
    |||| || |||||||
ACGACCGT-CTCA--CCGTCCATA</td><td>1.89E-1</td></tr>
<tr><td>520. bta-miR-654
MIMAT0009359</td><td>25498</td><td>-9.20</td><td>CAAAAATCAAACA-CAGACATA
           || ||||||||
TTCCACTACCAGTCGTCTGTAT</td><td>2.95E-2</td></tr>
<tr><td>521. bta-miR-412
MIMAT0009313</td><td>27769</td><td>-9.00</td><td>ACATCCCTTCAATCATGTGAAGC
           |:|| ||||||
TGTCGATCACCTGGTCCACTTCA</td><td>2.11E-1</td></tr>
<tr><td>522. bta-miR-2299-5p
MIMAT0011807</td><td>29406</td><td>-9.20</td><td>ACTCCTGGTAAGCAATCCAGT
  || :| ||||||||
AAAGTCTCTTTACTTAGGTCA</td><td>3.16E-1</td></tr>
<tr><td>523. bta-miR-708
MIMAT0009367</td><td>29961</td><td>-16.80</td><td>GAAGGCTCAGGAAGGTCTGCTCCTA
   :||| :|| || ||||||
GGGTCGA--TCTAACATTCGAGGAA</td><td>2.01E-1</td></tr>
<tr><td>524. bta-miR-2285ag-3p
MIMAT0040923</td><td>30283</td><td>-15.90</td><td>GTGGTGGAGAAATGTTAAAACTTGGA
 |:|: :|:|| ||| |||||||
AATCGGTTTTT--CAA--GTGAACCT</td><td>4.98E-2</td></tr>
<tr><td>525. bta-miR-2375
MIMAT0011921</td><td>227</td><td>-19.50</td><td>AATACGGTCTCGAACTA-CACTGGG
     | ||| |:| |||||||
CTAGTCTGGAG--AGGTAGTGACCC</td><td>8.01E-2</td></tr>
<tr><td>526. bta-miR-2363
MIMAT0011901</td><td>3265</td><td>-11.90</td><td>GAGACATCTGATTCACAAGTTGA
      ||: ||||||||
TTTATAAGG-AACTGGTTCAACT</td><td>2.65E-1</td></tr>
<tr><td>527. bta-miR-449a
MIMAT0009320</td><td>3450</td><td>-16.90</td><td>GGCAGTGATGCAG-AAACTGCCC
: |||:   :||: ||||||
TGGTCG-ATTGTTATGTGACGGT</td><td>1.22E-2</td></tr>
<tr><td>528. bta-miR-23b-3p
MIMAT0003852</td><td>3654</td><td>-12.70</td><td>CTGGAAATGC-ATTAAATGTGAT
 ||| ||| | ||||||||
CACCATTAGGGACCGTTACACTA</td><td>3.34E-1</td></tr>
<tr><td>529. bta-miR-2285bk
MIMAT0046622</td><td>5795</td><td>-9.80</td><td>TTAAAAATTTGAAACAAACTTTT
  |||||:|: |||| |||||||
TCTTTTTGAG-TTTGCTTGAAAA</td><td>2.39E-2</td></tr>
<tr><td>530. bta-miR-374a
MIMAT0004342</td><td>5893</td><td>-8.50</td><td>TGTGACGGAGGTAAGTATTATAC
        |||| ||||||||
GTGAATAGTCCA-ACATAATATT</td><td>6.48E-2</td></tr>
<tr><td>531. bta-miR-12019
MIMAT0046708</td><td>6695</td><td>-13.30</td><td>ATAAAGTAATTTAC--ACCACAGA
    ||: |::|| |||||||
AGACTCG--AGGTGGTAGGTGTCT</td><td>1.06E-1</td></tr>
<tr><td>532. bta-miR-2351
MIMAT0011887</td><td>7884</td><td>-5.70</td><td>TGATAAAAATCTGATAACTAC
 :|:| ||| :|||||||
TTTGT-GTTATGTTATTGATA</td><td>3.48E-1</td></tr>
</table>

1. miR Name	leftmost position of predicted target site	folding energy (in -Kcal/mol)	heteroduplex	p value
533. bta-miR-2284z MIMAT0025559	9610	-7.80	GTTGTTTCAAATCATGCTTTTTG<br>::|||| || :|||||| TTTTTGGGTTT-GTTTGAAAAAA	6.88E-2
534. bta-let-7a-5p MIMAT0003844	11441	-11.40	TTTTGTATTTCACAGATATACCTCA<br>:|:||: || ||||||| TTGATATG--TTG-GATGATGGAGT	8.26E-2
535. bta-miR-154b MIMAT0025547	15709	-14.22	AAAATGACATAAACAATGGACCTCA |||| ||: :|||||| GCTTAC-GTG-GTACCTTCTGGAGA	1.15E-2
536. bta-miR-23b-3p MIMAT0003852	16246	-10.40	TGTGTAACGCACCAGG--ATGTGAT |||| || || ||||||| CACCATT--AGGGACCGTTACACTA	1.36E-1
537. bta-miR-2485 MIMAT0012079	18383	-7.50	GGATGTTGTTAGA-CCTAGAAT<br>:||: ||| |||||| TGATGGACG-TCTAAGATCTTT	2.5E-1
538. bta-miR-2313-3p MIMAT0011830	19029	-17.10	GGTTTGTGTATGTTTTGGAACTGT ||: :||:| |: |||||||| CCG--TACGT-CG-CACCTTGACC	2.42E-1
539. bta-miR-25 MIMAT0003853	19305	-10.40	ACTTGC-ATCACAAGATGCAATT<br>:| ||||| |||||| AGTCTGGCTCTGTTC-ACGTTAC	2.46E-1
540. bta-miR-2363 MIMAT0011901	19546	-7.80	CCATTATAAATGATAAAGTTGT |||: |||||| TTTATAAGGAACTGGTTCAACT	1.92E-1
541. bta-miR-2370-3p MIMAT0011915	21498	-14.50	TTAAGAATGG-CAGTTGCTTA |||| || ||||||||| ACCTCTTTCCTTTCAACGAAT	1.45E-1
542. bta-miR-346 MIMAT0009297	21501	-21.10	AGAATGGCAG--TTGCTTATGCAGACA ||| ||||| ||| ||||||| TCT--CCGTCCGTACG--CCCGTCTGT	1.45E-1
543. bta-miR-25 MIMAT0003853	21942	-5.70	GATTTCATAAACAGCATGCAATT |||: |||||| AGTCTGGCTCTGTT-CACGTTAC	9.19E-2
544. bta-miR-2400 MIMAT0011956	22011	-15.80	ATAGTT-AAGCT--TGGTGCTGA<br>:|||: |||| |||||| AGTCAGGCTCGACGGACACGACC	6.99E-2
545. bta-miR-124a MIMAT0003811	22664	-8.50	TTTACGCCTTATC--ATGCCTTT |:|:| |||||| GAACCGTAAGTGGCGCACGGAAT	1.24E-1
546. bta-miR-193a-3p MIMAT0003795	24103	-12.80	TTAGAGATCTCTGTTTGCCAGTA || || ||| |||||| TGACCCT-GAAACATCCGGTCAA	3.07E-1

<table>
<tr><th>1. miR Name</th><th>leftmost position of predicted target site</th><th>folding energy (in -Kcal/mol)</th><th>heteroduplex</th><th>p value</th></tr>
<tr><td>547. bta-miR-23b-3p
MIMAT0003852</td><td>24227</td><td>-11.40</td><td>AGCGTAATTTCACATATGATGTGAA
   ||||| | :||||||
CACCATTAGGG--ACCGTTACACTA</td><td>1.36E-1</td></tr>
<tr><td>548. bta-miR-2375
MIMAT0011921</td><td>24299</td><td>-10.40</td><td>ATGCATATTTT-ACAGACACTGGT
   || |::|: || ||||||
CTAGTCTGGAGAGGT-AGTGACCC</td><td>2.18E-1</td></tr>
<tr><td>549. bta-miR-346
MIMAT0009297</td><td>24728</td><td>-11.70</td><td>TGATGTCTTTTATTCAGGCAGACT
 || |:   :|| | |||||||
TCTCCG-TCCGTACGCCCGTCTGT</td><td>1.75E-1</td></tr>
<tr><td>550. bta-miR-16a
MIMAT0009242</td><td>24762</td><td>-14.40</td><td>TAATAATATT--GATGCTGCTA
   :|||||| ||||||||
GTGGTTATAAATGCACGACGAT</td><td>1.75E-1</td></tr>
<tr><td>551. bta-miR-2351
MIMAT0011887</td><td>24797</td><td>-5.70</td><td>GTTTTTCCAGCA-TAACTAT
         :|| |||||||
TTTGTGTTATGTTATTGATA</td><td>3.85E-1</td></tr>
<tr><td>552. bta-miR-2351
MIMAT0011887</td><td>24875</td><td>-7.90</td><td>ATTTGCAGTCTTTTAACTAT
    :||:| : |||||||
TTTGTGTTATGTTATTGATA</td><td>2.32E-1</td></tr>
<tr><td>553. bta-miR-374a
MIMAT0004342</td><td>24914</td><td>-10.70</td><td>CAAGTTGTCAGTTGTATTATAA
          ||||||||||||
GTGAATAGTCCAACATAATATT</td><td>9.48E-2</td></tr>
<tr><td>554. bta-miR-16a
MIMAT0009242</td><td>24928</td><td>-17.90</td><td>TATTATAATTTACCTGCTGCTA
:|::| |||||| ||||||||
GTGGTTATAAATGCACGACGAT</td><td>9.48E-2</td></tr>
<tr><td>555. bta-miR-25
MIMAT0003853</td><td>25752</td><td>-9.90</td><td>TCGGA--ATATTAAATGCAATT
||:|| :|| ||||||
AGTCTGGCTCTGTTCACGTTAC</td><td>2.5E-1</td></tr>
<tr><td>556. bta-miR-2375
MIMAT0011921</td><td>25938</td><td>-13.30</td><td>TCGTAGAGCGATT-ACCACTGGT
   :||| | |: ||||||
CTAGTCTGGAGAGGTAGTGACCC</td><td>2.05E-1</td></tr>
<tr><td>557. bta-miR-12047
MIMAT0046750</td><td>26567</td><td>-12.40</td><td>CCTCTGCTAGTCTGTTTCCTC
          :|||||||||
TCCGTTTCCAGGACAAAGGAC</td><td>3.46E-2</td></tr>
<tr><td>558. bta-miR-425-5p
MIMAT0003832</td><td>27000</td><td>-10.90</td><td>GGAGAAGGTTAATGAATGTGTCAA
      || ||| :||||||
AGTTGCCC--TCACTAGCACAGTA</td><td>1.75E-1</td></tr>
<tr><td>559. bta-miR-23b-3p
MIMAT0003852</td><td>27765</td><td>-12.10</td><td>TATTACATCCCTTCAATCATGTGAA
      |||||| ||||||
CACCATTAGGGA--CCGTTACACTA</td><td>2.11E-1</td></tr>
<tr><td>560. bta-miR-2285bk
MIMAT0046622</td><td>28048</td><td>-5.70</td><td>AGGTAGAC-CTTAT-AACTTTA
||: |:|| | |: ||||||
TCTTTTTGAGTTTGCTTGAAAA</td><td>2.5E-1</td></tr>
</table>

<table>
<tr><th>1. miR Name</th><th>leftmost position of predicted target site</th><th>folding energy (in - Kcal/mol)</th><th>heteroduplex</th><th>p value</th></tr>
<tr><td>561. bta-miR-23b-3p MIMAT0003852</td><td>29197</td><td>-8.40</td><td>CAGATTTGCC--AGCTTATGTGAC
        || || ||||||
CACCATTAGGGACCG-TTACACTA</td><td>2.11E-1</td></tr>
<tr><td>562. bta-miR-2285bk MIMAT0046622</td><td>29371</td><td>-5.06</td><td>TGAGAAATAATATCTAAACTTTA
 ||:|||: ||||||
TCTTTTTG-AGTTTGCTTGAAAA</td><td>3.85E-1</td></tr>
<tr><td>563. bta-miR-592 MIMAT0009353</td><td>29689</td><td>-7.70</td><td>AGGGGTACTGGTACAGACACAAC
          :|| |||||||
TGTAGTAGCGTATAACTGTGTTA</td><td>2.01E-1</td></tr>
<tr><td>564. bta-let-7a-5p MIMAT0003844</td><td>30054</td><td>-15.00</td><td>GGCAACAGAACC-CCTACCTCT
      | |||| |||||||
TTGATATGTTGGATGATGGAGT</td><td>2.94E-1</td></tr>
<tr><td>565. bta-miR-12019 MIMAT0046708</td><td>30130</td><td>-17.50</td><td>GCAAGGATGCCACTAAGCCACAGC
    :| ||||:| ||||||
AGACTC-GAGGTGGT-AGGTGTCT</td><td>2.72E-2</td></tr>
<tr><td>566. bta-miR-12019 MIMAT0046708</td><td>30302</td><td>-10.60</td><td>ACTTGGAACTAGTGACCCACAGT
 || :| |:| : ||||||
AGACTCGAGGTGG-TAGGTGTCT</td><td>4.98E-2</td></tr>
<tr><td>567. bta-miR-12019 MIMAT0046708</td><td>30560</td><td>-17.90</td><td>TATGAG-TCCAAAA-CCACAGC
  |||| |||| | ||||||
AGACTCGAGGTGGTAGGTGTCT</td><td>3.66E-2</td></tr>
<tr><td>568. bta-miR-449d MIMAT0011962</td><td>30676</td><td>-23.60</td><td>CTGCAGAGGACATCAGCCTTC
|| || || ||| ||||||||
GAGGTGTCGTGT-GTCGGAAG</td><td>1.28E-1</td></tr>
<tr><td>569. bta-miR-12004 MIMAT0046664</td><td>30942</td><td>-19.40</td><td>ACAAGTGCCCAAGGG-AAGAGCCAG
   || || ||:| ||||||||
TCCTC-CG-TCTCTCGGTCTCGGTC</td><td>6.29E-2</td></tr>
<tr><td>570. bta-miR-2397-3p MIMAT0011952</td><td>2197</td><td>-15.70</td><td>TTTACTACTAGC-ACTGCTCTT
       || || ||||||||
TACGCAAGAACGGTGACGAGAC</td><td>2.61E-1</td></tr>
<tr><td>571. bta-miR-1434-3p MIMAT0012040</td><td>3100</td><td>-8.60</td><td>CCTGAAGAT-GATGACTTTCTTG
||| |||: || ||||||
GGA-GTCTGGAATCT-AAAGAAG</td><td>1.72E-1</td></tr>
<tr><td>572. bta-miR-2284q MIMAT0011957</td><td>3433</td><td>-11.90</td><td>TGGTTGCGATCAGTTTTGGCA
     :||| |||||||
AGGCTTGCT-TGAAAAACCGG</td><td>1.22E-2</td></tr>
<tr><td>573. bta-miR-2429 MIMAT0011999</td><td>4814</td><td>-9.00</td><td>GCAGCCAGGATAAAGTTTTT
      ||| |||||||||
TTTTTTTCCCTTTTCAAAAA</td><td>3.16E-1</td></tr>
<tr><td>574. bta-miR-2285bt MIMAT0046639</td><td>5796</td><td>-5.70</td><td>TAAAAATTTGAAACAAACTTTTA
    ||:: || |||||||
TCTGTTGGGTTT-ACTTGAAAAA</td><td>2.39E-2</td></tr>
</table>

<table>
<tr><th>1. miR Name</th><th>leftmost position of predicted target site</th><th>folding energy (in -Kcal/mol)</th><th>heteroduplex</th><th>p value</th></tr>
<tr><td>575. bta-miR-200c
MIMAT0003823</td><td>7186</td><td>-5.90</td><td>CCATTGTTTGCTCTTATTAGTATTC
         :|:| :||||||
AGGTAGTAATGGG--CCGTCATAAT</td><td>2.98E-1</td></tr>
<tr><td>576. bta-miR-2285bt
MIMAT0046639</td><td>7545</td><td>-7.30</td><td>TTATAAACCAGGTAATACTTTTA
       |||::| ||||||
TCTGTTGGGTTTA-CTTGAAAAA</td><td>2.94E-1</td></tr>
<tr><td>577. bta-miR-6120-3p
MIMAT0024591</td><td>8177</td><td>-14.70</td><td>GTTGTAATAAC-TTGGTGCCAACATA
|:|:| || ||| ||||||||
CGATA--GGTGCAAC--AGGTTGTAT</td><td>3.62E-1</td></tr>
<tr><td>578. bta-miR-2429
MIMAT0011999</td><td>8449</td><td>-12.40</td><td>CTTAAAGGGGGTGCAGTTTTT
   |||:|||: |||||||
TTTTTTTCCCT-TTTCAAAAA</td><td>3.29E-1</td></tr>
<tr><td>579. bta-miR-1434-3p
MIMAT0012040</td><td>9271</td><td>-15.50</td><td>GCACAG-CCTGTGGATTTCTTG
   ||| ||| |:||||||||
GGAGTCTGGA-ATCTAAAGAAG</td><td>2.28E-1</td></tr>
<tr><td>580. bta-miR-3120
MIMAT0024572</td><td>9312</td><td>-25.10</td><td>TGCTGGTGCTATACTTGCTGTA
|||: || |||:|||||||||
ACGGACA-GATGTGAACGACAC</td><td>2.28E-1</td></tr>
<tr><td>581. bta-miR-2285bt
MIMAT0046639</td><td>9692</td><td>-8.10</td><td>AAATGGCTC-TTACTACTTTTA
  | :|:| ||||||
TCTGTTGGGTTTACTTGAAAAA</td><td>1.98E-1</td></tr>
<tr><td>582. bta-miR-1949
MIMAT0046755</td><td>9933</td><td>-15.20</td><td>AACTTCATTCTTGCAATCTGGTATT
|||| || ||| :|||||||
TTGA--TACGACCGTAGGACCATAT</td><td>7.65E-2</td></tr>
<tr><td>583. bta-miR-8548
MIMAT0037487</td><td>9958</td><td>-9.10</td><td>GTGAAAATGGTTAATCCTA
|:: || ||| ||||||
CGTGGTT-CCA-TTAGGAA</td><td>7.65E-2</td></tr>
<tr><td>584. bta-miR-12022
MIMAT0046715</td><td>10504</td><td>-10.80</td><td>AAGGATGCTCAGGT--TGTCCAA
||:| ::|| || |||||||
TTTCAGTGA--ACAGCACAGGTT</td><td>8.51E-2</td></tr>
<tr><td>585. bta-miR-195
MIMAT0004335</td><td>10560</td><td>-14.30</td><td>TGTAGCATGGCTTTATGCTGCTA
||: ||: |:| ||||||||
ACG-GTTATAAAGACACGACGAT</td><td>8.51E-2</td></tr>
<tr><td>586. bta-miR-2461-3p
MIMAT0012048</td><td>10665</td><td>-12.50</td><td>TAGC-CA-AGTT--AAGTCTGA
  || || |:| |||||||
TCCGTGTGACGAGAGTCAGACT</td><td>3.07E-1</td></tr>
<tr><td>587. bta-miR-200c
MIMAT0003823</td><td>11918</td><td>-12.00</td><td>ATAGCAAGTGCCTGACTAGTATTG
        |:||:| :||||||
AGGTAGTAATGGGC-CGTCATAAT</td><td>1.75E-1</td></tr>
<tr><td>588. bta-miR-2284o
MIMAT0012068</td><td>14337</td><td>-7.64</td><td>GGCTACCATTATAAAGAACTTG
|: ||| ||||||
CTTTTGG--GGCTTGCTTGAAA</td><td>3.9E-1</td></tr>
</table>

<table>
<tr><th>1. miR Name</th><th>leftmost position of predicted target site</th><th>folding energy (in -Kcal/mol)</th><th>heteroduplex</th><th>p value</th></tr>
<tr><td>589. bta-miR-3431
MIMAT0017394</td><td>16507</td><td>-20.30</td><td>AAACGCAAAAGGC-GACTGAGG
        ||||| ||||||||
TGTAGGTGTTCCGACTGACTCC</td><td>2.39E-1</td></tr>
<tr><td>590. bta-miR-12022
MIMAT0046715</td><td>18677</td><td>-13.80</td><td>TGATTTATATTGTAGTGTCCAT
    |:|: |||| |||||||
TTTCAGTG-AACAGCACAGGTT</td><td>2.36E-2</td></tr>
<tr><td>591. bta-miR-2397-3p
MIMAT0011952</td><td>18852</td><td>-20.00</td><td>ATGCTTAAAGCTGCCA-TGCTCTG
|||| ||||| |||||||
TACG--CAAGAACGGTGACGAGAC</td><td>6.11E-2</td></tr>
<tr><td>592. bta-miR-6120-3p
MIMAT0024591</td><td>19599</td><td>-6.39</td><td>TTTATT--AATAATACAACATA
 :|||: |||||||
CGATAGGTGCAACAGGTTGTAT</td><td>5.58E-3</td></tr>
<tr><td>593. bta-miR-219b-3p
MIMAT0040926</td><td>20012</td><td>-6.70</td><td>GGATGTCATCTTCAGC-CAATTCG
        ||: |:| ||||||
TGACTAACAGG-TTTGCGTTAAGA</td><td>7.09E-2</td></tr>
<tr><td>594. bta-miR-6120-3p
MIMAT0024591</td><td>20749</td><td>-8.54</td><td>AATATCTGAATACTACAACATT
  ||||:: ||||||
CGATAGGTGCAACAGGTTGTAT</td><td>1.92E-1</td></tr>
<tr><td>595. bta-miR-2429
MIMAT0011999</td><td>21445</td><td>-7.60</td><td>ATACAAATAAAGAAGTTTTT
          |:||||||||
TTTTTTTCCCTTTTCAAAAA</td><td>1.45E-1</td></tr>
<tr><td>596. bta-miR-219b-3p
MIMAT0040926</td><td>21943</td><td>-9.70</td><td>ATTTCATAAACAGCATGCAATTCA
          ||: |:|||||||
TGACTAACAGGTT-TGCGTTAAGA</td><td>9.19E-2</td></tr>
<tr><td>597. bta-miR-2461-3p
MIMAT0012048</td><td>23228</td><td>-19.50</td><td>TGGAACAATGCCAGGCAGTCTGA
 || ||| ||| ||||||||
TCCGTGTGACG-AGAGTCAGACT</td><td>1.89E-1</td></tr>
<tr><td>598. bta-miR-3120
MIMAT0024572</td><td>23666</td><td>-11.00</td><td>TACC-AATGGCTTTTGCTGTT
  || :| :|||||||
ACGGACAGATGTGAACGACAC</td><td>1.12E-1</td></tr>
<tr><td>599. bta-miR-2284x
MIMAT0017395</td><td>24682</td><td>-9.30</td><td>TGGGAACGTAAGACCTTTTCA
  :: || || |||||||
TTTTGGGCTTGCTTGAAAAGT</td><td>2.18E-1</td></tr>
<tr><td>600. bta-miR-195
MIMAT0004335</td><td>24760</td><td>-15.20</td><td>TGTAATAATATT--GATGCTGCTA
||: :|||||| ||||||||
ACG--GTTATAAAGACACGACGAT</td><td>1.75E-1</td></tr>
<tr><td>601. bta-miR-195
MIMAT0004335</td><td>24926</td><td>-13.30</td><td>TGTATTATAATTTACCTGCTGCTA
||: ||| ||| ||||||||
ACG-GTTAT-AAAGACACGACGAT</td><td>9.48E-2</td></tr>
<tr><td>602. bta-miR-2284o
MIMAT0012068</td><td>25654</td><td>-8.80</td><td>GACTACTTAACAAACAGAACTTT
|| ||:: ||| |||||||
CTTTTGGG--GCTTG-CTTGAAA</td><td>7.54E-2</td></tr>
</table>

<table>
<tr><th>1. miR Name</th><th>leftmost position of predicted target site</th><th>folding energy (in - Kcal/mol)</th><th>heteroduplex</th><th>p value</th></tr>
<tr><td>603. bta-miR-2397-3p MIMAT0011952</td><td>26510</td><td>-21.20</td><td>AAGTGTTGCCTCCACTGCTCTC
   |:||| |||||||||
TACGCAAGAACGGTGACGAGAC</td><td>2.42E-2</td></tr>
<tr><td>604. bta-miR-2461-3p MIMAT0012048</td><td>26558</td><td>-16.80</td><td>TGGCTGCCAC--CTCTGCTAGTCTGT
 ||| ||| |||| :||||||
TCCG--TGTGACGAGA--GTCAGACT</td><td>3.46E-2</td></tr>
<tr><td>605. bta-miR-2397-3p MIMAT0011952</td><td>26696</td><td>-12.50</td><td>ATGCATTT--AACAATGCTCTT
|||| ||: || ||||||
TACGCAAGAACGGTGACGAGAC</td><td>1.72E-1</td></tr>
<tr><td>606. bta-miR-12062 MIMAT0046769</td><td>27758</td><td>-13.00</td><td>GACGATATA-TTACATCCCT
   ||:|:| ||||||
TCACTGTGTCCTGGTAGGGT</td><td>2.11E-1</td></tr>
<tr><td>607. bta-miR-193a-5p MIMAT0003794</td><td>27781</td><td>-17.30</td><td>TCATGTGAAGCTAATTATAAGACCCA
|||| | ||: ||||||||
AGTAGA--GCGG--GCGTTTCTGGGT</td><td>2.11E-1</td></tr>
<tr><td>608. bta-miR-3120 MIMAT0024572</td><td>27829</td><td>-10.80</td><td>AGCTACCACAATGCCTGCTGTT
 ||: | |::| ||||||
ACGG-ACAGATGTGAACGACAC</td><td>2.25E-1</td></tr>
<tr><td>609. bta-miR-138 MIMAT0003813</td><td>29728</td><td>-16.30</td><td>CAGCC-GAT-GGCA--ACCAGCG
  ||| ||| :|| ||||||
GCCGGACTAAGTGTTGTGGTCGA</td><td>1.11E-1</td></tr>
<tr><td>610. bta-miR-219b-3p MIMAT0040926</td><td>30033</td><td>-9.10</td><td>TCGCGTAGT-AGAGC-CAATTCT
        || :|:| |||||||
TGACTAACAGGTTTGCGTTAAGA</td><td>2.94E-1</td></tr>
<tr><td>611. bta-miR-544b MIMAT0009345</td><td>3444</td><td>-14.80</td><td>AGTTTTGGCAGTGATGCAGAAA
   :||| || :||||||||
CTTGAACAATCTTTACGTCTTA</td><td>1.22E-2</td></tr>
<tr><td>612. bta-miR-2360 MIMAT0011898</td><td>3450</td><td>-16.10</td><td>GGCAGTGATGCAG-AAACTGCCCT
::| |: | ||| ||||||
TTG-CG-GAGGTCGGAGGACGGGC</td><td>1.22E-2</td></tr>
<tr><td>613. bta-miR-2357 MIMAT0011894</td><td>4167</td><td>-10.72</td><td>ACAGCA-GTTTGTTAAAGAGACCAC
   :|| |:|: ||||||
GAGTGTACGAG-ACACGGTCTGGTT</td><td>3.62E-1</td></tr>
<tr><td>614. bta-miR-2285db MIMAT0046709</td><td>5795</td><td>-12.20</td><td>TTAAAAATTTGAAACAAACTTTT
  ||||||:|||| |||||||
TCTTTTTAGACTT-GCTTGAAAA</td><td>2.39E-2</td></tr>
<tr><td>615. bta-miR-2285bx MIMAT0046644</td><td>5795</td><td>-7.70</td><td>TTAAAAATTTGAAACAAACTTTT
   |||| |:||| |||||||
TCCTTTTTAGCTT-GCTTGAAAA</td><td>2.39E-2</td></tr>
<tr><td>616. bta-miR-2284b MIMAT0011983</td><td>5797</td><td>-8.20</td><td>AAAAATTTGAAACAAACTTTT
 |||| : |||| |||||||
CTTTTTTGGTTTGCTTGAAAA</td><td>2.39E-2</td></tr>
</table>

1. miR Name	leftmost position of predicted target site	folding energy (in -Kcal/mol)	heteroduplex	p value
617. bta-miR-2285am-5p MIMAT0040950	5797	-9.50	AAAAATTTGAAACAAACTTTTA<br>|||||::|:|| ||||||| TTTTTGGATTT-GATTGAAAAA	2.39E-2
618. bta-miR-27b MIMAT0003546	5986	-14.60	ATAGGAC-ACACCGTCTGTGAT<br>:||:|| ||: |||||| CGTCTTGAATCGGT-GACACTT	2.94E-1
619. bta-miR-147 MIMAT0009237	6908	-11.10	TTGT--TGGTAAGATCGCACAG<br>|: :|:| :|||||| ATCGTCTTCGTAAAGGCGTGTG	2.28E-1
620. bta-miR-2285ch MIMAT0046671	7057	-6.20	GATAATTATAAAG-CCATTGAT<br>|||| ||||||| CGACCGGTTTTTCAAGTAACTA	2.42E-1
621. bta-miR-2333 MIMAT0011866	7516	-12.50	CATCAATGGA-ATTGCATTGA<br>| |||| :||||||| CCCGGGACCTCAGACGTAACG	2.95E-2
622. bta-miR-2333 MIMAT0011866	9274	-16.40	CAGCCTGTGGATTTCTTGGCATTGA<br>|||: |||| ||| |||||| CCCGGG-ACCT-CAGA--CGTAACG	2.28E-1
623. bta-miR-12008 MIMAT0046675	10400	-10.60	TAATAATGGGTGATTGTGTTAAAT<br>||:||| | |||||| GGGTTTTCCTACT-TCTCAATTTC	3.24E-2
624. bta-miR-182 MIMAT0009244	11671	-11.60	CTTTTGGGTATTGGAGGTGTGCCAAT<br>||:|| :|: : |||||| TCACACTCAAGATGGT--AACGGTTT	3.07E-1
625. bta-miR-1264 MIMAT0046656	12620	-16.90	ATAGTGGTCCAGATCAGACTTG<br>|:|| | ||:|| ||||||| TGTCCACGAGTTTATTCTGAAC	2.18E-1
626. bta-miR-2335 MIMAT0011868	14843	-5.90	GCTCTATTATGAAGCATTATCA<br>|| ||| ||||||| TAAGTCAATCAGTAGTAATAGA	6.08E-3
627. bta-miR-3660 MIMAT0025555	15671	-11.40	AAATAACGTTTTTATGTCAGA<br>|:|:|: |||||| AATTTTACGAGAGGACAGTCA	1.45E-1
628. bta-miR-544b MIMAT0009345	17731	-9.70	TATATTCACAGA-CTGCAGAAA<br>||| ||||||| CTTGAACAATCTTTACGTCTTA	1.09E-1
629. bta-miR-490 MIMAT0009331	19259	-22.70	TGGCATGGA-TGCTAAGCAGGTTG<br>:||||||| | || ||||||| GTCGTACCTCAGGA--GGTCCAAC	2.46E-1
630. bta-miR-2338 MIMAT0011872	20006	-20.30	GGGTCAGGATGTCATCTTCAGCCA<br>: ||||| : : ||:|||||| TGCAGTC-GGGGACAGGAGTCGGA	7.09E-2

1. miR Name	leftmost position of predicted target site	folding energy (in -Kcal/mol)	heteroduplex	p value
631. bta-miR-490 MIMAT0009331	20796	-10.90	TTGCATTTAG--GTGCAGGTTC<br>|||| || | |||||| GTCGTACCTCAGGAGGTCCAAC	2.61E-1
632. bta-miR-6534 MIMAT0025570	20832	-14.40	CCGGGTTCTG-CAGTTCTTAGG<br>:||| | |:|||||| TAGGCAGGACTGACGAGAATCT	2.78E-1
633. bta-miR-11980 MIMAT0046368	20844	-15.70	GTTC-TTAGGCAGTGGTTGCCT<br>|:| ::|:|| ||||||| GAGGCGGTTCG--GGCAACGGA	2.78E-1
634. bta-miR-544b MIMAT0009345	21111	-15.40	GAGTTTTCTTGGAATGCAGAAT<br>|| || |:|||||||||| CTTGAACAATCTTTACGTCTTA	3.16E-1
635. bta-miR-2338 MIMAT0011872	21653	-14.60	TCTTGCTATGCTGGATATTCAGCCT<br>: ||| :||||||| TGCAGTCGGGGAC--AGGAGTCGGA	2.25E-1
636. bta-miR-2324 MIMAT0011853	21970	-15.90	ATAAAATCACT--ATCAACCCCT<br>|||| :||||||| TGTCGGTGTGACGGGGTTGGGGT	9.19E-2
637. bta-miR-2357 MIMAT0011894	23607	-11.80	CTAGGCTGCATGATGCTTAGACCAT<br>|||: |||||| GAGTGTACGAGACACGG-TCTGGTT	3.29E-1
638. bta-miR-490 MIMAT0009331	23835	-11.97	TGGTTACTACCCTACTTCAGGTTC<br>:|: ||:|||||| GTCG--TACCTCAGGAGGTCCAAC	2.86E-1
639. bta-miR-299-2 MIMAT0046669	24052	-18.30	GTGGTAGTAC--AACCACATAC<br>:|||| ||| |||||||| TACCA-AATGGCAGGGTGTATG	1.2E-1
640. bta-miR-182 MIMAT0009244	25360	-9.50	GGTAATCCTTGTACT--TGCCAAC<br>|||: |||||| TCACACTCAAGATGGTAACGGTTT	1.16E-1
641. bta-miR-2285db MIMAT0046709	25655	-6.90	ACTACTTAACAAACAGAACTTTT<br>||| |||||||| TCTTTTTAGACTTG-CTTGAAAA	7.54E-2
642. bta-miR-2285bx MIMAT0046644	25655	-6.90	ACTACTTAACAAACAGAACTTTT<br>||| |||||||| TCCTTTTTAGCTTG-CTTGAAAA	7.54E-2
643. bta-miR-2284b MIMAT0011983	25656	-10.40	CTACTTAACAAACAGAACTTTT<br>||||| |||||||| CTTTTTTGGTTTG-CTTGAAAA	7.54E-2
644. bta-miR-12059 MIMAT0046766	26061	-16.30	TATAGGTA-ATATGGAGGAGT<br>|||:| ||||||| TCCTCCGTCCCACCCTCCTCT	3.76E-1

<table>
<tr><th>1. miR Name</th><th>leftmost position of predicted target site</th><th>folding energy (in -Kcal/mol)</th><th>heteroduplex</th><th>p value</th></tr>
<tr><td>645. bta-miR-27b MIMAT0003546</td><td>26167</td><td>-13.60</td><td>GAATATGGTAGCTTCTGTGAC
||||: ||||||
CGTCTTGAATCGGTGACACTT</td><td>4.38E-2</td></tr>
<tr><td>646. bta-miR-11980 MIMAT0046368</td><td>26503</td><td>-11.90</td><td>GGTATCAAAGT--GTTGCCT
|||: |||||||
GAGGCGGTTCGGGCAACGGA</td><td>2.42E-2</td></tr>
<tr><td>647. bta-miR-12048 MIMAT0046751</td><td>26569</td><td>-16.60</td><td>TCTG-CTAGTCTGTTTCCTCCTTT
|||| || : ||||||||
AGACCACGAAG-TGGAGGAGGAAC</td><td>3.46E-2</td></tr>
<tr><td>648. bta-miR-3660 MIMAT0025555</td><td>26577</td><td>-11.40</td><td>TCTGTTTCCTCCTTTGTCAGC
||| :||||||
AATTTTACGAGAGGACAGTCA</td><td>3.46E-2</td></tr>
<tr><td>649. bta-miR-2474 MIMAT0012065</td><td>27142</td><td>-15.50</td><td>ACTGC-GAAGGTTAGTCCCGGTC
|| | :|:| | ||||||
AGAGGAAGGTCGACC-GGGCCAT</td><td>2.14E-1</td></tr>
<tr><td>650. bta-miR-2333 MIMAT0011866</td><td>27153</td><td>-21.30</td><td>TAGTCCCGGTCTGTGCATTGC
|:|| || | ||||||||
CCCGGGACCTCAGACGTAACG</td><td>2.14E-1</td></tr>
<tr><td>651. bta-miR-2284b MIMAT0011983</td><td>28048</td><td>-7.60</td><td>AGGTAGACCTTAT-AACTTTA
|:||| |: ||||||
CTTTTTTGGTTTGCTTGAAAA</td><td>2.5E-1</td></tr>
<tr><td>652. bta-miR-182 MIMAT0009244</td><td>28062</td><td>-10.80</td><td>ACTTTAAGCATTATTAATTGCCAAA
|| :||::| ||||||||
TCACACTCAAGATGGT-AACGGTTT</td><td>2.5E-1</td></tr>
<tr><td>653. bta-miR-3660 MIMAT0025555</td><td>29181</td><td>-9.50</td><td>ACTGGCTATTCTT-TGTCAGA
:|||: ||||||
AATTTTACGAGAGGACAGTCA</td><td>2.11E-1</td></tr>
<tr><td>654. bta-miR-2285bx MIMAT0046644</td><td>29370</td><td>-5.44</td><td>TTGAGAAATAATATCTAAACTTTA
||:|||| ||||||
TCCTTTTTA--GCTTGCTTGAAAA</td><td>3.85E-1</td></tr>
<tr><td>655. bta-miR-2285db MIMAT0046709</td><td>29371</td><td>-5.86</td><td>TGAGAAATAATATCTAAACTTTA
||:|||| ||||||
TCTTTTTA-GACTTGCTTGAAAA</td><td>3.85E-1</td></tr>
<tr><td>656. bta-miR-182 MIMAT0009244</td><td>29778</td><td>-12.10</td><td>CTTG-GAACAGGACCGCATGCCAAA
|| || |||: |||||||
TCACACTCAAGATGGT-AACGGTTT</td><td>1.83E-1</td></tr>
<tr><td>657. bta-miR-2338 MIMAT0011872</td><td>30669</td><td>-15.30</td><td>GAGTTGACTGCAGAGGACATCAGCCT
||: |: | | | |||||||
TGCAGTCGGGG--ACAGG-AGTCGGA</td><td>1.28E-1</td></tr>
<tr><td>658. bta-miR-362-3p MIMAT0012548</td><td>3626</td><td>-9.80</td><td>CCTTATGTGATTGGCAGTGTGTT
|| |:| |||||||
CTTAGGAACTTATC--CACACAA</td><td>3.34E-1</td></tr>
</table>

<table>
<tr><th>1. miR Name</th><th>leftmost position of predicted target site</th><th>folding energy (in - Kcal/mol)</th><th>heteroduplex</th><th>p value</th></tr>
<tr><td>659. bta-miR-2284g MIMAT0011847</td><td>5797</td><td>-7.30</td><td>AAAAATTTGAAACAAACTTTT
||||| ::||| |||||||
TTTTTCGGCTT-GCTTGAAAA</td><td>2.39E-2</td></tr>
<tr><td>660. bta-miR-2891 MIMAT0013849</td><td>6297</td><td>-14.20</td><td>TGCTAA-AGAACCCAAAGAA
 :|||| ||||||||
TTGATTGGATCGGGTTTCTA</td><td>7.07E-3</td></tr>
<tr><td>661. bta-miR-199a-3p MIMAT0003746</td><td>7884</td><td>-10.00</td><td>TGATAAAAATCTGATAACTACTGC
|:|: ||| || |||||||
ATTG--GTTACACGTCTGATGACA</td><td>3.48E-1</td></tr>
<tr><td>662. bta-miR-2395 MIMAT0011948</td><td>8180</td><td>-12.79</td><td>GTAATAACTTGGTGCCAACAT
|| | ||||||||
CAGACTCTCTCGACGGTTGTC</td><td>3.62E-1</td></tr>
<tr><td>663. bta-miR-2315 MIMAT0011835</td><td>8240</td><td>-13.90</td><td>TTCTGATTCAAAA-TTCTGCT
     ||:||||| |||||||
GGTCATAGGTTTTTAAGACGA</td><td>3.07E-1</td></tr>
<tr><td>664. bta-miR-2315 MIMAT0011835</td><td>8820</td><td>-8.40</td><td>TACACATGCA--CTTTCTGCT
     || || |||||||
GGTCATAGGTTTTTAAGACGA</td><td>1.54E-1</td></tr>
<tr><td>665. bta-miR-362-3p MIMAT0012548</td><td>9410</td><td>-11.20</td><td>ATGTGATTG-TGTGGTGTGTA
  :| ||| |||||||
CTTAGGAACTTATCCACACAA</td><td>1.92E-1</td></tr>
<tr><td>666. bta-miR-2315 MIMAT0011835</td><td>10064</td><td>-14.50</td><td>CCAGACATGTGATATGTTCTGCT
|||| || ::| :|||||||
GGTC-ATAGGTT-TTTAAGACGA</td><td>1.75E-1</td></tr>
<tr><td>667. bta-miR-2315 MIMAT0011835</td><td>12058</td><td>-14.70</td><td>GCGTGTTCTAGTGGTTCTGCT
      ||:|:   :|||||||
GGTCATAGGTTTTTAAGACGA</td><td>3.46E-2</td></tr>
<tr><td>668. bta-miR-11998 MIMAT0046654</td><td>12087</td><td>-14.50</td><td>GCAGTTAAAACA-GCTAGAGAA
      |||||| :||||||||
CAACCCTTTTGTGTGATCTCTT</td><td>3.46E-2</td></tr>
<tr><td>669. bta-miR-129 MIMAT0009220</td><td>12714</td><td>-5.50</td><td>TGATGGTCTTAAGTATACAAAAAT
    :|:|: || ||||||
TCGTTCGGG--TCTGGCGTTTTTC</td><td>3.34E-1</td></tr>
<tr><td>670. bta-miR-148d MIMAT0046670</td><td>14335</td><td>-9.00</td><td>TTGGCTACCATTATAAAGAACTTG
        ||:||: |||||||
TCAGTCACGTGATG--TCTTGAAA</td><td>3.9E-1</td></tr>
<tr><td>671. bta-miR-2484 MIMAT0012077</td><td>16411</td><td>-9.30</td><td>ATGATTTTAA--TCGT-ATAGCTA
||| |:|| ||:| ||||||
TACGTTAGTTTCAGTAGTATCGAG</td><td>3.57E-1</td></tr>
<tr><td>672. bta-miR-32 MIMAT0009283</td><td>19300</td><td>-9.30</td><td>CCGCCACTTGCATCACAAGATGCAATT
  || ||||: ||| | ||||||
TACGTTGAAT-CAGT--AC-ACGTTAT</td><td>2.46E-1</td></tr>
</table>

1. miR Name	leftmost position of predicted target site	folding energy (in -Kcal/mol)	heteroduplex	p value
673. bta-miR-2285cg MIMAT0046665	20279	-9.20	CCAGAAG-ATTATTGGTGGTTTG<br>||:||| |:||| |||||| AGTTTTCAAAGTAA-GACCAAAA	1.95E-1
674. bta-miR-2435 MIMAT0012007	20775	-10.30	GTACCTGTTAATATGCGAGTTT<br>:|| ||||||| CGGTTTCTCAAGTAAGCTCAAA	2.61E-1
675. bta-miR-2315 MIMAT0011835	21158	-8.00	ATTTTGGACGG-TTTTCTGCA<br>|: |:: |||||| GGTCATAGGTTTTTAAGACGA	2.01E-1
676. bta-miR-11998 MIMAT0046654	21422	-13.60	AAAGGGTAAACTACTTGTTAGAGAT<br>||| |||| || :|||||| CAACCCTTTTG-TG--TGATCTCTT	1.45E-1
677. bta-miR-2285dd MIMAT0046712	22028	-7.44	TGAAAAG--AAAGATGGTTTTT<br>:||||| |||||| ATTTTTCAAGTAAGTCCAAAAG	6.99E-2
678. bta-miR-759 MIMAT0009371	23227	-13.90	GTGGAACAATGCCAGGCAGTCTGA<br>|| :|| || |||||||| CAGTTT-TAAC-AAACGTCAGACG	1.89E-1
679. bta-miR-142-3p MIMAT0003791	24515	-9.50	GTGAGATTAAGTGTAAAACACT<br>||||: ||||||| GTAGGTATTTCATCCTTTGTGA	5.76E-2
680. bta-miR-199a-3p MIMAT0003746	24532	-9.50	ACACTATCTATAGCACCATCTACTGG<br>||:| ||| |||||| ATTGGT--TACACGT--CTGATGACA	5.76E-2
681. bta-miR-424-5p MIMAT0013593	24762	-11.40	TAATAATATTGA--TGCTGCTA<br>||:|| || ||||||| AGTTTTGTA-CTTAACGACGAC	1.75E-1
682. bta-miR-199a-3p MIMAT0003746	25475	-9.40	GTACTA-CTTGT--TCTACTGA<br>||:| ||: |||||| ATTGGTTACACGTCTGATGACA	2.95E-2
683. bta-miR-2285cg MIMAT0046665	25627	-7.20	TCTAATGGTAATCTCTATGGTTTT<br>|| :|| || ||||||| AG-TTTTCA-AAGTAAGACCAAAA	2.65E-1
684. bta-miR-148d MIMAT0046670	25655	-10.70	ACTACTTAACAAACAGAACTTT<br>|||||||||| TCAGTCACGTGATGTCTTGAAA	7.54E-2
685. bta-miR-2284g MIMAT0011847	25657	-6.90	TACTTAACAAACAGAACTTTT<br>||| |||||||| TTTTTCGGCTTG-CTTGAAAA	7.54E-2
686. bta-miR-2285cg MIMAT0046665	27372	-9.82	GGAAGAGTTGGATCAATGGTTTA<br>||:|||| |||||| AGTTTTCAA-AGTAAGACCAAAA	1.61E-2

1. miR Name	leftmost position of predicted target site	folding energy (in - Kcal/mol)	heteroduplex	p value
687. bta-miR-876<br>MIMAT0009380	28279	-7.60	TCACTCTTTATGG-CAAATCCA<br>||:| : ||||||| ACCACTAAGTGTTTCTTTAGGT	6.2E-2
688. bta-miR-32<br>MIMAT0009283	28796	-6.40	TTTTATTACAATCATATTGCAATT<br>|||| |||||| TACGTTGAATCAGTA-CACGTTAT	1.04E-1
689. bta-miR-129<br>MIMAT0009220	29599	-10.10	GAATTACTCAG-TTTCAAAAAG<br>|:||| : ||||||| TCGTTCGGGTCTGGCGTTTTTC	8.64E-2
690. bta-miR-199a-3p<br>MIMAT0003746	29661	-14.30	GCACCAGGAGTCCCAG-CTACTGA<br>||||: || ||| |||||| ATTGGTT-ACA-CGTCTGATGACA	2.69E-1
691. bta-miR-507b<br>MIMAT0046744	30252	-12.40	TTTGGGAAGAGAGGCCCCAATCA<br>||:| ||||||| AGGTGAGATTCTTCATGGTTAGT	4.98E-2
692. bta-miR-2441<br>MIMAT0012016	354	-14.90	AACAGGCGGAATTTGTCCTGA<br>:| ::||||||| GAGGGTG-ACAGGACAGGACA	1.54E-1
693. bta-miR-105a<br>MIMAT0009217	1722	-5.59	TGTAAAGGAAACAAATCTCATTTGC<br>|||| |||||| TGGTGTCCT--CAGACTCGTAAACT	3.11E-2
694. bta-miR-2285cz<br>MIMAT0046706	2213	-7.40	CTCTTGCATTTGCATGGGTTTT<br>|||:: :|||||| TGTTTTTTAAGTAAGCCCAAAC	2.61E-1
695. bta-miR-2285j<br>MIMAT0024585	2214	-6.90	TCTTGCATTTGCATGGGTTTTA<br>||:| |||||| GTTTTTCAAGCAAGACCAAAAA	2.61E-1
696. bta-miR-2284j<br>MIMAT0011827	5798	-10.80	AAAATTTGAAACAAACTTTTA<br>||||::|||| ||||||| TTTTGGACTT-GCTTGAAAAG	2.39E-2
697. bta-miR-105a<br>MIMAT0009217	5932	-6.97	GCC-CA--ATTTAAAACATTTGA<br>:|| || ||||||| TGGTGTCCTCAGACTCGTAAACT	6.48E-2
698. bta-miR-11991<br>MIMAT0046631	6923	-10.00	CACAGT--GGATTAAGAACAC<br>|||:| :||| |||||| ATGTTAGTTCTATGTCTTGTA	2.28E-1
699. bta-miR-2284j<br>MIMAT0011827	7548	-5.50	TAAACCAGGTAATACTTTTA<br>||| |: |||||| TTTTGGACTTGCTTGAAAAG	2.94E-1
700. bta-miR-2285bm<br>MIMAT0046624	8451	-6.80	TAAAGGGGGTGC--AGTTTTTA<br>:|||::| ::| |||||| GTTTTTCAGGTGAGCCAAAAAA	3.29E-1

<table>
<tr><th>1. miR Name</th><th>leftmost position of predicted target site</th><th>folding energy (in -Kcal/mol)</th><th>heteroduplex</th><th>p value</th></tr>
<tr><td>701. bta-miR-2419-3p MIMAT0011986</td><td>9897</td><td>-15.20</td><td>TGATGTGCTTTACCAACCGCCTA
|||| :| ||| || ||||||
ACTA-GCATAATCGTGGGCGGAC</td><td>1.42E-1</td></tr>
<tr><td>702. bta-miR-584 MIMAT0009352</td><td>10298</td><td>-14.80</td><td>CTTA-TAAC-GGCAAACCA
||:| | | |||||||||
GAGTCAGGGTCCGTTTGGT</td><td>3.62E-1</td></tr>
<tr><td>703. bta-miR-2296 MIMAT0011804</td><td>10502</td><td>-21.10</td><td>ATAAGGATGCTCAGGTTGTCCA
|||| ||||| |||||||
TCGTCCTTGGAGTC-AACAGGT</td><td>8.51E-2</td></tr>
<tr><td>704. bta-miR-376a MIMAT0009946</td><td>10692</td><td>-10.50</td><td>AGATGCTTTAGCTTCTATGAC
|| || |:|||||||
TGCACCTAAAAGGAGATACTA</td><td>3.07E-1</td></tr>
<tr><td>705. bta-miR-2353 MIMAT0011889</td><td>11967</td><td>-19.20</td><td>GGACAATACTGTTTTGCAGGCTT
|||| :||||||||
GGTCAGGAGACATGACGTCCGAT</td><td>2.39E-1</td></tr>
<tr><td>706. bta-miR-2452 MIMAT0012033</td><td>12069</td><td>-8.30</td><td>TGGTTCTGCTAATCAACAGCAG
||: |||||||
TTTTTTCCTTTTGTTTGTCGTG</td><td>3.46E-2</td></tr>
<tr><td>707. bta-miR-2471-5p MIMAT0012061</td><td>13203</td><td>-8.90</td><td>GCCTGTAGGTATAAAAGATCCTG
|:| |||||||
GGAGACTCTAATCTGTCTAGGAG</td><td>3.29E-1</td></tr>
<tr><td>708. bta-miR-105a MIMAT0009217</td><td>13609</td><td>-8.30</td><td>AACACG-ATTTCTTTACATTTGA
|||: ||| |||||||
TGGTGTCCTCAGACTCGTAAACT</td><td>2.73E-1</td></tr>
<tr><td>709. bta-miR-181b MIMAT0003793</td><td>14994</td><td>-12.40</td><td>AGTACTATGACTGGCA--GAATGTT
:||| :||| |||||||
TTGGGTGGCTG-TCGTTACTTACAA</td><td>9.48E-2</td></tr>
<tr><td>710. bta-miR-433 MIMAT0009318</td><td>15721</td><td>-11.50</td><td>ACA--ATGGA-CC--TCATGAA
||| ||| || ||||||
TGTGGCTCCTCGGGTAGTACTA</td><td>1.15E-2</td></tr>
<tr><td>711. bta-miR-30e-5p MIMAT0003799</td><td>17144</td><td>-9.60</td><td>TGACACCACTCGTAAGTATGTGTTTACT
||| :||| :|||||||
TCGAAGGT--CAGTTC--CTACAAATGT</td><td>3.39E-1</td></tr>
<tr><td>712. bta-miR-2285cz MIMAT0046706</td><td>17660</td><td>-11.80</td><td>AGCTAGGCGCGTTTTGGGTTTG
||:|||||||
TGTTTTTTAAGTAAGCCCAAAC</td><td>3.29E-1</td></tr>
<tr><td>713. bta-miR-433 MIMAT0009318</td><td>18660</td><td>-9.90</td><td>TCTTTATCAAGT-AATCATGAT
||: ||||||||
TGTGGCTCCTCGGGTAGTACTA</td><td>2.36E-2</td></tr>
<tr><td>714. bta-miR-376a MIMAT0009946</td><td>18934</td><td>-11.70</td><td>ATTTTGATTTTAAGTTCTATGAT
|||||| :||||||||
TGCACCTAAAA--GGAGATACTA</td><td>2.25E-1</td></tr>
</table>

1. miR Name	leftmost position of predicted target site	folding energy (in -Kcal/mol)	heteroduplex	p value
715. bta-miR-12035 MIMAT0046728	19514	-15.90	TTATACAGGACAGG--CTGGTGA<br>:||||:| |||||| AGACCCCTCTGTTCAGGACCACA	1.98E-1
716. bta-miR-2325a MIMAT0011854	19876	-6.90	TTTACATTTCCACGACAAAAG<br>:||| :|||||| CTTTTTTTGGGT-TTGTTTTG	3.48E-1
717. bta-miR-105a MIMAT0009217	20286	-13.00	ATTATTGGTGGTTT--GCATTTGT<br>|::|: || :||:| ||||||| TGGTGTCC-TCAGACTCGTAAACT	1.95E-1
718. bta-miR-11991 MIMAT0046631	20988	-5.90	TATGACCCTATTACTAAGAACAT<br>||| ||||||| ATGTTAGTTCTATG--TCTTGTA	3.8E-1
719. bta-miR-2285j MIMAT0024585	21153	-5.80	TTTGCATTTTGGAC-GGTTTTC<br>||:| | |||||| GTTTTTCAAGCAAGACCAAAAA	2.01E-1
720. bta-miR-2285j MIMAT0024585	22028	-9.50	TGAAAAGAAAG--ATGGTTTTT<br>:||||| | ||||||| GTTTTTCAAGCAAGACCAAAAA	6.99E-2
721. bta-miR-376a MIMAT0009946	22099	-7.10	CCACTGATAAGCTAGCTATGAT<br>||| |: ||||||| TGCACCTAAAAGG-AGATACTA	3.48E-1
722. bta-miR-15b MIMAT0003792	24760	-15.90	TGTAA-TAATATTGATGCTGCTA<br>||||| || || |||||||| ACATTTGGTACTAC-ACGACGAT	1.75E-1
723. bta-miR-2285j MIMAT0024585	25628	-6.20	CTAATGGTAATCTCTATGGTTTTA<br>:|| || ||||||| GTTTTTCA--AGCAAGACCAAAAA	2.65E-1
724. bta-miR-2284j MIMAT0011827	25660	-7.70	TTAAC--AAACAGAACTTTTA<br>||| ||| |||||||| TTTTGGACTTG-CTTGAAAAG	7.54E-2
725. bta-miR-181b MIMAT0003793	26995	-10.70	GCTATGGAGAAGGTTAATGAATGTG<br>|| :|: ||||||||| TTGGGTGGCTGTCG-TTACTTACAA	1.75E-1
726. bta-miR-2386 MIMAT0011938	27386	-12.20	AATGGTTTAAAAACCAAACA<br>||||||||||| CCTTTTGCTTTTTGGTTTGT	1.61E-2
727. bta-miR-2386 MIMAT0011938	28283	-9.20	TCTTTATGGCAAATCCAAACA<br>|:|: ||| ||||||| CCTTTTGCTTTTT-GGTTTGT	6.2E-2
728. bta-miR-1247-3p MIMAT0025550	30307	-17.80	GAACTAGTGACCC-ACAGTTCCCC<br>|:||| ||| || |||||| CGAGGTCA--GGGCTG-CAAGGGC	4.98E-2

<table>
<tr><th>1. miR Name</th><th>leftmost position of predicted target site</th><th>folding energy (in - Kcal/mol)</th><th>heteroduplex</th><th>p value</th></tr>
<tr><td>729. bta-miR-763
MIMAT0009374</td><td>30336</td><td>-18.70</td><td>GCAGAACT--CGCACCCACAGCTGG
|| ||| | || |||||||
CG--GTGACCAAGGAGG-GTCGACC</td><td>4.98E-2</td></tr>
<tr><td>730. bta-miR-2367-3p
MIMAT0011910</td><td>30468</td><td>-6.50</td><td>AGATTTGACAGTACACTTTCAG
::|| ||| | ||||||
GAAGGACATTCACG-GAAAGTT</td><td>3.29E-1</td></tr>
<tr><td>731. bta-miR-2461-5p
MIMAT0012047</td><td>1128</td><td>-16.00</td><td>TGTTATGAGAC--TGCAGAGTGC
||::| || | :|||||||
ACGGTCCTAGGCTCTGTCTCACC</td><td>1.69E-1</td></tr>
<tr><td>732. bta-miR-374c
MIMAT0046394</td><td>1708</td><td>-7.10</td><td>GGTTGCAAGG--CTATTGTAA
||: ||| ||||||||
TGAATAGTCCAACATAACATT</td><td>3.11E-2</td></tr>
<tr><td>733. bta-miR-363
MIMAT0003855</td><td>5646</td><td>-11.30</td><td>TAAGGGTGTAGGAAGTGCAAA
:|:|| : ||||||
GCGTCTACCTATGGCACGTTA</td><td>9.48E-2</td></tr>
<tr><td>734. bta-miR-2376
MIMAT0011922</td><td>6268</td><td>-17.70</td><td>GACGATGGTGGTGATATCTCAGAGA
|| |:| | |||||||||
TCGC--GGATCGTTCTAGAGTCTCC</td><td>3.52E-1</td></tr>
<tr><td>735. bta-miR-11974
MIMAT0046353</td><td>6785</td><td>-19.60</td><td>GGAGTGTGGTTGC-TAG-AGGTGCT
||| || :| :|| |||||||
CGACAC-CCCCTGTGTCGTCCACGA</td><td>3.52E-1</td></tr>
<tr><td>736. bta-miR-210
MIMAT0003824</td><td>6904</td><td>-16.60</td><td>ACTTTTGTTGGTAAGATCGCACAGT
::|:|| ||||||||
AGTCGGCGAC--AGTGTGCGTGTCA</td><td>2.28E-1</td></tr>
<tr><td>737. bta-miR-2358
MIMAT0011896</td><td>8185</td><td>-13.80</td><td>AACTTGGTGCCAACATATTTGA
:|:| ||||| ||||||
AGGTGCTAGGGTTG-ATAAACC</td><td>3.62E-1</td></tr>
<tr><td>738. bta-miR-19b
MIMAT0004337</td><td>8794</td><td>-10.00</td><td>TATGGTTACCATG-TGTTGCACT
|||| ||||||
AGTCAAAACGTACCTAAACGTGT</td><td>1.54E-1</td></tr>
<tr><td>739. bta-miR-2285z
MIMAT0029950</td><td>8816</td><td>-10.20</td><td>TTATTACACATGCACTTTCTGC
||| ||||||||
TCCTGGACTTACTTGAAAGACC</td><td>1.54E-1</td></tr>
<tr><td>740. bta-miR-12017
MIMAT0046697</td><td>8893</td><td>-11.92</td><td>GCTAGTGGCTGTGTGCTTTCCTCTGC
:|| || |||||||
CAAGTAC-AGTTTTCG--AGGAGACA</td><td>2.69E-1</td></tr>
<tr><td>741. bta-miR-221
MIMAT0003529</td><td>10546</td><td>-11.60</td><td>CAATCT-GTTAATTTTGTAGCA
||:|: |: |: ||||||
TTTGGGTCGTCTGTTACATCGA</td><td>8.51E-2</td></tr>
<tr><td>742. bta-miR-19b
MIMAT0004337</td><td>11805</td><td>-12.50</td><td>GCAATATTGTAGCACTTTGCACA
|||:| ||||||||
AGTCAAAACGTACCTAAACGTGT</td><td>6.2E-2</td></tr>
</table>

<table>
<tr><th>1. miR Name</th><th>leftmost position of predicted target site</th><th>folding energy (in - Kcal/mol)</th><th>heteroduplex</th><th>p value</th></tr>
<tr><td>743. bta-miR-92b MIMAT0009384</td><td>12327</td><td>-7.50</td><td>TTGTGTACCATTGAATGCAATC
|:   :|| ||||||
CCTCCGGCCCTGCTCACGTTAT</td><td>9.78E-2</td></tr>
<tr><td>744. bta-miR-2385-5p MIMAT0011935</td><td>13977</td><td>-16.90</td><td>GAC-TATGTTATTGCAGCCCCAG
:|| ||: ::|:| |||||||
TTGCATGAGGTGA--ACGGGGTC</td><td>3.48E-1</td></tr>
<tr><td>745. bta-miR-10164-3p MIMAT0040911</td><td>14512</td><td>-11.30</td><td>GCGGTGTAAAATTTCAAACAGTTA
|:|: :|| :|:| |||||||
CGTCG-GTTCGGAGGATTGTCAAC</td><td>5.76E-2</td></tr>
<tr><td>746. bta-miR-2319b MIMAT0011843</td><td>16083</td><td>-19.50</td><td>AGAAGTGCAGTTATGCAGAGTG
|||||| ||:||||||
GATTCACG-GATTATGTCTCAT</td><td>3.52E-1</td></tr>
<tr><td>747. bta-miR-12054 MIMAT0046761</td><td>20113</td><td>-15.30</td><td>ATGC-TCTGGCAACCTCCAC
||| ||: |||||||
CACGTAGG--GGTGGAGGTC</td><td>2.82E-1</td></tr>
<tr><td>748. bta-miR-2461-5p MIMAT0012047</td><td>20393</td><td>-15.40</td><td>TATCA-CTGATGAGA-AGAGTGG
:|| :|||| |||||||
ACGGTCCTAGGCTCTGTCTCACC</td><td>2.21E-1</td></tr>
<tr><td>749. bta-miR-19b MIMAT0004337</td><td>20410</td><td>-10.10</td><td>GTGGTGGTAGTAAGAGTGTTTGCACT
:|| | |:| | | :|||||||
AGTCAAAA-CGTAC-C-TAAACGTGT</td><td>2.21E-1</td></tr>
<tr><td>750. bta-miR-2313-5p MIMAT0011829</td><td>21155</td><td>-13.60</td><td>TGCATTTTGGACGGTTTTCTGCACA
||| |||| |: ||||||
ACG--GAACC-AGGAGTCGACGTGA</td><td>2.01E-1</td></tr>
<tr><td>751. bta-miR-2358 MIMAT0011896</td><td>21214</td><td>-9.60</td><td>TTGGC-AT--AAATTATTTGG
|: :| || ||:|||||||
AGGTGCTAGGGTTGATAAACC</td><td>2.01E-1</td></tr>
<tr><td>752. bta-miR-10164-3p MIMAT0040911</td><td>21281</td><td>-11.40</td><td>GTTTTGGAGAAATTCC--ACAGTTT
|: : :|| :||| ||||||
CGTCGGTTC--GGAGGATTGTCAAC</td><td>7.65E-2</td></tr>
<tr><td>753. bta-miR-19b MIMAT0004337</td><td>21871</td><td>-13.10</td><td>CCAGGAAATGGATTGGACATTGCACC
||| || || ||| ||||||
AGTC-AAAACGTA-CCT-AAACGTGT</td><td>2.65E-1</td></tr>
<tr><td>754. bta-miR-2358 MIMAT0011896</td><td>24860</td><td>-11.50</td><td>TGGGCAATTTGGGCTATTTGC
:| ||:: ::|||||||
AGGTGCTAGGGTTGATAAACC</td><td>2.32E-1</td></tr>
<tr><td>755. bta-miR-10164-3p MIMAT0040911</td><td>26221</td><td>-8.30</td><td>CTACTTGA-C--ACTACACAGTTG
: | | ||| |||||||
CGTCGGTTCGGAGGAT-TGTCAAC</td><td>1.95E-1</td></tr>
<tr><td>756. bta-miR-12017 MIMAT0046697</td><td>26550</td><td>-11.10</td><td>ATACACTTTGGCTGCCACCTCTGC
|| | : || ||||||
CAAGTACAGTTTTCG-AGGAGACA</td><td>3.46E-2</td></tr>
</table>

1. miR Name	leftmost position of predicted target site	folding energy (in -Kcal/mol)	heteroduplex	p value
757. bta-miR-12054 MIMAT0046761	27347	-16.80	CAACA-CCCAACCTCCAT<br>|| ||| ||||||| CACGTAGGGGTGGAGGTC	6.88E-2
758. bta-miR-12017 MIMAT0046697	30051	-10.10	TCTGGCAACAGAACCCCTACCTCTGG<br>||:|| || |||||| CAAGTACAGTTTT--CGA-GGAGACA	2.94E-1
759. bta-miR-10164-3p MIMAT0040911	30304	-8.00	TTGGAACTAGTGACC-CACAGTTC<br>:| ||: || |||||| CGTC-GGTTCGGAGGATTGTCAAC	4.98E-2
760. bta-miR-12037 MIMAT0046732	240	-7.00	ACTACACTGGG-CTCCAGAATT<br>|||| |:| |||||| TGAT--TTCTCTTCTGTCTTAC	8.01E-2
761. bta-miR-205 MIMAT0003545	1030	-12.30	AAGACATTGCAAGAAATGAAGGC<br>|||| ::| | |||||||| GTCTGAGGC-CACCTTACTTCCT	2.35E-1
762. bta-miR-2285bp MIMAT0046633	1673	-8.00	AGTCTTATGACGGTTTGGTTTA<br>|| |:|||||| TGTGTTTCAAGCAAGACCAAAA	3.11E-2
763. bta-miR-545-5p MIMAT0003806	1912	-10.10	AGTTTAG-AAACTTTTACTGT<br>:|::|: |||| ||||||| GTAGGTTATTTGTAAATGACT	3.9E-1
764. bta-miR-2285h MIMAT0024583	2214	-7.20	TCTTGCATTTGCATGGGTTTTA<br>||:| | |||||| GTATTTCAAGCAAAGCCAAAAG	2.61E-1
765. bta-miR-2285af MIMAT0031057	3431	-9.10	GCTGGTTGCGATCAGTTTTGGC<br>:|:| ||||||| TTGGACTTGTT-CGAAAAACCG	1.22E-2
766. bta-miR-2285cx MIMAT0046704	3492	-15.10	TTTGCAGGATC-TTTGGGTGTT<br>|:| || |||||||||| AGTTTTTCAAGTAAACCCACAA	2.58E-1
767. bta-miR-2320-3p MIMAT0011845	3906	-15.30	TAAACAAATTGATGATCATCGT<br>||||| ||: ||||||| TTTTGTGTCCCTGGTAGTAGCT	4.64E-2
768. bta-miR-1306 MIMAT0009974	6788	-12.90	GTGTGGTTGC--TAGAGGTGC<br>| :| |||| |||||| CCTGCAAACGTCCCCTCCACC	3.52E-1
769. bta-miR-96 MIMAT0009388	8176	-13.80	AGTTGTAATAACTTGGTGCCAAC<br>||: ||| :|:||||||| TCGTTTTTACACGATCACGGTTT	3.62E-1
770. bta-miR-2285af MIMAT0031057	8738	-11.10	TAGTAGACCAGG-ATTTTGGC<br>|| ||:| ||||||| TTGGACTTGTTCGAAAAACCG	3.52E-1

1. miR Name	leftmost position of predicted target site	folding energy (in -Kcal/mol)	heteroduplex	p value
771. bta-miR-2294 MIMAT0011802	9261	-11.70	TAAAGGT-TTAGC-ACAGCCTG<br>||:| :: | ||||||| TCGTCTACGGGAGATGTCGGAG	2.28E-1
772. bta-miR-2285af MIMAT0031057	9614	-11.40	TTTCAAATCATGCTTTTTGGG<br>|| ||||||||| TTGGACTTGTTCGAAAAACCG	6.88E-2
773. bta-miR-324 MIMAT0009285	10488	-14.30	TTTTTATGGTCCTTATAAGGATGCT<br>|:||| |||||| TGTGGTTACGGGA--TCCCCTACGC	8.51E-2
774. bta-miR-383 MIMAT0009309	10666	-20.30	AGCCA-AGTTA-AGTCTGATCT<br>||||| |:|:| |||||||| TCGGTGTTAGTGGAAGACTAGA	3.07E-1
775. bta-miR-543 MIMAT0009344	11422	-17.50	AAGTGGGTTGCCGTGAATGTTT<br>:|||:|||||||| TTCTTCACGTGGCGCTTACAAA	8.26E-2
776. bta-miR-1306 MIMAT0009974	11672	-13.40	TTTTG--GGTATTGGAGGTGT<br>:| |:| ||||||| CCTGCAAACGTCCCCTCCACC	3.07E-1
777. bta-miR-12002b MIMAT0046694	12482	-10.50	CAAATAAGCAGTTGCATGAGA<br>||| : |||||| TCGATTTCTAAGGTGTACTCC	2.14E-1
778. bta-miR-543 MIMAT0009344	12639	-6.20	TTGTAATACACCTACTCAATGTTA<br>|||| |||||| TTCTTCACGTGG--CGCTTACAAA	2.18E-1
779. bta-miR-2379 MIMAT0011925	13976	-11.20	TGACTATGTT--ATTGCAGCCC<br>||| || |||||| TTTTATAGAAGAGGTCGTCGGA	3.48E-1
780. bta-miR-12037 MIMAT0046732	14994	-10.90	AGTACTATGACTGGCAGAATG<br>|| |:||||||| TGATTTCTCT-TCTGTCTTAC	9.48E-2
781. bta-miR-543 MIMAT0009344	14995	-10.20	GTACTATG-ACTGGCAGAATGTTT<br>|| ||:| |||||||| TTCTTCACGTGGC--GCTTACAAA	9.48E-2
782. bta-miR-543 MIMAT0009344	15010	-5.80	GAATGTTTCATCAAAAATGTTT<br>||:| ||||||| TTCTTCACGTGGCGCTTACAAA	9.48E-2
783. bta-miR-543 MIMAT0009344	17749	-11.80	AAACAGCGCATTCTGTAAATGTTA<br>|| ||| |:| |||||| TTCTTCACGT--GGCGCTTACAAA	1.09E-1
784. bta-miR-2285af MIMAT0031057	19029	-5.10	GGTTTGTGTA--TGTTTTGGA<br>:: || :| |||||| TTGGACTTGTTCGAAAAACCG	2.42E-1

<table>
<tr><th>1. miR Name</th><th>leftmost position of predicted target site</th><th>folding energy (in - Kcal/mol)</th><th>heteroduplex</th><th>p value</th></tr>
<tr><td>785. bta-miR-2294 MIMAT0011802</td><td>20019</td><td>-19.00</td><td>ATCTTCAGCCAATTCGACAGCCTG
       ||| || |||||||
TCGTCTACGG--GAGATGTCGGAG</td><td>7.09E-2</td></tr>
<tr><td>786. bta-miR-2285bp MIMAT0046633</td><td>20278</td><td>-5.30</td><td>ACCAGAAGATTATTGGTGGTTTG
|| :||| || ||||||
TGTGTTTC-AAGCAAGACCAAAA</td><td>1.95E-1</td></tr>
<tr><td>787. bta-miR-200a MIMAT0003822</td><td>20407</td><td>-6.80</td><td>AGAGTGGT-GGTAGTAAGAGTGTTT
   :| || : :|| | ||||||
TTGTAGCAATGGTC--TGTCACAAT</td><td>2.21E-1</td></tr>
<tr><td>788. bta-miR-12037 MIMAT0046732</td><td>21114</td><td>-9.70</td><td>TTTTCTTGGAATGCAGAATT
       :||| :||||||
TGATTTCTCTTCTGTCTTAC</td><td>3.16E-1</td></tr>
<tr><td>789. bta-miR-2285h MIMAT0024583</td><td>21153</td><td>-10.80</td><td>TTTGCATTTTG-GACGGTTTTC
       ||:| ||||||||
GTATTTCAAGCAAAGCCAAAAG</td><td>2.01E-1</td></tr>
<tr><td>790. bta-miR-205 MIMAT0003545</td><td>21186</td><td>-12.00</td><td>AATGCTTC-TTCTAGTGAAGGG
   :||:| |:||||||
GTCTGAGGCCACCTTACTTCCT</td><td>2.01E-1</td></tr>
<tr><td>791. bta-miR-2294 MIMAT0011802</td><td>21655</td><td>-12.90</td><td>TTGCTATGCTGGATATTCAGCCTG
     ||||: ||||||
TCGTCTACGG--GAGATGTCGGAG</td><td>2.25E-1</td></tr>
<tr><td>792. bta-miR-2285bp MIMAT0046633</td><td>22027</td><td>-7.30</td><td>CTGAAAAGAAAG--ATGGTTTT
    |||| | |||||||
TGTGTTTCAAGCAAGACCAAAA</td><td>6.99E-2</td></tr>
<tr><td>793. bta-miR-127 MIMAT0003787</td><td>23497</td><td>-8.60</td><td>ACCTATTTGT-GTGTATGATCCGC
  |:| |: | ||||||
TCGGT--TCGAGTCTGCCTAGGCT</td><td>3.48E-1</td></tr>
<tr><td>794. bta-miR-543 MIMAT0009344</td><td>24934</td><td>-11.30</td><td>AATTTACCTGCTGCTAATGTTT
         :|:|| |||||||
TTCTTCACGTGGCGCTTACAAA</td><td>9.48E-2</td></tr>
<tr><td>795. bta-miR-2285bp MIMAT0046633</td><td>25627</td><td>-6.20</td><td>TCTAATGGTAATCTCTATGGTTTT
      :|| || |||||||
TGTGTTTCA--AGCAAGACCAAAA</td><td>2.65E-1</td></tr>
<tr><td>796. bta-miR-12001 MIMAT0046660</td><td>26142</td><td>-9.60</td><td>TGCAGCATG--TAAATCACAGT
| ||| || ||||||
AGGTCCCACTTCGGGAGTGTCC</td><td>1.78E-1</td></tr>
<tr><td>797. bta-miR-2285bp MIMAT0046633</td><td>27371</td><td>-10.40</td><td>AGGAAGAGTTGGATCAATGGTTTA
    |:|||| | || ||||||
TGTGTTTCAAGCAAG--ACCAAAA</td><td>1.61E-2</td></tr>
<tr><td>798. bta-miR-2337 MIMAT0011870</td><td>27384</td><td>-7.40</td><td>TCAATGGTTTAA-AAACCAA
     || ||| ||||||
TTTTTCCTTATTCTTTGGTG</td><td>1.61E-2</td></tr>
</table>

1. miR Name	leftmost position of predicted target site	folding energy (in - Kcal/mol)	heteroduplex	p value
799. bta-miR-2337 MIMAT0011870	28635	-9.20	TTACAACGATGTAAAACCAC<br>:||: ||||||| TTTTTCCTTATTCTTTGGTG	1.78E-1
800. bta-miR-2337 MIMAT0011870	29492	-5.30	ATCTAGAAATGTTCAAACCAG<br>|| |||: |||||| TTTTTCCTTAT-TCTTTGGTG	7.91E-3
801. bta-miR-2337 MIMAT0011870	30557	-8.50	GAATATGAGTCCAAAACCAC<br>||:| ||||||| TTTTTCCTTATTCTTTGGTG	3.66E-2
802. bta-miR-760-3p MIMAT0022951	30947	-18.40	TGCCCAAGGG--AAGAGCCA<br>| |||| :|: |||||| AGGGGTGTCTGGGTCTCGGC	6.29E-2
803. bta-miR-665 MIMAT0009363	1681	-15.00	GACGGT-TTGGTTTATACTGGA<br>||: |:||: |||||| TCCCCGGAGCCG--GATGACCA	3.11E-2
804. bta-miR-665 MIMAT0009363	3640	-11.00	CAGTGTGTTG-CATACTGGA<br>|: |:| | |||||| TCCCCGGAGCCGGATGACCA	3.34E-1
805. bta-miR-448 MIMAT0009319	5490	-9.60	TGTGGACG-CT--GTTATGCAT<br>|||: |: |||||| TACCCTGTAGGATGTATACGTT	2.86E-1
806. bta-miR-2285cq MIMAT0046688	5795	-12.00	TTAAAAATTTGAAACAAACTTTT<br>|||||:| ||| ||||||||| TCTTTTTGACCTT-GTTTGAAAA	2.39E-2
807. bta-miR-2285av MIMAT0046347	5797	-10.90	AAAAATTTGAAACAAACTTTT<br>|||||:::||| ||||||| TTTTTGGGCTT-GCTTGAAAA	2.39E-2
808. bta-miR-758 MIMAT0009370	5867	-11.90	ATAAACCTGACT-TGTCACAAT<br>|||: ||||||| CCAATCACCTGGTCCAGTGTTT	6.48E-2
809. bta-miR-3613b MIMAT0029948	7492	-7.10	GGTGGCACT--GGCTTTGTTC<br>|| ||| |||||||| CAAC-ATGAAAAAAAAAACAAG	2.95E-2
810. bta-miR-2285av MIMAT0046347	7547	-6.20	ATAAACCAGGTAATACTTTT<br>||| |: |||||| TTTTTGGGCTTGCTTGAAAA	2.94E-1
811. bta-miR-448 MIMAT0009319	7771	-7.00	AAGAGTGTGCCTA-ATATGCAT<br>||| ||||||| TACCCTGTAGGATGTATACGTT	8.64E-2
812. bta-miR-1271 MIMAT0009975	8177	-17.10	GTTGTAATAACTTGGTGCCAAC<br>||| ||| |||||||| ACTCATGAATGATCCACGGTTC	3.62E-1

<table>
<tr><th>1. miR Name</th><th>leftmost position of predicted target site</th><th>folding energy (in -Kcal/mol)</th><th>heteroduplex</th><th>p value</th></tr>
<tr><td>813. bta-miR-2284aa
MIMAT0025560</td><td>9611</td><td>-7.80</td><td>TTGTTTCAAATCATGCTTTTTG
    ::|||| || :||||||
TTTTGGGTTT-GTTTGAAAAAA</td><td>6.88E-2</td></tr>
<tr><td>814. bta-miR-448
MIMAT0009319</td><td>10415</td><td>-9.40</td><td>GTGTTAAATTTGTGTATATGCAT
:|| | |: |::|||||||
TACCCT-GTAGGATGTATACGTT</td><td>3.24E-2</td></tr>
<tr><td>815. bta-miR-2285q
MIMAT0025574</td><td>10478</td><td>-13.80</td><td>TCAATGGGGATTTTTATGGTCCTT
:|| ::| ||:| |||||||
GGTCTTTCAAGTAAGT-CCAGGAA</td><td>8.51E-2</td></tr>
<tr><td>816. bta-miR-1271
MIMAT0009975</td><td>11675</td><td>-18.70</td><td>TGGGTATT-GGAGGTGTGCCAAT
||:|||:| :| |||||||
ACTCATGAATGATC-CACGGTTC</td><td>3.07E-1</td></tr>
<tr><td>817. bta-miR-216b
MIMAT0009266</td><td>16552</td><td>-6.63</td><td>CAGCA-ACAATACAAGAGATTG
  :|| |||||||
AGTGTAAACGGACGTCTCTAAA</td><td>2.36E-2</td></tr>
<tr><td>818. bta-miR-29a
MIMAT0003518</td><td>17101</td><td>-16.80</td><td>CTGCCAAGGTCAGGGTGGAGTGCTAT
  :|| | ||||: | ||||||
ATTGGCTAAAGTCT--AC-CACGATC</td><td>3.76E-1</td></tr>
<tr><td>819. bta-miR-2285ay
MIMAT0046355</td><td>21549</td><td>-10.09</td><td>CCACTTACCCAGTTTGAGGGTTT
||| :||: ||||||
GGTTTTCCAAGTAAG-TCCCAAA</td><td>1.45E-1</td></tr>
<tr><td>820. bta-miR-2285ce
MIMAT0046657</td><td>22027</td><td>-7.44</td><td>CTGAAAAG--AAAGATGGTTTTT
  :||||| ||||||
AGTTTTTCAAGTAAGTCCAAAAG</td><td>6.99E-2</td></tr>
<tr><td>821. bta-miR-6519
MIMAT0025537</td><td>24930</td><td>-16.70</td><td>TTATAATTTACCTGCTGCTA
          ||:|||||||
AAAGAGACAAGGGCGACGAT</td><td>9.48E-2</td></tr>
<tr><td>822. bta-miR-2285ce
MIMAT0046657</td><td>25627</td><td>-5.70</td><td>TCTAATGGTAATCTCTATGGTTTTA
|| :|| || ||||||
AGTTTTTCA--AGTAAGTCCAAAAG</td><td>2.65E-1</td></tr>
<tr><td>823. bta-miR-2285av
MIMAT0046347</td><td>25659</td><td>-7.70</td><td>CTTAAC--AAACAGAACTTTT
   ||| ||| ||||||||
TTTTTGGGCTTG-CTTGAAAA</td><td>7.54E-2</td></tr>
<tr><td>824. bta-miR-181d
MIMAT0009243</td><td>26996</td><td>-9.30</td><td>CTATGGAGAAGGTTAATGAATGTG
       || :|||||||||
TGGGTGGCT-GTTGTTACTTACAA</td><td>1.75E-1</td></tr>
<tr><td>825. bta-miR-2285bb
MIMAT0046375</td><td>28048</td><td>-12.10</td><td>AGGTAGACCTTA--TAACTTTA
||: |:|||::| ||||||
TCTTTTTGGGGTTGTTTGAAAG</td><td>2.5E-1</td></tr>
<tr><td>826. bta-miR-2285av
MIMAT0046347</td><td>28050</td><td>-6.80</td><td>GTAGACCT-TAT-AACTTTA
  |:|||: |: ||||||
TTTTTGGGCTTGCTTGAAAA</td><td>2.5E-1</td></tr>
</table>

<table>
<tr><th>1. miR Name</th><th>leftmost position of predicted target site</th><th>folding energy (in - Kcal/mol)</th><th>heteroduplex</th><th>p value</th></tr>
<tr><td>827. bta-miR-1271 MIMAT0009975</td><td>28064</td><td>-10.70</td><td>TTTAAGCATTATTAATTGCCAAA
     :| |||:|| ||||||
ACTCATG-AATGATCCACGGTTC</td><td>2.5E-1</td></tr>
<tr><td>828. bta-miR-2285bb MIMAT0046375</td><td>29371</td><td>-9.10</td><td>TGAGAAATAATATCTAAACTTTA
 ||:|||: :| :|||||||
TCTTTTTGGGGT-TGTTTGAAAG</td><td>3.85E-1</td></tr>
<tr><td>829. bta-miR-2285cq MIMAT0046688</td><td>29371</td><td>-7.30</td><td>TGAGAAATAATATCTAAACTTTA
 ||:|||:   :|||||||
TCTTTTTG-ACCTTGTTTGAAAA</td><td>3.85E-1</td></tr>
<tr><td>830. bta-miR-2390 MIMAT0011943</td><td>29690</td><td>-10.70</td><td>GGGGTACTGGTACAGACACAAC
||:: || || |||||||
CCTTTTGTTTTTG--TGTGTTG</td><td>2.01E-1</td></tr>
<tr><td>831. bta-miR-28 MIMAT0009272</td><td>29966</td><td>-17.20</td><td>CTCA--GGAAGGTCTGCTCCTA
|||| :|| || ||||||
GAGTTATCTGACACTCGAGGAA</td><td>2.01E-1</td></tr>
<tr><td>832. bta-miR-204 MIMAT0004338</td><td>30937</td><td>-9.80</td><td>GCCGAACAAGTGCCCAAGGGAA
         :|| | |||||||
TCCGTATCCTACTGTTTCCCTT</td><td>6.29E-2</td></tr>
<tr><td>833. bta-miR-2285ao MIMAT0046360</td><td>2212</td><td>-12.50</td><td>GCTCTTGCATTTGCATGGGTTT
||: ||:| |||||||
CGGTTTTTCAAGCAGACCCAAA</td><td>2.61E-1</td></tr>
<tr><td>834. bta-miR-2285df MIMAT0046729</td><td>2214</td><td>-8.40</td><td>TCTTGCATTTGCATGGGTTTTA
       |:|| ::||||||
GGTTTTCAGACAAGTCCAAAAA</td><td>2.61E-1</td></tr>
<tr><td>835. bta-miR-12039 MIMAT0046734</td><td>3453</td><td>-12.90</td><td>AGTGATGCAGAAACTGCCCTGT
|| || | || ||||||
TCTCTTCTTCGGTCCCGGGACC</td><td>1.22E-2</td></tr>
<tr><td>836. bta-miR-329a MIMAT0009288</td><td>3625</td><td>-11.20</td><td>ACCTTATGTGATTGGCAGTGTGTT
         :|: | |||||||
TTTTTCCAATTGGTC--CACACAA</td><td>3.34E-1</td></tr>
<tr><td>837. bta-miR-10b MIMAT0003839</td><td>4284</td><td>-13.40</td><td>ACCTGATGCGAGGAC-ACAGGGTA
    :|| || | | ||||||||
GTGTTTAAGC-CAAGATGTCCCAT</td><td>1.75E-1</td></tr>
<tr><td>838. bta-miR-140 MIMAT0003789</td><td>7779</td><td>-10.50</td><td>GCCTAATATGCATGTTGTGGTA
           |: :|||||||
AGGCACCAAGATGGGACACCAT</td><td>8.64E-2</td></tr>
<tr><td>839. bta-miR-2480 MIMAT0012072</td><td>10531</td><td>-6.70</td><td>GTT-CAGGATTATATACAAT
||: |:|| |||||||
CAGTATTCTTTGTTATGTTA</td><td>8.51E-2</td></tr>
<tr><td>840. bta-let-7c MIMAT0004332</td><td>11441</td><td>-10.90</td><td>TTTTGTATTTCACAGATATACCTCA
  : ||: || |||||||
TTGGTATG--TTG-GATGATGGAGT</td><td>8.26E-2</td></tr>
</table>

<table>
<tr><th>1. miR Name</th><th>leftmost position of predicted target site</th><th>folding energy (in -Kcal/mol)</th><th>heteroduplex</th><th>p value</th></tr>
<tr><td>841. bta-miR-376c MIMAT0009947</td><td>12447</td><td>-6.77</td><td>ATGGCAGATTCAAACTATCCAA
  ::|| ||||||
ATTTGTATCTTCCTTATAGGTG</td><td>2.14E-1</td></tr>
<tr><td>842. bta-miR-2471-3p MIMAT0012062</td><td>16143</td><td>-15.10</td><td>CGTTGTGGCAGTT-GCATCAGA
 |: | ||| ||: |||||||
TCGTC-CCGACAGAAGTAGTCT</td><td>5.43E-2</td></tr>
<tr><td>843. bta-miR-2480 MIMAT0012072</td><td>16547</td><td>-11.60</td><td>TGCATCAGCAACAATACAAG
  ||| || ||||||||||
CAGTATTCTTTGTTATGTTA</td><td>2.36E-2</td></tr>
<tr><td>844. bta-miR-2480 MIMAT0012072</td><td>17045</td><td>-5.80</td><td>ATTGTGTGAAAAAGCATACAAA
 | |: |||| ||||||
CAGTATTCTTT--GTTATGTTA</td><td>2.54E-1</td></tr>
<tr><td>845. bta-miR-329a MIMAT0009288</td><td>17308</td><td>-13.50</td><td>AATTGCCAGCACCACGTGTGTT
           |||| |||||||
TTTTTCCAATTGGTCCACACAA</td><td>3.52E-1</td></tr>
<tr><td>846. bta-miR-2418 MIMAT0011982</td><td>18337</td><td>-15.10</td><td>AACAATTTAAGCATCTCATCCCT
          :|| ||||||||
GCTGGTGCGGTGT-GAGTAGGGT</td><td>3.39E-1</td></tr>
<tr><td>847. bta-miR-2310 MIMAT0011822</td><td>19875</td><td>-10.60</td><td>GTTTACATTTCCACGACAAAAG
    ||| :||| :||||||
TCTTTGTTTGGGT-TTGTTTTG</td><td>3.48E-1</td></tr>
<tr><td>848. bta-miR-2285df MIMAT0046729</td><td>21153</td><td>-6.20</td><td>TTTGCATTTTGGAC-GGTTTTC
       |:|| | ||||||
GGTTTTCAGACAAGTCCAAAAA</td><td>2.01E-1</td></tr>
<tr><td>849. bta-miR-2285df MIMAT0046729</td><td>21188</td><td>-9.60</td><td>TGCTTCTTCTAGTGAAGGGTTTTT
       ||| || :|||||||
GGTTTTCAGA-CA-AGTCCAAAAA</td><td>2.01E-1</td></tr>
<tr><td>850. bta-miR-2285df MIMAT0046729</td><td>22028</td><td>-6.14</td><td>TGAAAAG--AAAGATGGTTTTT
  ||||| ||||||
GGTTTTCAGACAAGTCCAAAAA</td><td>6.99E-2</td></tr>
<tr><td>851. bta-miR-2285ao MIMAT0046360</td><td>22109</td><td>-10.31</td><td>GCTAG-CTATGATTATGGGTTA
||:|: ||||||
CGGTTTTTCAAGCAGACCCAAA</td><td>3.48E-1</td></tr>
<tr><td>852. bta-miR-12039 MIMAT0046734</td><td>22525</td><td>-11.84</td><td>GGACCTTAATCC--TGCCCTGT
:|| || ||||||
TCTCTTCTTCGGTCCCGGGACC</td><td>3.66E-1</td></tr>
<tr><td>853. bta-miR-2292 MIMAT0011800</td><td>24731</td><td>-19.80</td><td>TGTCTTTTATTCAG--GCAGACTC
||:|||: :|||: |||||||
ACGGAAG--GAGTTAGAGTCTGAG</td><td>1.75E-1</td></tr>
<tr><td>854. bta-miR-15a MIMAT0004334</td><td>24760</td><td>-12.10</td><td>TGTAATAATATTGATGCTGCTA
   ||: || ||||||||
TGTTTGGTAATAC-ACGACGAT</td><td>1.75E-1</td></tr>
</table>

1. miR Name	leftmost position of predicted target site	folding energy (in - Kcal/mol)	heteroduplex	p value
855. bta-miR-2285ah-5p MIMAT0040945	25271	-21.70	CAGGGCCTTACAAGTGCCCCCAA<br>|||: || |:||||||| AGGACGGG-TGGAGATGGGGGTT	1.04E-1
856. bta-miR-2285df MIMAT0046729	25628	-8.70	CTAATGGTAATCTCTATGGTTTTA<br>|:|| :|| || |||||| GGTTTTCAGACAAG--TCCAAAAA	2.65E-1
857. bta-miR-2284f MIMAT0011905	25656	-6.00	CTACTTAACAAACAGAACTTT<br>||| ||||||| CTTTTTAGGCTTG-CTTGAAA	7.54E-2
858. bta-miR-2418 MIMAT0011982	27754	-15.10	TCCTGACGATATATTACATCCCT<br>||| :|:|:| |||||| GCTGGTGCGGTGTGA-GTAGGGT	2.11E-1
859. bta-miR-320b MIMAT0011991	29660	-17.82	TGCACC-AGGAGTCCCAGCT<br>|||| ||||||| TGGTGGGAGAGTTGGGTCGA	2.69E-1
860. bta-let-7c MIMAT0004332	30054	-15.00	GGCAACAGAACC-CCTACCTCT<br>| |||| ||||||| TTGGTATGTTGGATGATGGAGT	2.94E-1
861. bta-miR-11995 MIMAT0046648	30666	-13.30	AGAGAGTTGACTGCAGAGGACAT<br>||:||| |||||| TCTGTCGACT-CTTCATCCTGTT	1.28E-1
862. bta-miR-12018 MIMAT0046701	397	-12.40	CGCCGAC-TTCTTAAACAAGAG<br>||| ||||: |||||| AGTTCTGTAAGAGCCTGTTCTA	1.45E-1
863. bta-miR-2297 MIMAT0011805	400	-7.30	CGACTTCTTAAACAAGAGTG<br>||| ||||||| TCCTTATTCTTTTTTCTCAC	1.45E-1
864. bta-miR-17-3p MIMAT0003816	1122	-10.40	ACGTTATGTTATGAGACTGCAGA<br>||: ||:: ||||||| TGT-TCACGG--AAGTGACGTCA	1.69E-1
865. bta-miR-2285bv MIMAT0046642	2873	-10.60	ACACTGCATGT-GGAGTGTTTG<br>|| ||| |||||||| TTTTACAAACAAGCTCACAAAA	1.36E-1
866. bta-miR-2285cb MIMAT0046651	3493	-14.40	TTGCAGGATC-TTTGGGTGTT<br>|:| || |::||||||| GGTTTTCAAGTAGGCCCACAA	2.58E-1
867. bta-miR-655 MIMAT0009360	5904	-5.20	TAAGTATTATACTCAGCGTATTAT<br>|| || ||||||| TCTCTCCAATTG-GT-ACATAATA	6.48E-2
868. bta-miR-2455 MIMAT0012037	6905	-12.10	CTTTTGTTGGTAAGATCGCACAGT<br>:::||:: : || |||||| AGGGACGGAGGGGCT--CGTGTCT	2.28E-1

1. miR Name	leftmost position of predicted target site	folding energy (in - Kcal/mol)	heteroduplex	p value
869. bta-miR-1721 MIMAT0011849	9587	-7.80	TTTGTTTGCTATATATATCTGTT<br>:|:|| :|||||| AGAGTCTTAGTGTA-GTAGACAC	6.88E-2
870. bta-miR-17-3p MIMAT0003816	9632	-11.90	GGGTATTTTCTTACTGCAGA<br>: |:||||||| TGTTCACGGAAGTGACGTCA	2.73E-1
871. bta-miR-2437 MIMAT0012011	10296	-11.90	TGCTTATAACGGCAAACCACA<br>|||:: |||||||| TTTCCTTTTGTTTTTTGGTGT	3.62E-1
872. bta-miR-2285cd MIMAT0046655	10498	-15.10	CCTTATAAGGATGCTCAGGTTGTC<br>|| ||| :: |||||||| GG-TTTTTCAAGTAGGTCCAACAA	8.51E-2
873. bta-miR-1721 MIMAT0011849	10531	-19.80	GTTCAGGATTATATACAATCTGTT<br>:||||:||:|:|| |||||| AGAGTCTTAGTGTA--GTAGACAC	8.51E-2
874. bta-miR-6119-5p MIMAT0024588	11443	-7.50	TTGTATTTCACAGATATACCTCA<br>:| ||| |||||| TCAGTTTAGTTAAAAAATGGAGA	8.26E-2
875. bta-miR-2285cd MIMAT0046655	12827	-9.60	ACCTTTATTTTGTAAAAGGTTGTA<br>|| | ||||||| GGTTTTTCAAGTA-GGTCCAACAA	3.34E-1
876. bta-miR-2285cd MIMAT0046655	13751	-8.30	TCTCTCTATATATGCTGGTTGTG<br>:|| | |||||| GGTTTTTCAAGTAGGTCCAACAA	1.32E-1
877. bta-miR-532 MIMAT0003848	13905	-11.70	GTGGAGGTAGGCTTAGTAGGCATT<br>|| :||:| |||||| TGCCAGGATGTGAGT--TCCGTAC	3.29E-1
878. bta-miR-2285cb MIMAT0046651	13979	-10.54	CTATGTTATTGCAGCCCCAGGGTGTG<br>|:| || || |||||| GGT--TTTCAAGT--AGG-CCCACAA	3.48E-1
879. bta-miR-12003 MIMAT0046662	15735	-17.10	GAATTCTGTTCACAACATACAATGCT<br>|||: ||:| |||| ||||||| CTTG-GATACCGGTTG--GGTTACGA	1.15E-2
880. bta-miR-628 MIMAT0009356	16537	-8.40	AGCAGAGT-TATGCATCAGCAA<br>||| ||: |||||| GGAGATCATTTATACAGTCGTA	2.36E-2
881. bta-miR-30c MIMAT0003850	17150	-6.70	CACTCGTAAGTA-TGTGTTTACT<br>||| :||||||| CGACTCTCACATCCTACAAATGT	3.39E-1
882. bta-miR-582 MIMAT0009351	19033	-9.20	TGTG-TATGTTTTGGAACTGTAA<br>||: ||| : |||||||| TCATTGACCAACTTGTTGACATT	2.42E-1

<table>
<tr><th>1. miR Name</th><th>leftmost position of predicted target site</th><th>folding energy (in - Kcal/mol)</th><th>heteroduplex</th><th>p value</th></tr>
<tr><td>883. bta-miR-2285bn MIMAT0046625</td><td>19389</td><td>-8.91</td><td>ACAG-CTACTACAGCAGGTTTT
|||: ||||||||
TGTTTTTCAAGTAAGTCCAAAA</td><td>3.12E-1</td></tr>
<tr><td>884. bta-miR-190b MIMAT0009252</td><td>19600</td><td>-7.70</td><td>TTATTAATAATACAACATATCC
   ::|||| :| ||||||||
TTGGGTTATAGT-TTGTATAGT</td><td>5.58E-3</td></tr>
<tr><td>885. bta-miR-2297 MIMAT0011805</td><td>20406</td><td>-10.70</td><td>AAGAGTGGTGGTAGTAAGAGTG
  ||:|:: |: |||||||
TCCTTATT-CT-TTTTTCTCAC</td><td>2.21E-1</td></tr>
<tr><td>886. bta-miR-2285bv MIMAT0046642</td><td>20410</td><td>-12.00</td><td>GTGGTGGTAGTAAGAGTGTTTG
  ::|| || ||||||||
TTTTACAAACAAGCTCACAAAA</td><td>2.21E-1</td></tr>
<tr><td>887. bta-miR-148b MIMAT0003814</td><td>20414</td><td>-12.60</td><td>TGGTAGTAAGAGTGTTTGCACTGT
    ||| ||| |||||||
TGTTTCA-AGACAC-TACGTGACT</td><td>2.21E-1</td></tr>
<tr><td>888. bta-miR-12003 MIMAT0046662</td><td>23218</td><td>-11.80</td><td>TGATTCACGGTGGA-ACAATGCC
        ||: :| ||||||
CTTGGATACCGGTTGGGTTACGA</td><td>1.89E-1</td></tr>
<tr><td>889. bta-miR-2285bv MIMAT0046642</td><td>25024</td><td>-6.40</td><td>CCTCAACCTGT--AGGTGTTTT
        ||| :|||||||
TTTTACAAACAAGCTCACAAAA</td><td>2.46E-1</td></tr>
<tr><td>890. bta-miR-12003 MIMAT0046662</td><td>26689</td><td>-10.30</td><td>ATTGCTAATGCATTTAACAATGCT
    ||| || |||||||
CTTGGATACCG-GTTGGGTTACGA</td><td>1.72E-1</td></tr>
<tr><td>891. bta-miR-12018 MIMAT0046701</td><td>26839</td><td>-9.60</td><td>ATAAGTTCTTCTT-TACAAGAA
 :||| ||||: ||||||
AGTTCTGTAAGAGCCTGTTCTA</td><td>3.25E-1</td></tr>
<tr><td>892. bta-miR-2297 MIMAT0011805</td><td>27361</td><td>-7.90</td><td>CATGATTTTAAGGAAGAGTT
         ||::||||||
TCCTTATTCTTTTTTCTCAC</td><td>1.61E-2</td></tr>
<tr><td>893. bta-miR-12003 MIMAT0046662</td><td>27821</td><td>-13.20</td><td>ATTATAATAGCTA-CCACAATGCC
         ||:| || ||||||
CTTGGATACCGGTTGG-GTTACGA</td><td>2.25E-1</td></tr>
<tr><td>894. bta-miR-2437 MIMAT0012011</td><td>28635</td><td>-8.60</td><td>TTACAACGATGTAAAACCACC
       :|:: ||||||||
TTTCCTTTTGTTTTTTGGTGT</td><td>1.78E-1</td></tr>
<tr><td>895. bta-miR-2374 MIMAT0011920</td><td>30205</td><td>-25.36</td><td>CCCGCCAGAAGAGGAGCCCCAA
|||||| ||||||||
GGGCGG-AGAGGGGTCGGGGTT</td><td>5.91E-3</td></tr>
<tr><td>896. bta-miR-12018 MIMAT0046701</td><td>30526</td><td>-7.90</td><td>TGAATGCATATCAACAACAAGAT
     :||| || |||||||
AGTTCTGTA-AGAGCCTGTTCTA</td><td>2.11E-1</td></tr>
</table>

<table>
<tr><th>1. miR Name</th><th>leftmost position of predicted target site</th><th>folding energy (in -Kcal/mol)</th><th>heteroduplex</th><th>p value</th></tr>
<tr><td>897. bta-miR-17-3p MIMAT0003816</td><td>30663</td><td>-11.60</td><td>AGTAGAGAGTTGACTGCAGA
      | || |||||||
TGTTCACGGAAGTGACGTCA</td><td>1.28E-1</td></tr>
<tr><td>898. bta-miR-6529b MIMAT0029947</td><td>266</td><td>-17.80</td><td>GGATGTTTGAGGACGCAGAGG
        |||| |||||||
TCTCTAGTCTCC-GCGTCTCA</td><td>3.03E-1</td></tr>
<tr><td>899. bta-miR-29e MIMAT0009953</td><td>479</td><td>-10.90</td><td>AGGTGCT-ACTTCAA--GATGCTT
  : || ||||| ||||||
TTTGTGACTAAAGTTTACTACGAT</td><td>3.03E-1</td></tr>
<tr><td>900. bta-miR-6529b MIMAT0029947</td><td>1125</td><td>-14.10</td><td>TTATGTTATGAGACTGCAGAGT
    :|:| ||| :|||||||
TCTCTAGT-CTC-CGCGTCTCA</td><td>1.69E-1</td></tr>
<tr><td>901. bta-miR-2469 MIMAT0012059</td><td>2783</td><td>-15.30</td><td>AGG-AACAG--CCTACAGTAA
|:| ||| ||||||||||
TTCGGCGTCCGGGATGTCATT</td><td>2.9E-1</td></tr>
<tr><td>902. bta-miR-2284k MIMAT0011907</td><td>3982</td><td>-6.20</td><td>TTTTCAATGACT--ACTTTTG
       :|||: ||||||
TTTTTGGGCTGGCTTGAAAAG</td><td>2.11E-1</td></tr>
<tr><td>903. bta-miR-2284k MIMAT0011907</td><td>5797</td><td>-9.60</td><td>AAAAATTTGAAACAAACTTTTA
|||||:::|| |||||||
TTTTTGGGCT-GGCTTGAAAAG</td><td>2.39E-2</td></tr>
<tr><td>904. bta-miR-2469 MIMAT0012059</td><td>7067</td><td>-13.80</td><td>AAGCCATTGATGTAGTACAGTAT
||||| |: |||||||
TTCGG--CGTCCGGGATGTCATT</td><td>2.42E-1</td></tr>
<tr><td>905. bta-miR-2285bs MIMAT0046637</td><td>7749</td><td>-8.20</td><td>TAATTTGCTACATTCTAAGGTTAA
          |||:| ||||||
GGTTTTTCAAGTAGG--TCCAATC</td><td>2.98E-1</td></tr>
<tr><td>906. bta-miR-767 MIMAT0009375</td><td>8174</td><td>-13.10</td><td>AAAGTTGTAATAACT-TGGTGCC
   |:| |:|||: ||||||
GTACGAGTCTGTTGGTACCACGT</td><td>3.62E-1</td></tr>
<tr><td>907. bta-miR-484 MIMAT0003535</td><td>9258</td><td>-10.00</td><td>GTTTAAAGGTTTAGCACAGCCTGT
      ||| :| ||||||
TAGCCCTCC--CCTGACTCGGACT</td><td>2.28E-1</td></tr>
<tr><td>908. bta-miR-2284k MIMAT0011907</td><td>9693</td><td>-6.00</td><td>AATGGCTCTTACT--ACTTTTA
   ::|:| ||: ||||||
TTTTTGGG-CTGGCTTGAAAAG</td><td>1.98E-1</td></tr>
<tr><td>909. bta-miR-29e MIMAT0009953</td><td>10488</td><td>-8.50</td><td>TTTTTATGGTCCTTATAAGGATGCTC
      ||:| |:| ||||||
TTTGTGACTA-AAGT-TTACTACGAT</td><td>8.51E-2</td></tr>
<tr><td>910. bta-miR-2285bs MIMAT0046637</td><td>11394</td><td>-5.80</td><td>ATCTATGGCTGCAGCAAAGGTTAT
      :| || ||||||
GGTTTTTC-AAGT-AGGTCCAATC</td><td>1.15E-2</td></tr>
</table>

1. miR Name	leftmost position of predicted target site	folding energy (in -Kcal/mol)	heteroduplex	p value
911. bta-let-7b MIMAT0004331	11446	-14.10	TATTTCACAG--ATATACCTCA<br>|: ||||: ||||||| TTGGTGTGTTGGATGATGGAGT	8.26E-2
912. bta-miR-2364 MIMAT0011902	11975	-16.80	CTGTTTTGCAGGC-TTTACAGAGT<br>|||: : || | |::|||||| GACG-GGTGTAGGTAGGTGTCTCT	2.39E-1
913. bta-miR-424-3p MIMAT0015304	13464	-8.40	AAAGTTAATTGCTGC-CGTTTTC<br>||: :||: | |||||| TATCG--TCGCGGAGTGCAAAAC	3.76E-1
914. bta-miR-6529b MIMAT0029947	16083	-15.70	AGAAGTGCAGTTATGCAGAGT<br>||| | ||| :||||||| TCTCTA-GTCTCCGCGTCTCA	3.52E-1
915. bta-miR-2285bf MIMAT0046382	16217	-6.10	TAAATATGTTT-TGAGTGTTTC<br>:||| |||| |||||| GTTT-TTCAAACAAGCACAAAA	1.69E-1
916. bta-miR-2285bf MIMAT0046382	17149	-5.80	CCACTCGTAAGTATGTGTTTA<br>|| || :|||||| GTTTTTCAAACAAGCACAAAA	3.39E-1
917. bta-miR-2477 MIMAT0012069	19151	-13.80	TGTTA-AC-AAACATGCATTCCAC<br>:||:| || ||| |||||||| GCAGTCTGAATAGTA-GTAAGGTG	3.16E-1
918. bta-miR-29e MIMAT0009953	19250	-14.70	GTATATGGATGGCA-TGGATGCTA<br>|:|: ||| || ||||||| TTTGTGACTAAAGTTTACTACGAT	2.46E-1
919. bta-miR-370 MIMAT0009300	19260	-13.20	GGCATGG--ATGCT-AAGCAGGT<br>: || || ::|: |||||| TGGT-CCAAGGTGGGGTCGTCCG	2.46E-1
920. bta-miR-484 MIMAT0003535	20024	-13.20	CAGCCAATTCGAC--AGCCTGA<br>||| ||||||| TAGCCCTCCCCTGACTCGGACT	7.09E-2
921. bta-miR-2477 MIMAT0012069	21276	-8.20	TATTTGTTTTGGAGAAATTCCAC<br>:||: ||||||| GCAGTCTGAATAGTAGTAAGGTG	7.65E-2
922. bta-miR-2469 MIMAT0012059	21460	-10.90	TTTTTGTTGGT--GACAGTAT<br>::|: ||: |||||| TTCGGCGTCCGGGATGTCATT	1.45E-1
923. bta-miR-484 MIMAT0003535	21656	-13.50	TGCTATGCTGGATATTCAGCCTGA<br>|||: ||||||| TAGCCCTCCCCTG--ACTCGGACT	2.25E-1
924. bta-miR-767 MIMAT0009375	22006	-12.40	TTGAAATAGTTAAGCT-TGGTGCT<br>:|| |:|: |||||| GTACGAGTC-TGTTGGTACCACGT	6.99E-2

<table>
<thead>
<tr><th>1. miR Name</th><th>leftmost position of predicted target site</th><th>folding energy (in -Kcal/mol)</th><th>heteroduplex</th><th>p value</th></tr>
</thead>
<tbody>
<tr><td>925. bta-miR-7862 MIMAT0030442</td><td>24531</td><td>-13.70</td><td>AACACTATCTA--TAGCACCA
||:| |||||||
CGAGTCGAGGTCCCTCGTGGT</td><td>5.76E-2</td></tr>
<tr><td>926. bta-miR-29e MIMAT0009953</td><td>24753</td><td>-9.50</td><td>ATTTACTTGTAATAATATTGATGCTG
:||| :| :|| |||||||
TTTGTGACTAAAGTT--TACTACGAT</td><td>1.75E-1</td></tr>
<tr><td>927. bta-miR-2285bf MIMAT0046382</td><td>25023</td><td>-6.60</td><td>GCCTCAACCTGTAGGTGTTTT
||| |||||||
GTTTTTCAAACAAGCACAAAA</td><td>2.46E-1</td></tr>
<tr><td>928. bta-miR-11987 MIMAT0046387</td><td>26566</td><td>-23.80</td><td>ACCTCTGCTAGTCTGTTTCCTCC
||||| |:|| | ||||||
TGGAGGTGGTC--TCTAAGGAGC</td><td>3.46E-2</td></tr>
<tr><td>929. bta-miR-2285ct MIMAT0046695</td><td>28048</td><td>-7.10</td><td>AGGTAGA-CC-TTATAACTTTA
||: |:| || |||||||
TCTTTTTAGGTTTGATTGAAAG</td><td>2.5E-1</td></tr>
<tr><td>930. bta-miR-6529b MIMAT0029947</td><td>29624</td><td>-12.90</td><td>GGAG-TTTGAATTTGCAGAGG
:||| || :||||||
TCTCTAGTCT-CCGCGTCTCA</td><td>8.64E-2</td></tr>
<tr><td>931. bta-miR-2299-3p MIMAT0011808</td><td>29851</td><td>-19.30</td><td>AGGCTGATGTCAATACCCCGGC
| |||| :|| || ||||||
TGCGAC-CTAGGTAAGGGGCCT</td><td>1.98E-1</td></tr>
<tr><td>932. bta-miR-2392 MIMAT0011945</td><td>29992</td><td>-19.20</td><td>GATCTACTTCACGCGCATCCAG
|:|||| | ||||||
ACGTGGGGAGTGGGGGTAGGTA</td><td>2.01E-1</td></tr>
<tr><td>933. bta-let-7b MIMAT0004331</td><td>30054</td><td>-19.30</td><td>GGCAACAGAACC-CCTACCTCT
::| ||| |||| |||||||
TTGGTGTGTTGGATGATGGAGT</td><td>2.94E-1</td></tr>
<tr><td>934. bta-miR-2469 MIMAT0012059</td><td>30460</td><td>-8.80</td><td>GTGCAATTAGATTTGACAGTAC
|| :|| : ||||||
TTCG-GCGTCCGGGATGTCATT</td><td>3.29E-1</td></tr>
<tr><td>935. bta-miR-29d-3p MIMAT0009275</td><td>2236</td><td>-13.70</td><td>TTTCATGTTTTGCATGGTGCTT
:|||:: ||||||||
ATTAGCTAAAGTTTACCACGAT</td><td>2.61E-1</td></tr>
<tr><td>936. bta-miR-23a MIMAT0003827</td><td>3655</td><td>-17.10</td><td>TGGAAATGC-ATTAAATGTGAT
||||||| | ||||||||
ACCTTTAGGGACCGTTACACTA</td><td>3.34E-1</td></tr>
<tr><td>937. bta-miR-29d-3p MIMAT0009275</td><td>6787</td><td>-12.30</td><td>AGTGTGGTTGCTAGA-GGTGCTT
:|:|| :|:| ||||||
ATTAGCTAA-AGTTTACCACGAT</td><td>3.52E-1</td></tr>
<tr><td>938. bta-miR-11979 MIMAT0046367</td><td>8176</td><td>-9.50</td><td>AGTTGTAATAAC--TTGGTGCC
|| || |: || ||||||
TCCTCACTGGTGCCCACCACGT</td><td>3.62E-1</td></tr>
</tbody>
</table>

<table>
<tr><th>1. miR Name</th><th>leftmost position of predicted target site</th><th>folding energy (in - Kcal/mol)</th><th>heteroduplex</th><th>p value</th></tr>
<tr><td>939. bta-miR-302c MIMAT0009281</td><td>8794</td><td>-10.30</td><td>TATGGTTACCATGTGTTGCACTTT
|||| | ||||||
GGTGACTTTGTAC-CTTCGTGAAT</td><td>1.54E-1</td></tr>
<tr><td>940. bta-miR-302c MIMAT0009281</td><td>8811</td><td>-9.50</td><td>GCACTTTATTACA--CATGCACTTT
|||| ||| ||||||
GGTGA--CTTTGTACCTTCGTGAAT</td><td>1.54E-1</td></tr>
<tr><td>941. bta-miR-301a MIMAT0009276</td><td>8905</td><td>-15.80</td><td>GTGCTTT-CCTCTGCT--TGCACTA
:|||||| | |:|| ||||||
TACGAAACTGTTATGATAACGTGAC</td><td>2.69E-1</td></tr>
<tr><td>942. bta-miR-2285ak-5p MIMAT0040948</td><td>8943</td><td>-8.70</td><td>TGG-TAGTCC--AC-AACCTTA
|| | || || ||||||
TCCTTTTGGGCTTGCTTGGAAA</td><td>3.62E-1</td></tr>
<tr><td>943. bta-miR-11979 MIMAT0046367</td><td>9301</td><td>-16.70</td><td>GCTAGT-TCCATTGCTGGTGCT
||| |||: | ||||||
TCCTCACTGGTGCCCACCACGT</td><td>2.28E-1</td></tr>
<tr><td>944. bta-miR-487a MIMAT0003805</td><td>9697</td><td>-6.94</td><td>GCTCTTACTACTTTTATGATT
:|| |:| |||||||
TGACCTACAGGGACATACTAA</td><td>1.98E-1</td></tr>
<tr><td>945. bta-let-7e MIMAT0004333</td><td>11442</td><td>-11.40</td><td>TTTGTATTTCACAGATATACCTCA
:|:||: || |||||||
TGATATG--TTG-GAGGATGGAGT</td><td>8.26E-2</td></tr>
<tr><td>946. bta-miR-98 MIMAT0003809</td><td>11446</td><td>-10.70</td><td>TATTTCACAG--ATATACCTCA
|||: |||||||
TTGTTATGTTGAATGATGGAGT</td><td>8.26E-2</td></tr>
<tr><td>947. bta-miR-302c MIMAT0009281</td><td>11800</td><td>-9.90</td><td>TTGTGGCAATATTGTAGCACTTT
||:|| | |||||||
GGTGACTTTGTACCTTCGTGAAT</td><td>6.2E-2</td></tr>
<tr><td>948. bta-miR-487a MIMAT0003805</td><td>14022</td><td>-6.70</td><td>TCTTATTATTCTTATATGATG
|:|:| ||||||
TGACCTACAGGGACATACTAA</td><td>2.42E-1</td></tr>
<tr><td>949. bta-miR-1287 MIMAT0009971</td><td>14418</td><td>-23.50</td><td>CTTTTATATGCTGCTGATCCAGCT
:|:| :| ||||||||||
CTGAGT--TTGGTGACTAGGTCGT</td><td>7.54E-2</td></tr>
<tr><td>950. bta-miR-2318 MIMAT0011841</td><td>15733</td><td>-7.10</td><td>ATGAATTCTGTTCACAACATACAA
:|||| ||||||
CCAGTCTATTAAGT--AGTATGTG</td><td>1.15E-2</td></tr>
<tr><td>951. bta-miR-23a MIMAT0003827</td><td>16249</td><td>-8.60</td><td>GTAACGCACCAGG--ATGTGAT
|| || |||||||
ACCTTTAGGGACCGTTACACTA</td><td>1.36E-1</td></tr>
<tr><td>952. bta-miR-2318 MIMAT0011841</td><td>17044</td><td>-5.30</td><td>CATTGTGTGAAAAAGCATACAA
:|:| ||||||
CCAGTCTATTAAGTAGTATGTG</td><td>2.54E-1</td></tr>
</table>

<table>
<tr><th>1. miR Name</th><th>leftmost position of predicted target site</th><th>folding energy (in -Kcal/mol)</th><th>heteroduplex</th><th>p value</th></tr>
<tr><td>953. bta-miR-2285d
MIMAT0011815</td><td>19260</td><td>-10.70</td><td>GGCATGGATGCTAAGCAGGTTG
| || :|| ||||||
CGGTTTTTAAAGCAAGTCCAAA</td><td>2.46E-1</td></tr>
<tr><td>954. bta-miR-2318
MIMAT0011841</td><td>20362</td><td>-9.00</td><td>TTTCATACGACTCCAGCATACAC
  ||| | | || |||||||
CCAGTCTATTAAG-TAGTATGTG</td><td>1.89E-1</td></tr>
<tr><td>955. bta-miR-29d-3p
MIMAT0009275</td><td>21298</td><td>-12.00</td><td>CAGTTTGGAACGGG-GGTGCTT
          |::: ||||||
ATTAGCTAAAGTTTACCACGAT</td><td>7.65E-2</td></tr>
<tr><td>956. bta-miR-2285bd
MIMAT0046379</td><td>21554</td><td>-9.10</td><td>TACCCAGTTTG--AGGGTTT
     ||||:: ||||||
GTTTTTCAAGTAGTCCCAAA</td><td>1.45E-1</td></tr>
<tr><td>957. bta-miR-11979
MIMAT0046367</td><td>22010</td><td>-8.90</td><td>AATAGT-TAAGC--TTGGTGCT
   ||| :| ||||||
TCCTCACTGGTGCCCACCACGT</td><td>6.99E-2</td></tr>
<tr><td>958. bta-miR-669
MIMAT0013838</td><td>22083</td><td>-12.50</td><td>TTTGAATAT-AATCCACCCACT
     |:|| | |||||||
GTGCGTGTACGTGTGTGGGTGT</td><td>3.48E-1</td></tr>
<tr><td>959. bta-miR-487a
MIMAT0003805</td><td>22101</td><td>-8.60</td><td>ACTGATAAGCTAGCTATGATT
|||| |: |||||||
TGACCTACAGGGACATACTAA</td><td>3.48E-1</td></tr>
<tr><td>960. bta-miR-215
MIMAT0003797</td><td>22616</td><td>-7.40</td><td>AATTACACAG-GCGAAGGTCAA
       ||: |: ||||||
ACAGACAGTTAAGTATCCAGTA</td><td>1.86E-1</td></tr>
<tr><td>961. bta-miR-2288
MIMAT0011795</td><td>23290</td><td>-11.80</td><td>TTTTCGTAATTCTACTACCA
   :|::| :||||||||
TTTGGTGTGTGGATGATGGA</td><td>2.01E-1</td></tr>
<tr><td>962. bta-miR-2288
MIMAT0011795</td><td>23827</td><td>-11.80</td><td>TTGCTTAATGGTTACTACCC
  :|: |:: :|||||||
TTTGGTGTGTGGATGATGGA</td><td>2.86E-1</td></tr>
<tr><td>963. bta-miR-23a
MIMAT0003827</td><td>24228</td><td>-7.40</td><td>GCGTAATTTCACATATGATGTGAA
    ||| | :||||||
ACCTTTAGGG--ACCGTTACACTA</td><td>1.36E-1</td></tr>
<tr><td>964. bta-miR-23a
MIMAT0003827</td><td>25868</td><td>-7.00</td><td>GTGTTGTTC--AAACATGTGAT
     :|:| |||||||
ACCTTTAGGGACCGTTACACTA</td><td>1.98E-1</td></tr>
<tr><td>965. bta-miR-23a
MIMAT0003827</td><td>27766</td><td>-12.10</td><td>ATTACATCCCTTCAATCATGTGAA
     |||||| ||||||
ACCTTTAGGGA--CCGTTACACTA</td><td>2.11E-1</td></tr>
<tr><td>966. bta-miR-26b
MIMAT0003531</td><td>29046</td><td>-6.40</td><td>AACCCAGAAACAAACAACTTGAT
|||| ||||||
TTGG-ATAGGACTTAATGAACTT</td><td>3.15E-2</td></tr>
</table>

<table>
<tr><th>1. miR Name</th><th>leftmost position of predicted target site</th><th>folding energy (in - Kcal/mol)</th><th>heteroduplex</th><th>p value</th></tr>
<tr><td>967. bta-miR-23a MIMAT0003827</td><td>29197</td><td>-10.70</td><td>CAGATTTGCC-AGCTTATGTGAC
|| | || || ||||||
ACCTTTAGGGACCG-TTACACTA</td><td>2.11E-1</td></tr>
<tr><td>968. bta-miR-2410 MIMAT0011968</td><td>29509</td><td>-13.60</td><td>CCAGGGGT--AGAAGAGCTCAA
|| :||| ||||||
AGTGTCCAGGAGGAGTCGAGTC</td><td>7.91E-3</td></tr>
<tr><td>969. bta-miR-98 MIMAT0003809</td><td>30052</td><td>-13.10</td><td>CTGGCAACAGAACCCCTACCTCT
|||: |||||||
TTGTTATGTT-GAATGATGGAGT</td><td>2.94E-1</td></tr>
<tr><td>970. bta-let-7e MIMAT0004333</td><td>30055</td><td>-20.30</td><td>GCAACAGAACC-CCTACCTCT
| |||| ||||||||
TGATATGTTGGAGGATGGAGT</td><td>2.94E-1</td></tr>
<tr><td>971. bta-miR-301a MIMAT0009276</td><td>30215</td><td>-12.10</td><td>GAGGAGCCCCAATAAACAATGCACTG
||||| |||||||
TACGAAACTGTTAT-GATAACGTGAC</td><td>5.91E-3</td></tr>
<tr><td>972. bta-miR-669 MIMAT0013838</td><td>30333</td><td>-20.80</td><td>CTTGCAGA-ACTCGCACCCACA
:||| | | |:||||||||
GTGCGTGTACGTGTGTGGGTGT</td><td>4.98E-2</td></tr>
<tr><td>973. bta-miR-11979 MIMAT0046367</td><td>30341</td><td>-15.70</td><td>ACTCGCACCCACAGCTGGTGCG
|||| | ||||||
TCCTCACTGGTGCCCACCACGT</td><td>4.98E-2</td></tr>
<tr><td>974. bta-miR-2290 MIMAT0011798</td><td>267</td><td>-11.50</td><td>GATGTTTGAGGACGCAGAGGA
:|| || ||||||
TGTTTGGCTGGTG-GTCTCCG</td><td>3.03E-1</td></tr>
<tr><td>975. bta-miR-12058 MIMAT0046765</td><td>3106</td><td>-12.50</td><td>GATGATGACTTTCTTGAAGAAA
|||||| || ||||||
CGTGTACTGA-TGACCTTCTTG</td><td>1.72E-1</td></tr>
<tr><td>976. bta-miR-449b MIMAT0009321</td><td>3450</td><td>-18.50</td><td>GGCAGTGATGCAG-AAACTGCCC
| |||: :||: ||||||
CGGTCG-ATTGTTATGTGACGGA</td><td>1.22E-2</td></tr>
<tr><td>977. bta-miR-2450a MIMAT0012030</td><td>3454</td><td>-15.80</td><td>GTGATGCA--GAAACTGCCCT
||||| |||||||
GTGTACGTGGAGATGACGGGT</td><td>1.22E-2</td></tr>
<tr><td>978. bta-miR-369-3p MIMAT0003802</td><td>5904</td><td>-8.20</td><td>TAAGTATTATACTCAGCGTATTATT
||| ||:| || || ||||||||
TTTC-TAGT-TG-GT-ACATAATAA</td><td>6.48E-2</td></tr>
<tr><td>979. bta-miR-11993 MIMAT0046634</td><td>6064</td><td>-21.10</td><td>ACTGAGTGGCCAACAGCTACAGGTG
|||||| || || ||||||
GTACTCACATGT--GCG-TGTCCAG</td><td>4.26E-2</td></tr>
<tr><td>980. bta-miR-93 MIMAT0003837</td><td>8812</td><td>-12.30</td><td>CACTTTATTACACATGCACTTTC
||| |||||||
ATGGACGTGCTTGT-CGTGAAAC</td><td>1.54E-1</td></tr>
</table>

1. miR Name	leftmost position of predicted target site	folding energy (in -Kcal/mol)	heteroduplex	p value
981. bta-miR-2420 MIMAT0011987	8940	-12.00	CGATGGTAGTC-CACAACCT<br>::||: | |||||||| TTTCTTATTTGTGTGTTGGA	3.62E-1
982. bta-miR-12028 MIMAT0046721	10049	-18.60	ATAAGGTCTACTGTCCCAGACA<br>::|| |||||||||| TCTCTTAG-CCCCAGGGTCTGT	1.75E-1
983. bta-miR-12058 MIMAT0046765	11320	-8.50	GGTTCTAACTTAGAGGAAGAAA<br>||| ||||||| CGTGTACTGA-TGACCTTCTTG	2.73E-1
984. bta-miR-93 MIMAT0003837	11802	-12.20	GTGGCAATATTGTAGCACTTTG<br>:||||||||| ATGGACGTGCTTGTCGTGAAAC	6.2E-2
985. bta-miR-20b MIMAT0003796	11803	-16.00	TGGCAATATTGT-AGCACTTTG<br>:|:||| ||||||||| ATGGACGTGACACTCGTGAAAC	6.2E-2
986. bta-miR-12058 MIMAT0046765	11927	-12.40	GC-C-TGACTAGTATTGAAGAAG<br>|| | |||||| | |||||| CGTGTACTGATGA--CCTTCTTG	1.75E-1
987. bta-miR-380-3p MIMAT0003804	13607	-6.10	TGAACACGATTTCTTTACATT<br>|| ||: | |||||| TCTGCACCTGGTGTAATGTAT	2.73E-1
988. bta-miR-376e MIMAT0025543	13962	-7.30	TGGTATGATTTTGGTGACTATGTT<br>|||||| ||||||| TTACACCTAAAA--GGAGATACAA	3.48E-1
989. bta-miR-381 MIMAT0009307	16270	-6.00	TAAATGATGTT-ACCAAATTGTATC<br>|| |:| || |||||| TGTCTCT-CGAACGG--GAACATAT	1.36E-1
990. bta-miR-2420 MIMAT0011987	16726	-10.80	TGTATTATCGCGCCACAACCA<br>:|:| |||||| TTTCTTATTTGTG-TGTTGGA	8.13E-2
991. bta-miR-92a MIMAT0009383	19305	-13.00	ACTTGC-ATCACAAGATGCAATT<br>|| || ||||| |||||| TGTCCGGCCCTGTTC-ACGTTAT	2.46E-1
992. bta-miR-6525 MIMAT0025554	20639	-12.70	TAAGTATTTGAATTCCCCAA<br>::|| ||||||| GAGTGAGGACGAAAGGGGTC	3.25E-1
993. bta-miR-668-3p MIMAT0040936	21454	-10.40	AAGAAGTTTTTGTTG-GTGACAG<br>||| |::| |||||| CCCATCA--CCCGGCTCACTGTA	1.45E-1
994. bta-miR-20b MIMAT0003796	21522	-6.00	GACAAGC-CT--AATCACTTTA<br>|| || || |||||| ATGGACGTGACACTCGTGAAAC	1.45E-1

1. miR Name	leftmost position of predicted target site	folding energy (in - Kcal/mol)	heteroduplex	p value
995. bta-miR-93 MIMAT0003837	21522	-5.80	GACAAGC-CTAAT--CACTTTA || ||	||: |||||| ATGGACGTGCTTGTCGTGAAAC	1.45E-1
996. bta-miR-92a MIMAT0009383	21942	-5.70	GATTTCATAAACAGCATGCAATT |||: |||||| TGTCCGGCCCTGTT-CACGTTAT	9.19E-2
997. bta-miR-12058 MIMAT0046765	22150	-7.80	GTA-AAGA--GCTAGAAGAAG |:| || :|| |||||| CGTGTACTGATGACCTTCTTG	3.85E-1
998. bta-miR-2450a MIMAT0012030	22520	-21.40	TATATGGACCTTAATCCTGCCCT |:||| ||||: |||||| GTGTACGTGGAG--ATGACGGGT	3.66E-1
999. bta-miR-369-3p MIMAT0003802	22983	-7.00	TTTTGTCGAATACAAAGTATTATG<br>:||:| || ||||||| TTTCTAGTT--GGT-ACATAATAA	3.76E-1
1000. bta-miR-380-3p MIMAT0003804	25243	-9.00	ACTCCCGACCC-CATTACATC || |||||||| TCTGCACCTGGTGTAATGTAT	1.04E-1
1001. bta-miR-769 MIMAT0009376	29215	-13.10	TGACTGTTGCT--AAGGTCTCA<br>:|: |||||||| TCGAGTCTTGGGCCTCCAGAGT	2.11E-1
1002. bta-miR-2290 MIMAT0011798	29624	-9.20	GGAGTTTGAATTTGCAGAGGG<br>::|| |||||| TGTTTGGCT-GGTGGTCTCCG	8.64E-2
1003. bta-miR-6525 MIMAT0025554	30313	-13.70	GTGAC-CCACAGTTCCCCAT | || || ||||||| GAGTGAGGACGAAAGGGGTC	4.98E-2
1004. bta-miR-20b MIMAT0003796	30469	-6.20	GATTTG-ACAGT--ACACTTTC |::|| || || |||||| ATGGACGTGACACTCGTGAAAC	3.29E-1
1005. bta-miR-323b-3p MIMAT0040935	1705	-10.60	GTAGGTTGCAAGGCTATTGTA |||::|	|||||| TCTCCGGCTGGCACATAACAC	3.11E-2
1006. bta-miR-7864 MIMAT0030449	2905	-15.80	ACTGTGGAT--ATGGAGGAAT |||: :|: :|||||| AGACGGTTGGCCTTCTCCTTC	1.36E-1
1007. bta-miR-7864 MIMAT0030449	3145	-11.30	GATGT-AGAAGG-TGAGGAAA ||: || |||||| AGACGGTTGGCCTTCTCCTTC	2.73E-1
1008. bta-miR-2425-5p MIMAT0011994	3453	-14.70	AGT-GATGCAGAAAC-TGCCCTGT ||| ||| ||	||||||| TCAAGTAC--CTAGGCACGGGACC	1.22E-2

1. miR Name	leftmost position of predicted target site	folding energy (in -Kcal/mol)	heteroduplex	p value
1009. bta-miR-11986c MIMAT0046638	3516	-5.50	TAAGCAACAGTATAGTCAGTTG<br>||: | |||||| AGAAAAGGTTTCCTGAGTCAAG	2.58E-1
1010. bta-miR-10184-3p MIMAT0040943	3650	-9.10	CATACTGG--AAATGCATTAAA<br>|| |:||||||| GGTAACCCAAAAGATGTAATTT	3.34E-1
1011. bta-miR-2284v MIMAT0011937	3980	-6.00	CATTTTCAA-TGACTACTTTTG<br>: ||| :|| |||||| TTTGGGGTTTGCT--TGAAAAA	2.11E-1
1012. bta-miR-2284d MIMAT0011836	5796	-8.30	TAAAAATTTGAAACAAACTTTTA<br>|||||::: ||| ||||||| CTTTTTGGG-ATTGCTTGAAAAA	2.39E-2
1013. bta-miR-2284v MIMAT0011937	5799	-10.30	AAATTTGAAACAAACTTTTA<br>|||::: |||| ||||||| TTTGGGGTTTGCTTGAAAAA	2.39E-2
1014. bta-miR-2284d MIMAT0011836	6130	-5.60	GAAAGGGGTTGTATTACTTTTG<br>||||:: :|: |||||| CTTTTTGGGATTGCTTGAAAAA	2.14E-1
1015. bta-miR-2284v MIMAT0011937	6929	-6.00	GGATTAAGAAC--ACTTTTA<br>::|:: :||| |||||| TTTGGGGTTTGCTTGAAAAA	2.28E-1
1016. bta-miR-2284v MIMAT0011937	7547	-6.10	ATAAACCAGGTAATACTTTTA<br>|||:: | |||||| TTTGGGGTTTGCT-TGAAAAA	2.94E-1
1017. bta-miR-323b-3p MIMAT0040935	8944	-7.40	GGTAGTCCACAACCTTATTGTT<br>:| |:| || |||||| TCTCCGGCTG-GCACATAACAC	3.62E-1
1018. bta-miR-2284d MIMAT0011836	9692	-8.50	AAATGGCTCTTAC-TACTTTTA<br>::|:|| || |||||| CTTTTTGGGATTGCTTGAAAAA	1.98E-1
1019. bta-miR-2284v MIMAT0011937	9695	-7.60	TGGCTCT-TAC-TACTTTTA<br>::|:|: || |||||| TTTGGGGTTTGCTTGAAAAA	1.98E-1
1020. bta-miR-12005 MIMAT0046666	9921	-9.50	TGCTTCCGTTT--CAACTTCAT<br>||| :|| ||||||| TGGGAGGGGAATTCCTGAAGTA	7.65E-2
1021. bta-miR-323b-3p MIMAT0040935	9941	-14.00	TCTTGCAATCTG-GTATTGTG<br>|| |:| |||||||| TCTCCGGCTGGCACATAACAC	7.65E-2
1022. bta-miR-323b-3p MIMAT0040935	9981	-16.60	TAAGGTAGAACCATGTATTGTC<br>|||: || || |||||||| TCTCCGGCT-GGCACATAACAC	7.65E-2

1. miR Name	leftmost position of predicted target site	folding energy (in -Kcal/mol)	heteroduplex	p value
1023. bta-miR-208b MIMAT0009262	10165	-13.30	CTAAGCCTTACAGTGATGTCTTAT<br>||:|||| || :||||||| TGTTTGGAA-AACA-AGCAGAATA	3.56E-2
1024. bta-miR-7864 MIMAT0030449	11320	-13.50	GGTTCTAACT--TAGAGGAAG<br>:|||: |||||||| AGACGGTTGGCCTTCTCCTTC	2.73E-1
1025. bta-miR-302a MIMAT0009278	11800	-9.90	TTGTGGCAATATTGTAGCACTT<br>||:|| | ||||||| AGTGATTTTGTACCTTCGTGAA	6.2E-2
1026. bta-miR-11986c MIMAT0046638	11860	-13.90	GCTTTTGAAAAGCTTGCTCAGTTG<br>||||| |||| :||||||| AGAAAAGGTTTC--CTGAGTCAAG	1.67E-1
1027. bta-miR-6715 MIMAT0046369	11911	-15.90	GCTGTGGAT-AGCAAGTGCCTGA<br>||| : |: || ||||||| CGA-GTTTGACCG-GCACGGACA	1.75E-1
1028. bta-miR-323b-3p MIMAT0040935	16296	-8.60	GGTGGTATGTCATATTATTGTG<br>| ||: :| | ||||||| TCTCCG-GCTGGCACATAACAC	1.36E-1
1029. bta-miR-134 MIMAT0009227	16942	-17.60	GACCTCCTGGTACAGGAAAGTCACA<br>||| |||| ||: ||||||| GGTGAG-ACCA-GTT-GGTCAGTGT	1.11E-1
1030. bta-miR-1388-3p MIMAT0013591	17172	-14.50	ACTATAAATGCATTACCTGAGAT<br>:|| ||||||||| ACGCCCGACTGT-TTGGACTCTA	3.39E-1
1031. bta-miR-10180-3p MIMAT0040938	18311	-22.80	GGCTGTGGCTAAAGCTCCTCCTG<br>:|| : || ||:|||||| TCG-TGTCG--CGCGGGGAGGAG	2.5E-1
1032. bta-miR-323b-3p MIMAT0040935	18671	-5.60	TAATCATGATT-TATATTGTA<br>:||:: |||||| TCTCCGGCTGGCACATAACAC	2.36E-2
1033. bta-miR-10180-3p MIMAT0040938	18704	-22.10	AGCACA-TGT-TGCCTCCTC<br>|||||| :|: : ||||||| TCGTGTCGCGCGGGGAGGAG	2.36E-2
1034. bta-miR-1388-3p MIMAT0013591	20025	-16.10	AGCCAATTCGACAG-CCTGAGAG<br>|| ||||: ||||||| ACG-CCCGACTGTTTGGACTCTA	7.09E-2
1035. bta-miR-2308 MIMAT0011820	20048	-16.60	CAGCTCT--AACCAGAGCCCAC<br>:|||| :|||||| AATGAGAGACGACGTTCGGGTT	7.09E-2
1036. bta-miR-11986c MIMAT0046638	20521	-13.40	TTGATTTTAAAGATTTTCAGTTC<br>||::|||| :||||||| AGAAAAGGTTTC-CTGAGTCAAG	3.16E-1

1. miR Name	leftmost position of predicted target site	folding energy (in - Kcal/mol)	heteroduplex	p value
1037. bta-miR-6715 MIMAT0046369	20846	-17.70	TCTTAGGCAGTGGT--TGCCTGC<br>||:|::| |||: |||||| CGAGTTTG--ACCGGCACGGACA	2.78E-1
1038. bta-miR-2457 MIMAT0012042	21198	-16.20	AGTGAAGGGTTTT-TAATTGGCAT<br>||:| ||:|:| :| |||||| TCGC--GCCGAGACGTCAACCGTT	2.01E-1
1039. bta-miR-2285ba MIMAT0046373	21549	-11.59	CCACTTACCCAGTTTGAGGGTTT<br>||| |||: |||||| GGTTTTTCAAGCAAG-TCCCAAA	1.45E-1
1040. bta-miR-2403 MIMAT0011960	22161	-15.70	GAAGAAGGTGATCTTCCCGAG<br>|: ||||||||| TTCCGGTCGATCGAAGGGCTC	3.85E-1
1041. bta-miR-653 MIMAT0009358	22620	-9.60	ACACAGGCGA--AGGTCAACAA<br>||||: || |||||| GTTGTCT-CTAACAAAGTTGTG	1.86E-1
1042. bta-miR-10180-3p MIMAT0040938	26571	-16.30	TGC-TAGTCTGTTTCCTCCTT<br>|| :||: :|:: |||||| TCGTGTCGCGCGG-GGAGGAG	3.46E-2
1043. bta-miR-653 MIMAT0009358	27332	-10.20	TAATGCTGAACATTTCAACAC<br>:||:: || ||||||||| GTTGTCTCTAACAAAGTTGTG	6.88E-2
1044. bta-miR-12051 MIMAT0046754	27468	-14.90	AATGAATAGGT--TACAGGAGG<br>|||:||:| |||||| AGCCTTGTCTACGACGTCCTCA	2.14E-1
1045. bta-miR-1388-3p MIMAT0013591	28112	-18.20	GGACATCTGG-AGACCTGAGAT<br>|||: |:||||||||| ACGCCCGACTGTTTGGACTCTA	3.03E-1
1046. bta-miR-12051 MIMAT0046754	29650	-12.90	GTGTGCCTATTGCACCAGGAGT<br>||| ||||||| AGCCTTGTCTACGACGTCCTCA	2.69E-1
1047. bta-miR-12005 MIMAT0046666	29979	-10.40	GCTCCTAATTCCAGATCTACTTCAC<br>:| ||| :|| |||||| TG-GGA--GGGGAATTCCTGAAGTA	2.01E-1
1048. bta-miR-2366 MIMAT0011904	30298	-15.80	TAAAACTTGGAACTAGTGACCCA<br>||:: || |||||||| GGTCTGGGAGAAGA-CACTGGGT	4.98E-2
1049. bta-miR-29c MIMAT0003829	2236	-13.70	TTTCATGTTTTGCATGGTGCTT<br>:|||:: |||||||| ATTGGCTAAAGTTTACCACGAT	2.61E-1
1050. bta-miR-2399-5p MIMAT0011954	3443	-21.60	CAGTTTTGGCAGTGATGCAGAAAC<br>||||: :|||: |:|||||| ATCAAGGTTGTCG--ATGTCTTTC	1.22E-2

<table>
<tr><th>1. miR Name</th><th>leftmost position of predicted target site</th><th>folding energy (in -Kcal/mol)</th><th>heteroduplex</th><th>p value</th></tr>
<tr><td>1051. bta-miR-2367-5p MIMAT0011909</td><td>4284</td><td>-17.00</td><td>ACCTGATG-C-GAGGACACAGGGT
|||| | ||| |||||||
GAGACTACCGACTC-AATGTCCCA</td><td>1.75E-1</td></tr>
<tr><td>1052. bta-miR-2284m MIMAT0011976</td><td>5798</td><td>-14.00</td><td>AAAATTTGAAACAAACTTTTA
||||:::||| |||||||||
TTTTGGGCTT-GTTTGAAAAG</td><td>2.39E-2</td></tr>
<tr><td>1053. bta-miR-2330-5p MIMAT0011861</td><td>5916</td><td>-14.30</td><td>TCAG-C-GTATTATTAAAGCCCA
:||| | :|:| : |||||||
GGTCAGGAGTGACGGGTTCGGGT</td><td>6.48E-2</td></tr>
<tr><td>1054. bta-miR-197 MIMAT0009257</td><td>6260</td><td>-15.00</td><td>ATGATGTTGACGATGGTGGTGAT
|| || ||||||||
CGACCCACCT-CTTCCACCACTT</td><td>3.52E-1</td></tr>
<tr><td>1055. bta-miR-29c MIMAT0003829</td><td>6787</td><td>-12.30</td><td>AGTGTGGTTGCTAGA-GGTGCTT
:|:|| :|:| ||||||
ATTGGCTAA-AGTTTACCACGAT</td><td>3.52E-1</td></tr>
<tr><td>1056. bta-miR-2362 MIMAT0011900</td><td>6908</td><td>-13.20</td><td>TTGTTGGTAAGATCG-CACAGTG
:|: |||||||
CCAGTCTTGCGAGGTAGTGTCAC</td><td>2.28E-1</td></tr>
<tr><td>1057. bta-miR-2284m MIMAT0011976</td><td>6928</td><td>-5.40</td><td>TGGATTAAGAAC--ACTTTTA
::|: |||| ||||||
TTTTG-GGCTTGTTTGAAAAG</td><td>2.28E-1</td></tr>
<tr><td>1058. bta-miR-2284m MIMAT0011976</td><td>7548</td><td>-6.20</td><td>TAAACCAGGTAATACTTTTA
||| |: ||||||
TTTTGGGCTTGTTTGAAAAG</td><td>2.94E-1</td></tr>
<tr><td>1059. bta-miR-2362 MIMAT0011900</td><td>8530</td><td>-7.60</td><td>TTAATGCCCACTT--ACACAGTA
| ||: ||||||
CCAGTCTTGCGAGGTAGTGTCAC</td><td>5.51E-2</td></tr>
<tr><td>1060. bta-miR-761 MIMAT0009373</td><td>8817</td><td>-11.00</td><td>TATTACACATGCACTTTCTGCTGA
|| |||: ||||||
ACACAGTCAAAGTGG--GACGACG</td><td>1.54E-1</td></tr>
<tr><td>1061. bta-miR-29c MIMAT0003829</td><td>9299</td><td>-14.60</td><td>CTGCTAGTTCCATTGCTGGTGCTA
:| :|| || ||||||||
ATTGGCTAAAGT--TTACCACGAT</td><td>2.28E-1</td></tr>
<tr><td>1062. bta-miR-2284m MIMAT0011976</td><td>9694</td><td>-7.50</td><td>ATGGCTCTTAC-TACTTTTA
::|:| || ||||||
TTTTGGGCTTGTTTGAAAAG</td><td>1.98E-1</td></tr>
<tr><td>1063. bta-miR-2355-5p MIMAT0011891</td><td>9932</td><td>-7.60</td><td>CAACTTCATTC-TTGCAATCTGGT
|:| ||| ||||||
CGTATAACAGGTAAC-ATAGACCC</td><td>7.65E-2</td></tr>
<tr><td>1064. bta-miR-194 MIMAT0009254</td><td>9990</td><td>-9.30</td><td>ACCATGTATTGTCAGTGTTACC
|||::| || ||||||
AGGTGTACCTCAACGACAATGT</td><td>7.65E-2</td></tr>
</table>

<table>
<tr><th>1. miR Name</th><th>leftmost position of predicted target site</th><th>folding energy (in -Kcal/mol)</th><th>heteroduplex</th><th>p value</th></tr>
<tr><td>1065. bta-miR-2330-3p MIMAT0011862</td><td>10303</td><td>-15.04</td><td>AACGGCAAACCACAAGGAGC
 ||||| ||||||
CTGCCGGTCCCCATTCCTCA</td><td>3.62E-1</td></tr>
<tr><td>1066. bta-miR-2285x MIMAT0029946</td><td>10472</td><td>-6.30</td><td>CTGA--TTTCAATGGGGATTTTTA
| | |||| | :|||||||
GGTTTTCAAGT-AAGTCTAAAAAG</td><td>8.51E-2</td></tr>
<tr><td>1067. bta-miR-376b MIMAT0009945</td><td>10691</td><td>-12.00</td><td>TAGATGCTTTAGCTTCTATGAC
 | ||| || |:|||||||
TTGTACCTAAAAGGAGATACTA</td><td>3.07E-1</td></tr>
<tr><td>1068. bta-miR-2355-5p MIMAT0011891</td><td>11832</td><td>-13.90</td><td>AATACTTG-CCACTTCTGATCTGGG
     ||| ||| |||||||
CGTATAACAGGT--AACATAGACCC</td><td>6.2E-2</td></tr>
<tr><td>1069. bta-miR-10165-5p MIMAT0040912</td><td>11969</td><td>-15.10</td><td>ACAATACTGTT--TTGCAGGCT
       :||: :||||||||
CACAGGAGCAGTGGACGTCCGA</td><td>2.39E-1</td></tr>
<tr><td>1070. bta-miR-194 MIMAT0009254</td><td>16174</td><td>-13.00</td><td>TTTGC-TGCAAGTGTTGTTACG
|: | || ||:||||||
AGGTGTACCTCAACGACAATGT</td><td>5.43E-2</td></tr>
<tr><td>1071. bta-miR-2399-5p MIMAT0011954</td><td>17731</td><td>-16.00</td><td>TATATTCA-CAGACTGCAGAAAC
    |:|| ||| ||:||||||
ATCAAGGTTGTC-GATGTCTTTC</td><td>1.09E-1</td></tr>
<tr><td>1072. bta-miR-6516 MIMAT0030443</td><td>18857</td><td>-10.20</td><td>TAAAGCTGCCATG-CTCTGCAAC
    :| :|| || ||||||
CAAGTG-TGG-ACAATGACGTTT</td><td>6.11E-2</td></tr>
<tr><td>1073. bta-miR-376b MIMAT0009945</td><td>18933</td><td>-11.70</td><td>GATTTTGATTTTAAGTTCTATGAT
      |||||| :||||||||
TTGTACCTAAAA--GGAGATACTA</td><td>2.25E-1</td></tr>
<tr><td>1074. bta-miR-197 MIMAT0009257</td><td>19514</td><td>-14.50</td><td>TTATACAGGA-CAGGCTGGTGAA
       ||| ||| |||||||
CGACCCACCTCTTCC-ACCACTT</td><td>1.98E-1</td></tr>
<tr><td>1075. bta-miR-154c MIMAT0025542</td><td>20733</td><td>-11.70</td><td>TATACTCAGTTATGTCAATATCT
     |||:: || ||||||||
TCTCTAGTTGGCAC-GTTATAGA</td><td>1.92E-1</td></tr>
<tr><td>1076. bta-miR-2330-3p MIMAT0011862</td><td>20808</td><td>-14.50</td><td>GCAGGTTCAG-AAAAAGGAGT
   ||: ||| ||||||
CTGCCG-GTCCCCATTCCTCA</td><td>2.61E-1</td></tr>
<tr><td>1077. bta-miR-2362 MIMAT0011900</td><td>21286</td><td>-9.60</td><td>GG--AGAA--ATTC--CACAGTT
|| |||| |:| ||||||
CCAGTCTTGCGAGGTAGTGTCAC</td><td>7.65E-2</td></tr>
<tr><td>1078. bta-miR-29c MIMAT0003829</td><td>21298</td><td>-12.00</td><td>CAGTTTGGAACGGG-GGTGCTT
          |::: ||||||
ATTGGCTAAAGTTTACCACGAT</td><td>7.65E-2</td></tr>
</table>

<table>
<tr><th>1. miR Name</th><th>leftmost position of predicted target site</th><th>folding energy (in -Kcal/mol)</th><th>heteroduplex</th><th>p value</th></tr>
<tr><td>1079. bta-miR-2285ar MIMAT0046337</td><td>21552</td><td>-9.10</td><td>CTTACCCAGTTTG--AGGGTTT
||||:: ||||||
CGGTTTTTCAAGTAGTCCCAAA</td><td>1.45E-1</td></tr>
<tr><td>1080. bta-miR-376b MIMAT0009945</td><td>22098</td><td>-7.40</td><td>CCCACTGATAAGCTAGCTATGAT
|| ||| |: |||||||
TTGTACCTAAAAGG-AGATACTA</td><td>3.48E-1</td></tr>
<tr><td>1081. bta-miR-2362 MIMAT0011900</td><td>25873</td><td>-11.90</td><td>GTTCAAACATGTGATCTCACAGTA
||| |:|: : |||||||
CCAGT-CTTGCGAGGTAGTGTCAC</td><td>1.98E-1</td></tr>
<tr><td>1082. bta-miR-2362 MIMAT0011900</td><td>26141</td><td>-15.40</td><td>ATGCAGCATGT-AAATCACAGTT
||| |:|: ||||||||
CCAGTCTTGCGAGGTAGTGTCAC</td><td>1.78E-1</td></tr>
<tr><td>1083. bta-miR-2285ar MIMAT0046337</td><td>29330</td><td>-13.12</td><td>GCCA-TCAACCCAAAAGGGTTC
|||| || ||||||
CGGTTTTTCAAGTAGTCCCAAA</td><td>3.48E-1</td></tr>
<tr><td>1084. bta-miR-761 MIMAT0009373</td><td>29737</td><td>-19.00</td><td>GCAACCAGCGTCA-ACTGCTGC
||| ||| |||||||
ACACAGTCAAAGTGGGACGACG</td><td>1.11E-1</td></tr>
<tr><td>1085. bta-miR-2399-5p MIMAT0011954</td><td>30173</td><td>-6.90</td><td>CAAAGAAATCAG--ACAGAAAA
||| |||||||
ATCAAGGTTGTCGATGTCTTTC</td><td>5.91E-3</td></tr>
<tr><td>1086. bta-miR-2330-5p MIMAT0011861</td><td>30185</td><td>-14.60</td><td>ACAGAAAATTTTGAATAAGCCCC
||| :|| :||||||
GGTCAGGAGTGACGGGTTCGGGT</td><td>5.91E-3</td></tr>
<tr><td>1087. bta-miR-2362 MIMAT0011900</td><td>30302</td><td>-13.00</td><td>ACTTGGAACTAGTGACC--CACAGTT
| |||| |: || ||||||
CCAGTCTTG--CG-AGGTAGTGTCAC</td><td>4.98E-2</td></tr>
<tr><td>1088. bta-miR-2399-5p MIMAT0011954</td><td>30719</td><td>-8.50</td><td>TACTGAAGACACCT-CAGAAAT
:||| || ||||||
ATCAAGGTTGTCGATGTCTTTC</td><td>2.39E-1</td></tr>
<tr><td>1089. bta-miR-194b-3p MIMAT0040934</td><td>245</td><td>-10.80</td><td>ACTGGGCTCCAGAATTTCCATGG
||| :||||||
GCTAACATTGTCGTTGAGGTACA</td><td>8.01E-2</td></tr>
<tr><td>1090. bta-miR-2898 MIMAT0013856</td><td>319</td><td>-16.30</td><td>GATATAGTATGCTCCACCA
|:|| ||||||||
AGGGGCCGTA-GAGGTGGT</td><td>1.54E-1</td></tr>
<tr><td>1091. bta-miR-450a MIMAT0003834</td><td>325</td><td>-9.32</td><td>GTATGCTCCACCACTGCGCAAAA
:||| ||| |||||||
TATA-ATCCTTGTGTAGCGTTTT</td><td>1.54E-1</td></tr>
<tr><td>1092. bta-miR-2285dj MIMAT0046747</td><td>2944</td><td>-8.20</td><td>GCCATAGA--AGAG-AAACTTTC
|:|:| ||: ||||||||
CCCTGTTTGGACTTGTTTGAAAG</td><td>2.42E-1</td></tr>
</table>

1. miR Name	leftmost position of predicted target site	folding energy (in -Kcal/mol)	heteroduplex	p value
1093. bta-miR-194b-3p MIMAT0040934	2950	-11.40	GAAGAGAAAC--TTTCTCCATGT<br>||| |||||||| GCTAACATTGTCGTTGAGGTACA	2.42E-1
1094. bta-miR-2285ae MIMAT0030448	3094	-11.70	ACAGCTCCTGAAGATGATGACTTTCT<br>|||:| |||| ||||||| CTTTTTGGATT--TACT--TGAAAGA	1.72E-1
1095. bta-miR-544a MIMAT0009346	3445	-11.50	GTTTTGGC-AGTGATGCAGAAA<br>|| :|||||||| CTTGAACGATTTTTACGTCTTA	1.22E-2
1096. my-mir-noheader-provided-33	3448	-12.49	TTGGCAGTGATGCAGAAACTGCCC<br>|||: |||||| GTTAGTCG--ATTAATGTGACGGA	1.22E-2
1097. bta-miR-3613a MIMAT0024574	5125	-6.85	GAATTGCTTGCCTACTACAACA<br>|||: ||||||| CTTGTTTTTTTTTCATGTTGT	9.05E-2
1098. bta-miR-27a-3p MIMAT0003532	5987	-12.00	TAGGAC-ACACCGTCTGTGAT<br>|:|| ||: |||||| GCCTTGAATCGGT-GACACTT	2.94E-1
1099. bta-miR-2422 MIMAT0011989	6172	-14.80	AGCCATGAGC--AAGCTTCCCTCAA<br>|| :| || |:||||||| GAGG--CGTGGAGTC-AGGGGAGTT	3.34E-1
1100. bta-miR-1343-3p MIMAT0011840	8728	-11.90	GTTGTTGCTGTAGTAGACCAGGAT<br>||| |: |||||| CTCTCACG--CCCG--GGGTCCTC	3.52E-1
1101. bta-miR-2285ae MIMAT0030448	8814	-9.50	CTTTATTACACATGCACTTTCT<br>||| ||||||| CTTTTTGGATTTACTTGAAAGA	1.54E-1
1102. bta-miR-2286 MIMAT0011793	8896	-14.90	AGTGGCTGTGTGCTTTC-CTCTGCTTG<br>|::| :| ||:| |:|||||| TAATTGTGTAGC-AAGGCGGGACGAAG	2.69E-1
1103. bta-miR-130b MIMAT0009224	8903	-19.60	GTGTGCTTTCCTCTGCTTGCACTA<br>:||: ||||| || ||||||| TACGGGAAAGTAG--TAACGTGAC	2.69E-1
1104. bta-miR-2285bh MIMAT0046388	8944	-7.00	GG--TAGTCC--AC-AACCTTA<br>:| | || || |||||| TCTTTTTGGGCTTGCTTGGAAA	3.62E-1
1105. bta-miR-2286 MIMAT0011793	10064	-19.20	CCAGACATGT-GATATGTTCTGCTTC<br>:|||::| :|::||||||| TAATTGTGTAGCAAGGCGGGACGAAG	1.75E-1
1106. bta-miR-2301 MIMAT0011812	10295	-8.60	CTGCTTATAACGGCAAACCAC<br>||| : ||||||| TTCTCCCTTTGGT-TTTGGTG	3.62E-1

<table>
<tr><th>1. miR Name</th><th>leftmost position of predicted target site</th><th>folding energy (in -Kcal/mol)</th><th>heteroduplex</th><th>p value</th></tr>
<tr><td>1107. bta-miR-153 MIMAT0009239</td><td>10416</td><td>-13.60</td><td>TGTTAAATTTGTGTATATGCAT
 :|:| |||||| ||||||
CTAGTGAAAACACTGATACGTT</td><td>3.24E-2</td></tr>
<tr><td>1108. bta-miR-1814b MIMAT0011908</td><td>10530</td><td>-12.70</td><td>CGTTCAGGATTATATACAATCT
|: ||::|:|| |||||||
GTTTGTTTTGAT--TTGTTAGA</td><td>8.51E-2</td></tr>
<tr><td>1109. bta-miR-507-3p MIMAT0040915</td><td>11676</td><td>-15.40</td><td>GGGTATTGGAGGTGTGCCAAT
     |: :||: ||||||
AAGTAAGGTTCTGTACGGTTA</td><td>3.07E-1</td></tr>
<tr><td>1110. bta-miR-2286 MIMAT0011793</td><td>12110</td><td>-10.50</td><td>CCTGTAATATTG-CTAAATCTGCTTA
      |:||:| :||||||
TAATTGTGTAGCAAGGCGGGACGAAG</td><td>3.46E-2</td></tr>
<tr><td>1111. bta-miR-6520 MIMAT0025538</td><td>12635</td><td>-7.20</td><td>AGACTTGTAATACACCTACTCAA
   ||: ||| |||||||
CGAGAG-AGACTGT-TATGAGTT</td><td>2.18E-1</td></tr>
<tr><td>1112. bta-miR-7857-3p MIMAT0040925</td><td>13759</td><td>-14.90</td><td>TATATGCT-GGTTG-TGAACAAT
     ||: ||||| |||||||
TTTCTCGGTCCAACCTCTTGTTA</td><td>1.32E-1</td></tr>
<tr><td>1113. bta-miR-12021 MIMAT0046714</td><td>16114</td><td>-7.90</td><td>GCGTGGTCTGCTCTTCTCAA
    ||: |||||||
TCGACCGTCTAAAAAGAGTT</td><td>5.43E-2</td></tr>
<tr><td>1114. bta-miR-2346 MIMAT0011881</td><td>16142</td><td>-12.20</td><td>ACGTTGTGGCAGTTGCATCAGA
      : :| | ||||||
CGGTTTGGTGGAAGTGTAGTCA</td><td>5.43E-2</td></tr>
<tr><td>1115. bta-miR-1343-3p MIMAT0011840</td><td>16243</td><td>-12.70</td><td>ATGTGTGTAACGCACCAGGAT
     ||: || ||||||
CTCTCACG-CCCGGGGTCCTC</td><td>1.36E-1</td></tr>
<tr><td>1116. bta-miR-1814b MIMAT0011908</td><td>16369</td><td>-8.80</td><td>TTGGTCTATATAAACAATCT
          ||||||||||
GTTTGTTTTGATTTGTTAGA</td><td>3.57E-1</td></tr>
<tr><td>1117. bta-miR-7857-3p MIMAT0040925</td><td>16872</td><td>-8.40</td><td>CTTGAGACA--TTTCAGAACAAT
   ||| || ||||||||
TTTCTCGGTCCAACCTCTTGTTA</td><td>2.82E-1</td></tr>
<tr><td>1118. bta-miR-30b-5p MIMAT0003547</td><td>17151</td><td>-6.70</td><td>ACTCGTAAGTA-TGTGTTTACT
        ||| :|||||||
TCGACTCACATCCTACAAATGT</td><td>3.39E-1</td></tr>
<tr><td>1119. bta-miR-544a MIMAT0009346</td><td>17730</td><td>-9.10</td><td>ATATATTCACAGACTGCAGAAA
          |:| |||||||
CTTGAACGATTTTTACGTCTTA</td><td>1.09E-1</td></tr>
<tr><td>1120. bta-miR-568 MIMAT0009350</td><td>19769</td><td>-13.20</td><td>TGGTGTCTGCATGTATACAG
  |||| |:||| ||||||
CACACATATGTAAATATGTA</td><td>2.61E-1</td></tr>
</table>

1. miR Name	leftmost position of predicted target site	folding energy (in - Kcal/mol)	heteroduplex	p value
1121. bta-miR-130b MIMAT0009224	20413	-11.30	GTGGTAGTAAGAGTGTTTGCACTG<br>:|| : :| |||||||| TACGG--GAAAGTAGTAACGTGAC	2.21E-1
1122. bta-miR-3613a MIMAT0024574	20748	-5.80	CAATATCTGAA-TACTACAACA<br>||:| :|| ||||||| CTTGTTTTTTTTTTCATGTTGT	1.92E-1
1123. bta-miR-338 MIMAT0009292	20849	-13.20	TAGGCAGTGGTTGC--CTGCTGGT<br>::||: ::| | |||||| AGTTGTT-TTAGTGACTACGACCT	2.78E-1
1124. bta-miR-544a MIMAT0009346	21111	-16.30	GAGTTTTCTTGGAATGCAGAAT<br>|| || || :|||||||||| CTTGAACGATTTTTACGTCTTA	3.16E-1
1125. bta-miR-2422 MIMAT0011989	21972	-16.60	AAAATCACTATCA-ACCCCTCAT<br>|||: ||| ||||||| GAGGCGTGG-AGTCAGGGGAGTT	9.19E-2
1126. bta-miR-425-3p MIMAT0003833	22159	-13.40	TAGAAGAAGGTGATCTTCCCGAG<br>|| :||: ||||||| CCCGCCT-GTGCTGTAAGGGCTA	3.85E-1
1127. bta-miR-1814b MIMAT0011908	22806	-9.70	ATAATGGCTCTGCACAATCT<br>|| : ||: ||||||| GTTTGTTTTGATTTGTTAGA	3.25E-1
1128. bta-miR-361 MIMAT0003830	23235	-13.40	ATGCC--AGG-CAGTCTGATAA<br>|:|| || |||||||| CATGGGGACCTCTAAGACTATT	1.89E-1
1129. bta-miR-2285bh MIMAT0046388	25596	-9.20	TTATAATAGTTGGCAGAACCTTT<br>::| |||||||| TCTTTTTGGGCTTG-CTTGGAAA	2.65E-1
1130. bta-miR-27a-3p MIMAT0003532	26168	-13.60	AATATGGTAGCTTCTGTGAC<br>||||: |||||| GCCTTGAATCGGTGACACTT	4.38E-2
1131. bta-miR-489 MIMAT0009942	27124	-13.30	GTC-CCTACTAAGT-ATGTCAC<br>||| || || || ||||||| CAGCGGTATATACACTACAGTG	2.14E-1
1132. bta-miR-194b-3p MIMAT0040934	27341	-11.80	ACATTTCAACACCCAACCTCCATGA<br>|||| |||| |||||| GCTAACATTGT-CGTTG-AGGTACA	6.88E-2
1133. bta-miR-1343-3p MIMAT0011840	27683	-14.60	ATTATACTGGACACCAGGAG<br>:|| | ||||||| CTCTCACGCCCGGGGTCCTC	2.21E-1
1134. bta-miR-2285dj MIMAT0046747	28048	-10.90	AGG-TAGACCT--TATAACTTTA<br>|| :|:|||| |||||| CCCTGTTTGGACTTGTTTGAAAG	2.5E-1

<table>
<tr><th>1. miR Name</th><th>leftmost position of predicted target site</th><th>folding energy (in - Kcal/mol)</th><th>heteroduplex</th><th>p value</th></tr>
<tr><td>1135. bta-miR-507-3p MIMAT0040915</td><td>28068</td><td>-7.84</td><td>AGCATT--ATTAATTGCCAAA
|||| ||||||
AAGTAAGGTTCTGTACGGTTA</td><td>2.5E-1</td></tr>
<tr><td>1136. bta-miR-2285b MIMAT0011833</td><td>29181</td><td>-7.10</td><td>ACTGGCTATTCTTTGTCAGATTTG
||| ||||||||
GGTTTTTCAAG--TGAGTCTAAAA</td><td>2.11E-1</td></tr>
<tr><td>1137. bta-miR-2285di MIMAT0046746</td><td>29330</td><td>-12.60</td><td>GCCA--TCAACCCAAAAGGGTTC
:||| || ||||||
TGGTTTTACAAGGAAGTCCCAAA</td><td>3.48E-1</td></tr>
<tr><td>1138. bta-miR-2285dj MIMAT0046747</td><td>29370</td><td>-6.90</td><td>TTGAGAAATAATATCTAAACTTTA
|| |||:   :|||||||
CCCTGTTTG-GACTTGTTTGAAAG</td><td>3.85E-1</td></tr>
<tr><td>1139. bta-miR-2346 MIMAT0011881</td><td>29541</td><td>-13.00</td><td>GCTACTTCTCAGCTAC-CATCAGG
||:| | || || | ||||||
CGGT-TTG-GTGGAAGTGTAGTCA</td><td>7.91E-3</td></tr>
<tr><td>1140. bta-miR-196b MIMAT0009256</td><td>30050</td><td>-21.20</td><td>TTCTGGCAACAGAACCCCTACCTC
|:| :|||||| ||||||
AGGGTTGTTGTC-CTTTGATGGAT</td><td>2.94E-1</td></tr>
<tr><td>1141. bta-let-7f MIMAT0003519</td><td>30052</td><td>-12.40</td><td>CTGGCAACAGAACCCCTACCTCT
|||: | |||||||
TTGATATGTT-AGATGATGGAGT</td><td>2.94E-1</td></tr>
<tr><td>1142. bta-miR-507-3p MIMAT0040915</td><td>30157</td><td>-11.70</td><td>TAACTAAGCAGAC-TGCCAAA
|||| ||||||
AAGTAAGGTTCTGTACGGTTA</td><td>5.91E-3</td></tr>
<tr><td>1143. bta-miR-130b MIMAT0009224</td><td>30218</td><td>-17.02</td><td>GAGCCCCAATAAACAATGCACTG
|||| || |||||||
TACGGG-AAAGTAGTAACGTGAC</td><td>5.91E-3</td></tr>
<tr><td>1144. bta-miR-544a MIMAT0009346</td><td>30383</td><td>-14.80</td><td>AGAGTTGGCCAAAGTGCAGAAT
:| ||| |||:||||||||
CTTGAACGATTTTTACGTCTTA</td><td>1.09E-1</td></tr>
<tr><td>1145. bta-miR-2285b MIMAT0011833</td><td>30453</td><td>-6.60</td><td>TATAATGGTGCAATTAGATTTG
|| || |:||||||
GGTTTTTCAAGTGAGTCTAAAA</td><td>3.29E-1</td></tr>
<tr><td>1146. bta-miR-2285ae MIMAT0030448</td><td>30465</td><td>-5.70</td><td>ATTAGATTTGACAGTACACTTTCA
|:|::|:| ||||||
CTTTTTGGATT--TACTTGAAAGA</td><td>3.29E-1</td></tr>
<tr><td>1147. bta-miR-2301 MIMAT0011812</td><td>30557</td><td>-15.70</td><td>GAATATGAGTCCAAAACCAC
||: ||||||||||
TTCTCCCTTTGGTTTTGGTG</td><td>3.66E-2</td></tr>
<tr><td>1148. bta-miR-2346 MIMAT0011881</td><td>30671</td><td>-13.60</td><td>GTTGA-CTGCAGAGGACATCAGC
|: | | | |||||||
CGGTTTGGTG-GAAGTGTAGTCA</td><td>1.28E-1</td></tr>
</table>

1. miR Name	leftmost position of predicted target site	folding energy (in -Kcal/mol)	heteroduplex	p value
1149. bta-miR-2285bh MIMAT0046388	30734	-17.50	AGAAATATAAGAGAATGAACCTTA<br>||||| |: |||:||||||| TCTTTTTG--GGCTTGCTTGGAAA	2.39E-1
